# Wild-Type U2AF1 Antagonizes the Splicing Program Characteristic of U2AF1-Mutant Tumors and Is Required for Cell Survival

**DOI:** 10.1371/journal.pgen.1006384

**Published:** 2016-10-24

**Authors:** Dennis Liang Fei, Hayley Motowski, Rakesh Chatrikhi, Sameer Prasad, Jovian Yu, Shaojian Gao, Clara L. Kielkopf, Robert K. Bradley, Harold Varmus

**Affiliations:** 1 Cancer Biology Section, Cancer Genetics Branch, National Human Genome Research Institute, Bethesda, United States Of America; 2 Department of Medicine, Meyer Cancer Center, Weill Cornell Medicine, New York, United States Of America; 3 Department of Biochemistry and Biophysics, Center for RNA Biology, University of Rochester Medical Center, Rochester, United States Of America; 4 Genetics Branch, National Cancer Institute, Bethesda, United States Of America; 5 Computational Biology Program, Public Health Sciences Division, Fred Hutchinson Cancer Research Center, Seattle, United States Of America; 6 Basic Sciences Division, Fred Hutchinson Cancer Research Center, Seattle, United States Of America; Cincinnati Children's Hospital Medical Center, UNITED STATES

## Abstract

We have asked how the common S34F mutation in the splicing factor U2AF1 regulates alternative splicing in lung cancer, and why wild-type U2AF1 is retained in cancers with this mutation. A human lung epithelial cell line was genetically modified so that *U2AF1*S34F is expressed from one of the two endogenous *U2AF1* loci. By altering levels of mutant or wild-type U2AF1 in this cell line and by analyzing published data on human lung adenocarcinomas, we show that S34F-associated changes in alternative splicing are proportional to the ratio of S34F:wild-type gene products and not to absolute levels of either the mutant or wild-type factor. Preferential recognition of specific 3′ splice sites in S34F-expressing cells is largely explained by differential *in vitro* RNA-binding affinities of mutant versus wild-type U2AF1 for those same 3′ splice sites. Finally, we show that lung adenocarcinoma cell lines bearing *U2AF1* mutations do not require the mutant protein for growth *in vitro* or *in vivo*. In contrast, wild-type U2AF1 is required for survival, regardless of whether cells carry the *U2AF1*S34F allele. Our results provide mechanistic explanations of the magnitude of splicing changes observed in *U2AF1*-mutant cells and why tumors harboring *U2AF1* mutations always retain an expressed copy of the wild-type allele.

## Author Summary

Large-scale genomics studies have identified recurrent mutations in many genes that fall outside the conventional domain of proto-oncogenes. They include genes encoding factors that mediate RNA splicing; mutations affecting four of these genes are present in up to half of proliferative myeloid disorders and in a significant number of solid tumors, including lung adenocarcinoma. Here we have characterized several properties of a common mutant version of the U2AF1 splicing factor, a component of the U2 auxiliary factor complex, in lung cells. We have found that mutant-associated changes in splice site selection are primarily influenced by the ratio of mutant and wild-type U2AF1 gene products; thus increasing wild-type U2AF1 levels represses the mutant-induced splicing program. We show that the altered splice site preferences of mutant U2AF1 can be attributed to changes in its binding to relevant 3′ splice sites. We also show that mutant U2AF1 is different from some oncogenes: the growth properties of lung cancer cell lines carrying the mutant allele are unaffected by loss of the mutant gene, while the wild-type allele is absolutely required for survival. These results advance our understanding of the molecular determinants of the mutant-associated splicing program, and they highlight previously unappreciated roles of wild-type U2AF1 in the presence of the recurrent *U2AF1*S34F mutation.

## Introduction

Somatic mutations in genes encoding four splicing factors (*U2AF1*, *SF3B1*, *SRSF2* and *ZRSR2*) have recently been reported in up to 50% of myelodysplastic syndromes (MDS) and related neoplasms and at lower frequencies in a variety of solid tumors [1–9]. Among these factors, only *U2AF1* is known to be recurrently mutated in lung adenocarcinomas (LUADs) [3,9]. The only recurrent missense mutation of *U2AF1* in LUAD affects codon 34 and always changes the conserved serine in a zinc knuckle motif to phenylalanine (p.Ser34Phe, or S34F). This striking mutational consistency suggests a critical, yet unknown, role for *U2AF1*S34F during lung carcinogenesis. In addition, the wild-type (WT) *U2AF1* allele is always retained in cancers with common *U2AF1* mutations, including *U2AF1*S34F [2]. However, the functional significance of the wild-type allele in cells with mutant *U2AF1* is not known.

U2AF1 is a component of the U2 small nuclear ribonucleoprotein auxiliary factor complex (U2AF) [10,11]. During early spliceosome assembly, U2AF recognizes sequences at the 3′ ends of introns to facilitate the recruitment of the U2 small nuclear ribonucleoprotein (snRNP) complex to the 3′ splice site; the recruitment occurs in conjunction with recognition of the intronic branch point by splicing factor 1 (SF1) [12,13]. *In vitro* crosslinking assays showed that U2AF1 contacts the AG dinucleotide at the intron-exon boundary and flanking sequences [14–16].

Consistent with the critical role that U2AF1 plays in RNA splicing, *U2AF1* mutations are known to cause specific alterations in RNA splicing, most notably affecting the inclusion of cassette exons in mRNA [17–20]. However, the precise molecular basis of these splicing alterations, as well as how they are quantitatively regulated, is unknown. One possibility is that U2AF1 mutations cause altered RNA-binding affinity, resulting in altered splice site recognition. A computational model of the structure of the U2AF1:RNA complex suggested that Ser34 is a critical residue that contacts RNA [17]. Another study reported that U2AF1S34F exhibited altered affinity relative to the wild-type protein for RNA oligonucleotides derived from a cassette exon whose recognition is repressed in S34F-expressing cells [18]. Finally, the S34F mutation reportedly prevented a minimal fission yeast U2AF heterodimer from binding to a particular 3′ splice site RNA sequence [21]. However, it is not known whether altered RNA binding accounts for most S34F-associated splicing alterations and whether mechanisms other than altered binding control S34F-associated splicing.

Here, we combine genetic and biochemical approaches to show that wild-type U2AF1 antagonizes the S34F-associated splicing program in lung epithelial cells. Analyses of the transcriptomes of primary LUAD samples as well as isogenic lung cells in culture indicate that the ratio of mutant to wild-type U2AF1 gene products is a critical determinant of the magnitude of S34F-associated changes in alternative splicing. S34F-associated splicing alterations can be largely explained by differences in the relative affinities of U2AF-SF1 complexes containing mutant versus wild-type U2AF1 for RNA containing the relevant 3′ splice sites. Moreover, we show that proliferation of cancer cells with *U2AF1*S34F is critically dependent on expression of the wild-type, but not the mutant, allele of *U2AF1*.

## Results

### S34F-associated splicing correlated with S34F:WT mRNA ratios in LUAD

**Fig 1:**
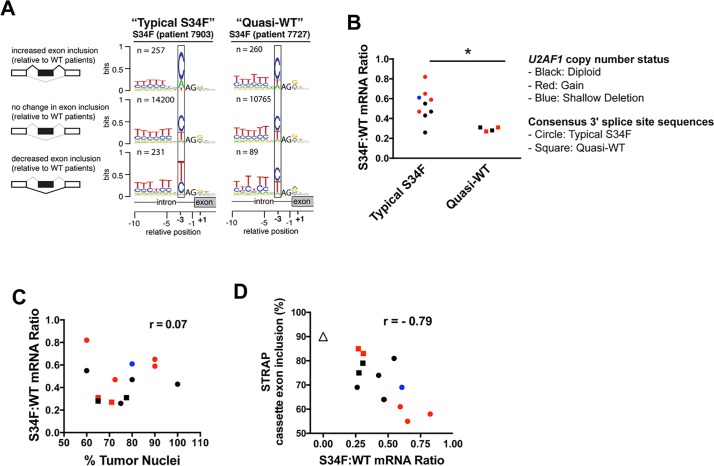
S34F-associated splicing program correlates with S34F:WT mRNA ratios in LUAD. **(A)**. Different consensus 3′ splice site sequences preceding cassette exons for two representative LUADs with the S34F mutation computed from published TCGA transcriptomes. In both cases, changes in the use of cassette exons were determined by comparisons with an average transcriptome from LUADs without *U2AF1* mutations. Boxes highlight nucleotides found preferentially at the -3 position. The nucleotide frequencies preceding exons that are more often included or more often excluded in tumors with the S34F mutation differ from the genomic consensus in the tumor from patient #7903, which represent “typical S34F” consensus 3′ splice sites, but not in the tumor from patient #7727, which represent “quasi-WT” consensus 3′ splice sites. (Consensus 3′ splice sites from transcriptomes of every S34F-mutant LUAD are presented in S1 Fig). The analysis was restricted to introns with canonical GT-AG U2-type splice sites. The invariable AG at the 3′ splice site was not plotted to scale in order to highlight the consensus sequences at the -3 position. The vertical axis represents the information content in bits (the maximal value is two. Zero to one bit is shown). n is the number of cassette exon sequences used to construct the logo. A cartoon illustrating alternative splicing of a cassette exon (black box) is shown on the left side of the sequence logo. Black lines over splice junctions illustrate the S34F-promoted isoform for each comparison. **(B)**. *U2AF1*-mutant LUAD transcriptomes harboring “typical S34F” consensus 3′ splice sites have relatively high S34F:WT mRNA ratios. *U2AF1*-mutant LUAD samples were grouped based on the nature of the consensus 3′ splice sites. The asterisk represents a statistically significant change by Student’s t test. **(C)**. S34F:WT *U2AF1* mRNA ratios do not correlate with tumor purity in LUAD tumors with the S34F mutation. Tumor purity is represented by the percent of tumor nuclei in each LUAD sample (derived from TCGA clinical data) and plotted against the S34F:WT mRNA ratios. **(D)**. Inclusion of the *STRAP* cassette exon correlates with the S34F:WT mRNA ratio. Same as Panel **C** but the S34F:WT mRNA ratio is plotted against the inclusion frequency for the *STRAP* cassette exon. The median inclusion level of the same cassette exon for all transcriptomes from tumors without a *U2AF1* mutation (S34F:WT mRNA ratio = zero) is shown as a triangle. r, Pearson’s correlation coefficient. In panels B–D, circles represent samples with typical-S34F consensus 3′ splice site sequences; squares represent samples with quasi-WT consensus 3′ splice site sequences. Colors indicate *U2AF1* copy number status as calculated by GISTIC 2.0 (See details in the Supplemental Materials and Methods): black, diploid; blue, shallow deletion; red, gain.

Before undertaking experiments to study the effects of mutant U2AF1 in cultured cells, we first examined 512 transcriptomes from primary human LUADs published by The Cancer Genome Atlas (TCGA) [9]. Thirteen of these tumors harbor the most common *U2AF1* mutation, S34F. (Two others carry rare mutations of unknown significance, S65L and G216R, and were not considered further.) We identified cassette exons whose inclusion was increased or decreased by ten percent or more in each tumor with the S34F mutation relative to the median inclusion of each cassette exon across all tumors without a *U2AF1* mutation. We then identified consensus sequence motifs for the 3′ splice sites lying immediately upstream of the cassette exons that were promoted or repressed in association with *U2AF1*S34F, represented by “sequence logos” as shown in Figs 1A and S1. The same analysis was performed on 19 random tumors lacking a *U2AF1* mutation as controls.

As illustrated by data from patient 7903 in Fig 1A, over two hundred cassette exons were included more frequently and a similar number were included less frequently in this *U2AF1*-mutant tumor. Notably, as illustrated by the sequence logos, the nucleotide distribution at the -3 position (boxed) of promoted and repressed exons was different from that observed upstream of the much larger number of unaffected exons: A replaced T as the second most common nucleotide preceding the promoted exons, while T was more common than C in the sequence preceding the repressed exons. These patterns were observed in nine of the thirteen tumors with the *U2AF1*S34F allele (S1A Fig). They have been observed previously in comparisons between transcriptomes carrying the *U2AF1*S34F allele with wild-type transcriptomes [17–20], and are therefore henceforth referred to as the “typical S34F” consensus 3′ splice sites. In the other four tumors with the *U2AF1*S34F mutation, these “typical S34F” consensus 3′ splice sites were partially or completely absent (Figs 1A and S1B). Those four tumors exhibited consensus 3′ splice sites that were similar to the consensus 3′ splice sites identified in tumors lacking a *U2AF1* mutation, where variations in inclusion relative to the median wild-type sample are presumably stochastic (S1C Fig). Thus, we henceforth refer to these sequence patterns, as shown for tumor from patient 7727 in Fig 1A, as “quasi-WT”.

To explain why transcriptomes of some tumors with *U2AF1* mutations showed a typical S34F-associated consensus 3′ splice sites, while others exhibited quasi-WT patterns, we estimated the levels of mutant and total *U2AF1* mRNA based on available data from the tumors to determine the ratio of mutant to wild-type (S34F:WT) mRNA. Tumors with quasi-WT patterns had low S34F:WT mRNA ratios (ranging from 0.27 to 0.31), whereas all but one tumor with the S34F-associated pattern had higher ratios (0.43 or more) (Fig 1B). In contrast, absolute levels of *U2AF1*S34F mRNA or total *U2AF1* mRNA levels were not different between these two groups of tumors (S2 Fig, panels A and B).

We next sought to understand the origin of the wide range of S34F:WT ratios that we observed. These ratios, ranging from 0.26 to 0.82, could not be explained by contamination of tumor cells with non-tumor cells, since the proportion of tumor nuclei reported for these samples did not correlate with S34F:WT mRNA ratios (Fig 1C) or with the presence or absence of the expected S34F-associated consensus 3′ splice sites (S2C Fig). Conversely, *U2AF1* DNA copy number correlated with the estimated levels of total *U2AF1* mRNA (S2D Fig). Seven of the 13 *U2AF1*S34F mutant samples, including two of the four samples displaying quasi-WT consensus 3′ splice site sequences, showed either copy number loss or gain at the *U2AF1* locus, suggesting that copy number variation (CNV) might account, at least in part, for the varying S34F:WT mRNA ratios in LUAD samples (Fig 1B). Unbalanced allelic expression or proportions of tumor subclones might also contribute to the variable S34F:WT mRNA ratios, although these possibilities could not be readily tested using the available LUAD data.

We next tested whether the *U2AF1* S34F:WT ratio was associated with quantitative changes in splicing (versus the qualitative differences in consensus 3′ splice sites described above). We correlated S34F:WT ratios with the quantitative inclusion of specific S34F-responsive cassette exons and 5' extensions of exons resulting from competing 3′ splice sites that were reported previously [17,19,20]. We studied three cassette exons that exhibited less (*ASUN* and *STRAP*) or more (*ATR*) inclusion, and two 5' extensions of exons (*FMR1* and *CASP8*) that were used less frequently in the presence of *U2AF1*S34F. Tumors with the highest S34F:WT mRNA ratios showed the lowest inclusion levels of the cassette exon in *STRAP* mRNA, whereas tumors without a *U2AF1* mutation had the highest level of inclusion (Fig 1D; Pearson correlation of -0.79). Similar correlations were observed between inclusion levels of other representative exons and S34F:WT mRNA ratios (S3 Fig, panels E, I, M and Q). As controls, we tested for correlations between the inclusion of these cassette exons and levels of *U2AF1*S34F mRNA, total *U2AF1* mRNA, or percent tumor nuclei. None of these analyses, with the exception of the *U2AF1*S34F mRNA level versus the inclusion of the 5' extension of the *FMR1* exon, showed a relationship as strong as that observed for the S34F:WT mRNA ratio (S3 Fig panels B-D, F-H, J-L, N-P and R-T). These results indicate that the S34F:WT mRNA ratio predicts the magnitude of S34F-associated splicing in human LUAD.

### Creation of isogenic lung cell lines that recapitulated features of S34F-associated splicing in LUAD

The results presented in the preceding section, based on analyses of LUAD tumors with the *U2AF1*S34F mutation, suggest that the magnitude of S34F-associated splicing is a function of the S34F:WT mRNA ratio. To directly test this hypothesis, we developed a cell line that allows manipulation of WT and mutant U2AF1 gene product levels and measurement of the corresponding effects on RNA splicing.

**Fig 2:**
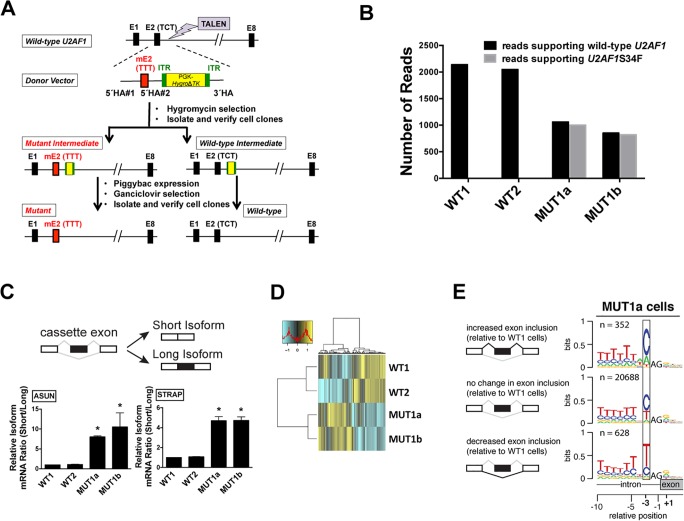
Creation of isogenic lung cell lines that recapitulate features of S34F-associated splicing. **(A)**. Strategy to create a TCT to TTT point mutation (S34F) at the endogenous *U2AF1* locus in HBEC3kt cells. TALEN, transcription activator-like effector nuclease; E, exon; mE, mutant (S34F) exon; ITR, inverted terminal repeat; HA, homology arm. See Results and Supplemental Materials and Methods for details. **(B)**. MUT1a and MUT1b cells contain similar levels of mutant and wild-type *U2AF1* mRNA. The number of reads supporting mutant or wild-type *U2AF1* was obtained from RNA-seq, using poly(A)-selected RNA from the four cell lines. **(C).** The S34F-associated cassette exons in *ASUN* and *STRAP* mRNAs show decreased inclusion in MUT cell lines. (Top) Scheme of alternative splicing with a cassette exon (black box) to generate short and long isoforms in which the cassette exon is excluded or included. (Bottom) Alternative splicing of cassette exons in *ASUN* and *STRAP* mRNAs, measured by RT-qPCR using isoform-specific primers. The short/long isoform ratio in WT1 cells was arbitrarily set to 1 for comparison. Asterisks represent statistical significant changes as compared to that in WT1 cells. Error bars represent s.e.m. (standard error of the mean) (n = 4). **(D)**. Heat map depicting the inclusion levels of all cassette exons that showed at least a 10% change in use among the cell lines. The dendrogram was constructed from an unsupervised cluster analysis. **(E).** Sequence logos from 3′ splice sites preceding cassette exons with altered inclusion in MUT1a cells display typical S34F consensus 3′ splice sites. Logos were constructed as in Fig 1A based on the transcriptome of MUT1a cells in comparison with that of WT1 cells. Other comparisons of transcriptomes from MUT and WT cell lines yielded similar sequence logos.

The human bronchial epithelial cell line (HBEC3kt) was previously derived from normal human bronchial tissue and immortalized by introduction of expression vectors encoding human telomerase reverse transcriptase (*hTERT*) and cyclin-dependent kinase-4 (*CDK4*) [22]. To knock in a *U2AF1*S34F allele at an endogenous locus in HBEC3kt cells, we adopted a published genomic DNA editing approach [23], using the *PiggyBac* transposon that leaves no traces of exogenous DNA at the locus (Fig 2A; see Supplemental Materials and Methods for details). We identified three cell clones at intermediate stages of gene editing after screening more than 50 primary transfectants (S4 Fig, panels A and B). Sanger sequencing of these intermediate clones revealed that one of the three clones carried the desired S34F missense sequence, while two clones were wild-type (S4C Fig). Wild-type intermediates are expected because a homologous sequence between the S34F point mutation and the drug cassette in the vector can serve as the 5' homology arm for recombination (designated as 5' HA#2 in Fig 2A). From the final clones derived from these intermediates (after transposition to remove the drug cassette flanked by the *PiggyBac* elements), we chose one subclone from each of the two wild-type intermediate clones (referred to as WT1 and WT2 cells) and two subclones from the sole mutant intermediate clone (referred to as MUT1a and MUT1b cells) for all subsequent experiments with isogenic HBEC3kt cells (S4D Fig). The MUT and WT cells all expressed similar levels of *U2AF1* mRNA and protein (S4E Fig). Using high-throughput mRNA sequencing (RNA-seq) and allele-specific RT-qPCR, we observed similar levels of wild-type and mutant *U2AF1* mRNAs in the MUT cells (Figs 2B and S5C), consistent with heterozygosity at the *U2AF1* locus.

To determine how the engineered *U2AF1 S34F* allele affects mRNA splicing, we first assayed the inclusion levels of 20 cassette exons that were previously reported to be associated with mutant *U2AF1* in both LUAD and AML (acute myeloid leukemia) [19]. We confirmed that all 20 of these cassette exons, which included the previously studied *ASUN* and *STRAP* cassette exons, were indeed S34F-dependent in our engineered cells using RT-qPCR with isoform-specific primers (Figs 2C and S6).

We next evaluated the global difference in cassette exon recognition in MUT versus WT cells using RNA-seq (S1 Table). MUT and WT cells were grouped separately in an unsupervised cluster analysis based on cassette exon inclusion (Fig 2D). We observed the expected consensus 3′ splice sites of cassette exons that were promoted or repressed in MUT versus WT cells (Fig 2E). Overall, these results indicate that we successfully created clonal HBEC3kt cells isogenic for *U2AF1*S34F and that the MUT cells exhibited similar alterations in splicing relative to their WT counterparts as do primary LUAD transcriptomes.

### The ratio of S34F:WT gene products controlled S34F-associated splicing in isogenic lung cells

We next used our isogenic cell model with the *U2AF1*S34F mutation to test the hypothesis that the S34F:WT ratio, rather than absolute levels of the mutant or wild-type gene products, controls S34F-associated splicing. We tested two specific predictions. First, changing the levels of *U2AF1*S34F while keeping the S34F:WT ratio constant should not affect S34F-associated splicing. Second, changing the level of wild-type *U2AF1* while keeping the level of *U2AF1*S34F constant (e.g., allowing the S34F:WT ratio to change) should alter the inclusion of S34F-dependent cassette exons. We tested these predictions in the isogenic HBEC3kt cells by manipulating levels of wild-type or mutant *U2AF1* gene products and measuring the subsequent changes in S34F-associated splicing.

**Fig 3:**
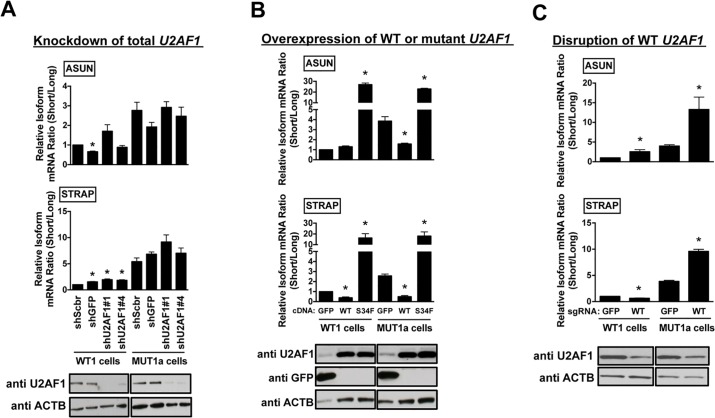
The ratio of S34F:WT U2AF1 gene products controls S34F-associated splicing in isogenic HBEC3kt cell lines. **(A)**. Reduction of both mutant and wild-type U2AF1 RNA and protein does not affect S34F-associated splicing. WT1 and MUT1a cells were transduced with shRNAs against *U2AF1* (shU2AF1#1 and #4) or two control shRNAs (shScbr, a scrambled shRNA; shGFP, an shRNA against *GFP*). Total RNA and protein were harvested 4 days later. The frequencies of incorporation of cassette exon sequences in *ASUN* and *STRAP* mRNAs (top and middle panels) were determined by the relative short/long isoform ratios by RT-qPCR, as represented in Fig 2C. Immunoblots for U2AF1 and ACTB in total cell lysates are shown in the bottom panel. Asterisks represent statistical significant changes as compared to shScbr-transduced condition in each cell line. Error bars represent s.e.m (n = 3). **(B)**. Overexpression of wild-type or mutant *U2AF1* to change S34F:WT ratios alters S34F-sensitive splicing. WT1 and MUT1a cells were transduced with expression vectors encoding *GFP*, wild-type (WT) or mutant (S34F) *U2AF1* for 3 days before harvesting cells to quantify the level of splicing changes and proteins as in panel A. Asterisks represent statistical significant changes as compared to GFP-transduced condition in each cell line. Error bars represent s.e.m (n = 3). **(C)**. Disruption of WT *U2AF1* by gene editing to increase S34F:WT ratios enhances S34F-sensitive splicing. WT1 and MUT1a cells were transduced with Cas9 and either sgRNA-GFP or sgRNA-WT. Total RNA and protein were harvested 6 days later for assays as in panel **A**. Asterisks represent statistical significant changes as compared to Cas9 and sgRNA-GFP-transduced condition in each cell line. Error bars represent s.e.m. (n = 3).

We first reduced the amounts of both mutant and wild-type *U2AF1* mRNA concordantly in MUT1a cells, keeping the S34F:WT mRNA ratio constant. This was achieved by transducing MUT1a cells with short hairpin RNAs (shRNAs) that target regions of the *U2AF1* transcripts distant from the S34F missense mutation. The same shRNAs were also introduced in WT1 cells as a control. Allele-sensitive RT-qPCR confirmed that the mRNA ratio of the mutant and wild-type *U2AF1* remained constant (S7A Fig), while the overall U2AF1 mRNA and protein levels were reduced by more than 90% (Figs 3A, bottom panel, and S7B). Knockdown of total *U2AF1* in both MUT1a and WT1 cells did not cause significant changes in recognition of the *ASUN* or *STRAP* cassette exons, two splicing events that are strongly associated with *U2AF1*S34F, in either cell line (Fig 3A, upper panels). Similar results were obtained for two additional S34F-associated cassette exons in *USP25* and *AXL* that exhibit increased inclusion in cells expressing *U2AF1*S34F (S7 Fig, panels C and D).

We next confirmed that knockdown of *U2AF1* was sufficient to alter splicing events known to be dependent on wild-type U2AF1. We studied a competing 3′ splice site event in *SLC35C2*, in which the use of an intron-proximal over an intron-distal 3′ splice site depends on the level of U2AF1 independent of a *U2AF1* mutation [24]. Knockdown of total *U2AF1* in either WT1 or MUT1a cell lines reduced the use of the U2AF1-dependent intron-proximal 3′ splice site (S7E Fig). Thus, reduction of *U2AF1* to levels sufficient to affect U2AF1-dependent alternative splicing did not affect S34F-associated splicing in MUT1a cells when the S34F:WT ratio was maintained.

We next altered the S34F:WT ratio by separately overexpressing mutant or wild-type *U2AF1* in WT1 and MUT1a cells and examining the subsequent changes in the recognition of the *ASUN* and *STRAP* cassette exons. These cassette exons are preferentially excluded in cells expressing *U2AF1*S34F. Increasing the amount of U2AF1S34F protein—hence increasing the S34F:WT ratio in either cell type—further enhanced skipping of these cassette exons (Fig 3B). Conversely, decreasing the S34F:WT ratio in MUT1a cells by increasing the production of wild-type U2AF1 protein reduced the extent of exon skipping to approximately the same levels seen in WT1 cells (Fig 3B).

We also altered the production of wild-type U2AF1 mRNA and protein in MUT1a cells by disrupting the endogenous wild-type *U2AF1* locus with the CRISPR-Cas9 system. Single-guide RNAs (sgRNAs) designed to match either the WT or mutant *U2AF1* sequences were shown to disrupt either reading frame selectively, generating indels (insertions and deletions) at the *U2AF1* locus and thereby changing the S34F:WT ratios (S8 and S9 Figs). Since WT *U2AF1* is required for the growth of these cells (as shown below), we extracted RNA and protein from cells six days after transduction with Cas9 and sgRNA-WT, when depletion of wild-type U2AF1 was incomplete (Fig 3C). Selective disruption of wild-type *U2AF1* increased the S34F:WT mRNA ratio in MUT1a cells; as predicted, the extent of exon skipping was further increased in *ASUN* and *STRAP* mRNAs (Fig 3C). Notably, the degree of exon skipping induced by mutant cDNA was similar to that caused by disrupting the wild-type *U2AF1* allele (compare Fig 3C with 3B), even though the absolute protein levels of U2AF1S34F were different in the two experiments. These results show that wild-type U2AF1 antagonizes the activity of U2AF1S34F by a competitive mechanism, such that the S34F:WT ratio controls the magnitude of S34F-associated splicing changes independent of levels of either mutant or wild-type protein.

### Disruption of WT U2AF1 globally enhanced S34F-associated splicing

**Fig 4:**
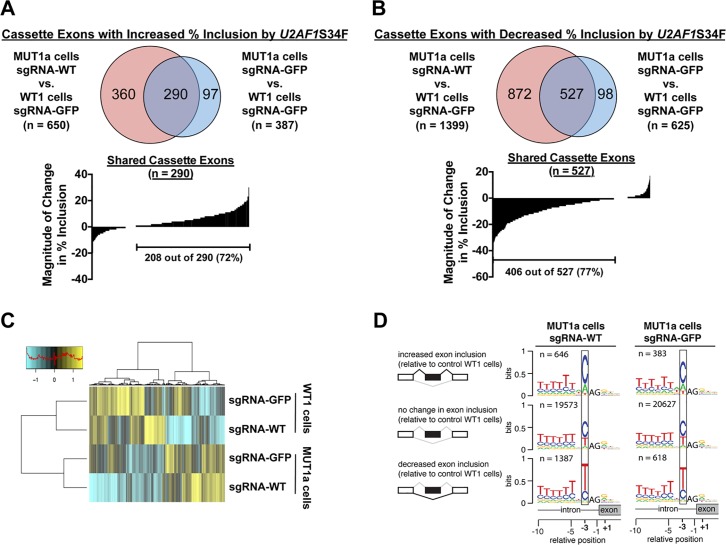
Increasing the ratio of S34F:WT gene products by disrupting the wild-type *U2AF1* locus enhances S34F-associated splicing of cassette exons. **(A)**. RNA-seq was performed for WT1 and MUT1a cells transduced with Cas9 and either sgRNA-GFP or sgRNA-WT. Upper panel: The Venn diagrams show overlap of 290 cassette exons that display at least a ten percent increase in inclusion levels in MUT1a cells relative to levels in WT1 cells, with or without CRISPR-Cas9-mediated disruption of wild-type *U2AF1*. Lower panel: Increasing the S34F:WT ratio by disrupting wild-type *U2AF1* enhances the magnitude of S34F-associated splicing. The “waterfall” plot depicts changes in percent inclusion levels for all shared cassette exons identified from the Venn diagram when the wild-type *U2AF1* locus was disrupted. Each vertical bar represents one shared cassette exon. **(B)**. The analysis shown in Panel A was repeated for cassette exons showing ten percent or more decreased inclusion in MUT1a cells. **(C)**. Heat map depicting the inclusion levels of all cassette exons that showed at least a 10% change in use among the treatment conditions. The dendrogram was constructed from an unsupervised cluster analysis. (**D**). Enhanced features of S34F-associated logos at 3′ splice acceptor sites after disruption of wild-type *U2AF1*. Sequence logos were constructed as in Fig 1A, based on the indicated comparisons.

Results in the preceding sections are based on studies of a few well-documented S34F-responsive cassette exons that likely serve as surrogates for the global effects of U2AF1S34F on splice site recognition. To determine whether these results reflect general rules governing S34F-associated splicing, we used RNA-seq to measure the consequences of altering S34F:WT ratios on global recognition of cassette exons. When wild-type *U2AF1* was diminished by CRISPR-Cas9-mediated disruption in MUT1a cells (see Fig 3C), the S34F-associated changes in inclusion (Fig 4A) or exclusion (Fig 4B) of cassette exons were enhanced. These global effects are consistent with our measurements of individual cassette exons by RT-qPCR (Fig 3C). An unsupervised cluster analysis suggests that disruption of wild-type *U2AF1* in MUT1a cells primarily enhances the magnitude of changes for S34F-associated cassette exons (Fig 4C). We also observed a modest increase in the preference for C versus T at the -3 position of the consensus 3′ splice sites of cassette exons that were promoted versus repressed in association with *U2AF1*S34F (Fig 4D) following reduction of wild-type *U2AF1* levels, consistent with the observed association between the S34F:WT mRNA ratio and typical S34F-associated consensus 3′ splice sites identified in LUAD tumor transcriptomes (Fig 1).

We observed similar results when we extended our analyses to include other types of alternative splicing beyond cassette exon recognition (S10 Fig). We used RT-qPCR to validate five splicing alterations that are sensitive to ablation of wild-type *U2AF1* in the presence of *U2AF1*S34F (S11 Fig). Two of the five events involve incorporation of cassette exons (in *ATR* and *MED15*), two involve competition between different 3′ splice sites (in *CASP8* and *SRP19*), and one involves a choice between two mutually exclusive exons (in *H2AFY*). We conclude that the importance of the S34F:WT ratio for S34F-dependent splicing changes extends from cassette exon recognition to other types of alternative splicing.

### Changes in RNA splicing correlated with relative binding affinities of mutant and WT U2AF1 complexes

Based on the role of U2AF1 in 3′ splice site recognition, we hypothesized that differential RNA binding by mutant and WT U2AF1 could contribute to the observed dependence of S34F-associated splicing on the WT:S34F ratios. It has previously been shown that the S34F mutation reduces the binding affinity of the U2AF1-containing complex for a representative skipped splice site [18]. However, whether changes in RNA binding could account for exon inclusion, as well as the generality of this observation, were unknown. We therefore tested whether altered RNA-binding affinity could explain mutation-dependent increases (*ZFAND1*, *FXR1*, *ATR*, *MED15*) and decrease (*CEP164*) in exon inclusion, as well as competing 3′ splice site recognition (*FMR1*). These splicing events exhibited S34F-associated alterations in both our isogenic cell lines (S1 Table) and in LUAD transcriptomes (S2 Table).

**Fig 5:**
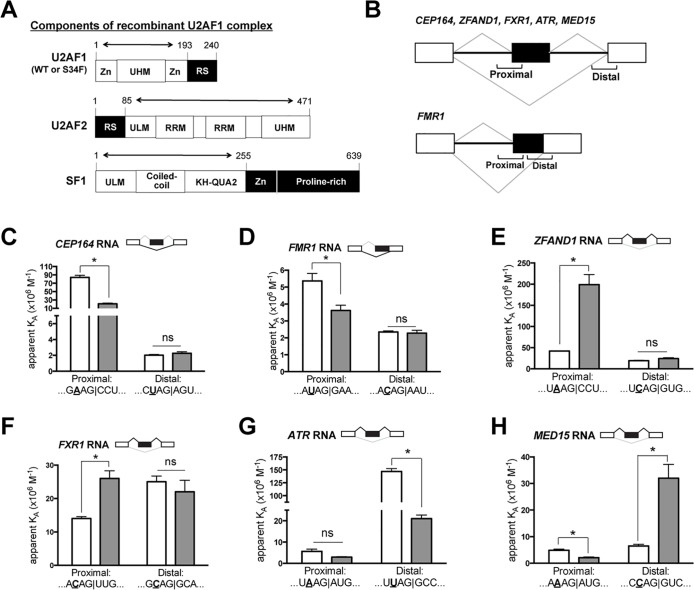
Differential binding of mutant and wild-type U2AF1 complexes to RNA oligonucleotides explains most S34F-associated alterations in RNA splicing. **(A)**. Cartoons illustrate components of recombinant U2AF1 complexes used in the binding assay. Full-length proteins are shown but only partial sequences (denoted by bi-directional arrows) were used to make recombinant protein complexes. KH-QUA2, K-Homology Quaking motif; RRM, RNA recognition motif domain; RS, arginine/serine-rich domain; UHM, U2AF homology motif; ULM, U2AF ligand motif; ZnF, zinc finger domain. **(B)**. Scheme of alternative splicing patterns for cassette exons (top diagram) and 5' extended exons from competing 3′ splice site selection (bottom). The brackets indicate the positions of RNA oligonucleotides used for the binding assays. Exons are shown as boxes: white boxes indicate invariant exonic sequences and black boxes denote sequences that are incorporated into mRNA (exonic) only when the proximal 3′ splice sites are used. Introns are shown as solid lines. The grey lines represent possible splices. The names of characterized genes that conform to the patterns shown in the upper and lower cartoons are indicated. **(C—H)**. Mutant and wild-type U2AF1 complexes have different affinities (K_A_’s) for relevant 3′ splice site oligonucleotides. To accomplish the binding assays, the wild-type or mutant U2AF1 protein complexes were titrated into 5' fluorescein-tagged RNA oligonucleotides over a range of concentrations as described in the Supplemental Materials and Methods. RNA sequences from -4 to +3 relative to the 3′ splice sites (vertical lines) in proximal and distal positions are shown. The nucleotide at the -3 position is bolded and underlined. Empty bars, K_A_ for WT U2AF1 complex; grey bar, K_A_ for mutant U2AF1 complex. For ease of comparison between the affinity binding results with S34F-associated splicing, S34F-promoted splices, as determined from RNA-seq data, are shown on top of each bar graph in black lines. The fitted binding curves, full oligonucleotide sequences, and apparent equilibrium dissociation constants are shown in S12 Fig. The relative changes in affinity and use of proximal *versus* distal splice sites are summarized in S3 Table. The asterisk represents a statistically significant change by unpaired t-tests with Welch’s correction. ns, not statistically significant.

We determined the RNA binding affinities of purified U2AF1-containing protein complexes using fluorescence anisotropy assays, in which the anisotropy increases of fluorescein-labeled RNA oligonucleotides following protein titration were fit to obtain the apparent equilibrium binding affinities (S12 Fig; Supplemental Materials and Methods). The recombinant proteins comprised either WT or S34F-mutant U2AF1 as ternary complexes with the U2AF2 and SF1 subunits that recognize the adjoining 3′ splice site consensus sequences. The constructs were nearly full length and included the relevant domains for 3′ splice site recognition [15,25,26] (Fig 5A). The six pairs of tested RNA oligonucleotides (33–35 nucleotides in length) were derived from the proximal or distal 3′ splice sites of the six genes listed above (Figs 5B and S12). Combined with prior results for sequence variants derived from the S34F-skipped *DEK* cassette exon [18], we have in total measured binding affinities for 16 RNA oligonucleotides, which consist of five sequences with a U at the -3 position of the 3′ splice site (“UAG” splice sites), seven “CAG” splice sites, and four “AAG” splice sites.

The trends among S34F-altered RNA binding affinities of U2AF1 complexes (Fig 5C–5H) for the tested splice site sequences generally agreed with the nucleotide distributions observed in consensus 3′ splice site that are promoted or repressed by *U2AF1*S34F (Figs 1A and S1A). The S34F mutation reduced the affinities of U2AF1-containing complexes for four out of five tested “UAG” splice sites, consistent with T as the most common nucleotide at the -3 position of the 3′ splice site (henceforth referred to as -3T) for S34F-repressed exons. Likewise, the S34F mutation often increased the affinities of U2AF1 complexes for “CAG” splice sites (for three out of seven tested sequences), consistent with -3C as the most common nucleotide preceding S34F-promoted exons. The remaining tested “UAG” or “CAG” splice sites showed no significant difference between S34F and WT protein binding. The “AAG” splice sites lacked a consistent relationship to the S34F-induced RNA affinity changes of U2AF1 complexes *per se*. However, the S34F mutation increased the binding of the U2AF1-containing complex for one “AAG” splice site for an S34F-promoted exon (*ZFAND1*), which is consistent with the greater prevalence of -3A than -3T preceding S34F-promoted exons.

Overall, the altered binding affinities of U2AF1-containing complexes for the proximal 3′ splice site could account for four of the six S34F-associated alternative splicing events (*CEP164*, *FMR1*, *ZFAND1*, *FXR1*) (Fig 5C–5F, Supplementary S3 Table). Similar to the previously-tested S34F-skipped splice site in *DEK* [18], the S34F mutation decreased affinities of the U2AF1-containing complexes for the skipped 3′ splice sites of the *CEP164* and *FMR1* exons (Fig 5C and 5D). Remarkably, the S34F mutation enhanced binding of the U2AF1-containing complexes to the proximal 3′ splice sites of *ZFAND1* and *FXR1* (Fig 5E and 5F), which could explain the enhanced inclusion of these exons in cell lines and LUAD (Supplementary S3 Table). In agreement with the observed splicing changes, the S34F mutation had no significant effect on the distal 3′ splice sites of these exons.

The affinities of the mutant U2AF1 complexes for the proximal splice site oligonucleotides of the remaining two S34F-promoted exons (*ATR* and *MED15*) were either similar to wild-type or decreased (Fig 5G and 5H). These results differed from the S34F-dependent increase in U2AF1 binding to the proximal 3′ splice site that was observed for *ZFAND1* and *FXR1* (Fig 5E and 5F), which could readily explain the enhanced exon inclusions. However, for the *ATR* pre-mRNA, the *U2AF1* mutation reduced binding to the distal more than to the proximal 3′ splice site (Fig 5G, third and fourth columns). Given co-transcriptional splicing in the 5'-to-3′ direction, the downstream (as opposed to upstream) 3′ splice sites could compete as splicing acceptors for a given 5' donor splice site when transcription is relatively rapid as compared to splicing. (Such 3′ splice site competition likely occurs relatively frequently, as the *ATR* cassette exon is alternatively spliced even in wild-type cells). As such, a “net gain” in affinity for the proximal relative to distal 3′ splice site could explain the observed S34F-associated exon inclusion in *ATR* mRNAs. For the *MED15* pre-mRNA, deviation of the S34F-associated splicing changes and a simple RNA affinity model suggested that additional mechanisms control selection of the *MED15* 3′ splice sites. In total, our measurements of *in vitro* RNA-binding affinities are sufficient to explain six of seven tested alterations in splice site recognition driven by *U2AF1*S34F (Fig 5C–5H and in [18]).

### HBEC3kt and LUAD cells were not dependent on *U2AF1*S34F for growth, but wild-type *U2AF1* was absolutely required

Other than its effect on RNA splicing, the consequences of the *U2AF1*S34F mutation on cell behavior are largely unknown. Recurrent mutations, such as *U2AF1 S34F*, are considered likely to confer a selective advantage to cells in which they occur when expressed at physiologically relevant levels. However, mutant HBEC3kt cells (MUT1a and MUT1b) do not exhibit obvious phenotypic properties of neoplastic transformation—such as a growth advantage over wild-type cells (S13 Fig) or an ability to grow in an anchorage-independent manner—that are frequently observed in cultured cells expressing well-known oncogenes, like mutant *RAS* genes.

Another attribute of some well-known oncogenes, such as *BCR-ABL* fusion in chronic myeloid leukemia or mutant *EGFR* or *KRAS* in LUAD, is the dependence on sustained expression of those oncogenes for the maintenance of cell growth or viability. To determine whether LUAD cells harboring a pre-existing S34F mutation are dependent upon (or “addicted to”) the mutant allele, we searched the COSMIC database for LUAD cell lines with the *U2AF1*S34F allele [27]. Two cell lines (H441 and HCC78) were found and both these cells exhibit copy number gains at the *U2AF1* locus (three copies for H441 cells; four copies for HCC78 cells) and one copy of a variant allele. We confirmed that *U2AF1*S34F was the minor allele in these cells by Sanger sequencing and allele-specific RT-qPCR (S14 Fig, panels A and B). We further used the CRISPR-Cas9 system to selectively disrupt the wild-type or mutant *U2AF1* sequences and then assessed the impact of inactivating the *U2AF1* alleles on the clonogenic growth of the two LUAD lines with the *U2AF1* mutation. In addition, we performed similar experiments with the LUAD cell line A549 (wild-type for U2AF1) and the HBEC3kt-derived MUT1a cell line.

**Fig 6:**
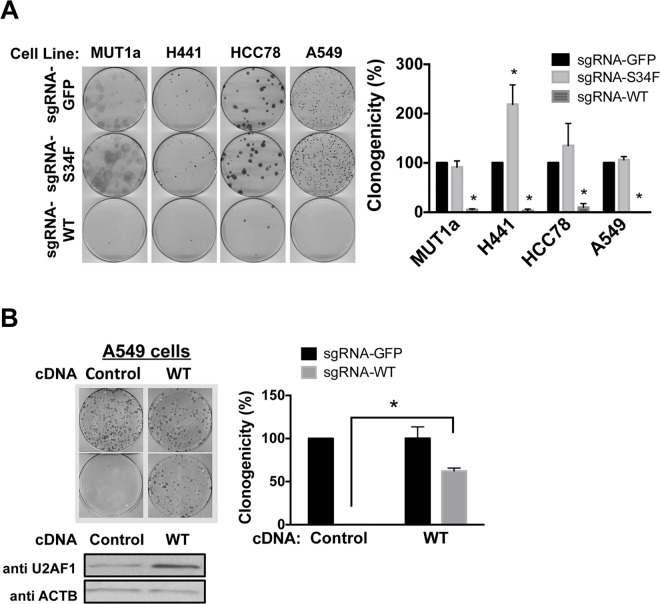
Wild-type but not mutant *U2AF1* is required for the clonogenic growth of the isogenic HBEC3kt cells and LUAD cell lines. **(A)**. Clonogenic growth assays after selective disruption of the wild-type or mutant *U2AF1* allele. Left panel: The indicated cell lines were transduced with lentiviruses expressing Cas9 and sgRNA-GFP, sgRNA-S34F or sgRNA-WT, followed by clonogenic assays. Cell colonies were stained with methylene blue and counted three weeks later. Right panel: Quantification of the clonogenic assay. The results are shown as percent clonogenicity by setting the number of control cell colonies (cells transduced with Cas9 and sgRNA-GFP) as 100%. Asterisks represent statistical significant changes as compared to Cas9 and sgRNA-GFP-transduced condition in each cell line. Error bars represent s.e.m (n = 3). **(B)**. Rescue of growth inhibition by Cas9 and sgRNA-WT by overexpressing a form of wild-type *U2AF1* cDNA that is not predicted to be the target for sgRNA-WT (See S8 Fig and S1 Text). Left panel: A549 cells were transduced with a control (DsRed-Express 2) or a wild-type *U2AF1* cDNA that is not predicted to be the target for sgRNA-WT. Increased expression of wild-type U2AF1 was confirmed by immunoblot (left bottom panel). These cells were subsequently transduced with Cas9 and either sgRNA-GFP or sgRNA-WT followed by clonogenic assays as in panel A (left upper panel). Right panel: Quantification as in Panel A. The asterisk represents a statistical significant change for the indicated comparison. Error bars represent s.e.m. (n = 3).

In all instances, loss of the mutant allele did not impair cell growth. Only one line (H441) exhibited altered growth, in the form of a two-fold increase in clonogenicity (Fig 6A). Successful disruption of the *U2AF1*S3F allele was confirmed by restoration of a normal RNA splicing profile in subclonal cells derived from the clonogenic assay colonies (see S14–S17 Figs, S4 and S5 Tables, and text below). In contrast, loss of the wild-type allele completely inhibited clonogenic growth in all tested cell lines, regardless of whether the line carried the *U2AF1*S34F allele or not. A rescue experiment confirmed that loss of cell growth was due to loss of wild-type *U2AF1* expression. The loss of clonogenic capacity after disrupting endogenous *U2AF1* in A549 cells was prevented by first transducing them with a form of wild-type *U2AF1* cDNA (Fig 6B) that is not predicted to be a target for sgRNA-WT (S8 Fig). Overall, these findings indicate that wild-type *U2AF1* is required for the clonogenic growth of cells, including lung cancer cell lines, that the S34F mutant is unable to compensate for loss of the wild-type allele, and that LUAD cells with the S34F mutation are not dependent on the mutant allele for growth *in vitro*.

To examine the effect of *U2AF1*S34F on tumor growth *in vivo*, we derived H441 and HCC78 cells transduced with Cas9 and sgRNA-S34F or sgRNA-GFP as polyclonal pools or as clones (S14 and S15 Figs). The cell lines were verified to either carry or not carry the *U2AF1*S34F allele, and we confirmed that the Cas9 and sgRNA-S34F-transduced cells lost the S34F-associated splicing program (S16 and S17 Figs).

We inoculated these subclonal cell lines subcutaneously in nude mice and monitored xenograft tumor growth. The H441-derived cell lines, in which the *U2AF1*S34F allele was disrupted, were able to establish tumors *in vivo* at rates similar to those observed for tumor cells carrying the mutant allele (S6 Table). The HCC78-derived cell lines did not grow palpable tumors after xenografting within the observation period, so the requirement for *U2AF1*S34F *in vivo* could not be tested in that line. These experiments show that *U2AF1*S34F is dispensable for growth of these LUAD cell lines *in vivo*, a result consistent with the clonogenicity assays shown in Fig 6. We conclude that *U2AF1*S34F appears to be neither sufficient nor necessary for lung cell transformation in these assays. In contrast, wild-type *U2AF1* is required for cell viability, consistent with the retention of a wild-type allele in human cancers carrying common *U2AF1* mutations.

## Discussion

In this report, we have examined the mechanistic and phenotypic consequences of the common *U2AF1*S34F mutation. Our data demonstrate that the S34F:WT ratio controls the quantitative consequences of the *U2AF1* mutation for splice site recognition, and suggest that differential RNA-binding affinities of the mutant and wild-type protein result in preferential recognition of specific 3′ splice sites. Moreover, our finding that wild-type *U2AF1* is required for cell survival irrespective of the presence or absence of *U2AF1*S34F explains the genetic observation that tumors always retain an expressed copy of the wild-type allele. Finally, we expect that the genetic models of *U2AF1*S34F that we derived from immortalized lung epithelial cells, as well as cell lines derived from lung adenocarcinomas with the mutation, will prove useful for future functional studies of this common mutation.

### The S34F:WT ratio controls S34F-associated splicing

U2AF1S34F is known to induce specific splicing alterations, but it is not known how these changes are regulated. We show that the ratio of S34F:WT U2AF1 gene products is a critical determinant of the magnitude of S34F-associated splicing. This conclusion was demonstrated in an isogenic lung epithelial cell line engineered to express *U2AF1*S34F from one of the two endogenous *U2AF1* loci, and was further supported by analyses of human LUAD transcriptomes carrying the *U2AF1*S34F allele. These results suggest that wild-type U2AF1 antagonizes the splicing program associated with the S34F mutation.

### Altered RNA binding affinities often explain S34F-associated splicing changes

We find that a major functional difference between purified S34F mutant and wild-type U2AF1 proteins resides in altered binding affinities for a subset of 3′ splice sites. The trends in the RNA sequence preferences of S34F-mutant U2AF1 are consistent with the preferred 3′ splice sites of S34F-affected transcripts (here and in [17–20,28]), which we use as the signature of S34F-associated differential splicing (Fig 1A). For oligonucleotides that showed significant changes in binding affinities, the S34F mutation typically reduced or enhanced respective binding of the U2AF1 splicing factor complexes to 3′ splice sites preceded by a -3U or -3C (Fig 5 and [18]). In support of our findings for the relevant ternary complex of human U2AF1, U2AF2 and SF1 subunits, recent studies confirmed that the corresponding S34F mutation inhibited binding of the minimal fission yeast U2AF heterodimer to a “UAG” splice site RNA [21]. These S34F-altered RNA affinities are consistent with the location of the substituted amino acid in a zinc knuckle that may directly contact RNA [17]. Although the effects of the S34F mutation on binding 3′ splice sites preceded by -3A are variable, extrusion or alternative U2AF1 binding sites for disfavored nucleotides could occur in different sequence contexts by analogy with other RNA binding proteins [29,30].

Several lines of evidence support the idea that U2AF1S34F is capable of initiating pre-mRNA splicing once it binds to RNA. Nuclear extracts of cells overexpressing mutant *U2AF1* can support *in vitro* splicing reactions more efficiently than nuclear extracts derived from cells overexpressing wild-type *U2AF1* for a minigene with a specific 3′ splice site sequence [17]. In addition, mutant U2AF1 can compensate for loss of the wild-type factor for the inclusion of some U2AF1-dependent cassette exons [31]. Our observations that the altered RNA-binding affinities correlate well with S34F-associated splicing for the majority of splice sites that we tested further suggest that mutant and wild-type U2AF1 are functionally equivalent for downstream steps of splicing (Fig 5 and [18]).

### A working model of S34F-associated splicing

In light of our findings and existing evidence from the literature, we propose a model wherein mutant U2AF1 drives differential splicing by favoring the recognition of one of two competing 3′ splice sites (S18 Fig). This model is motivated by three key facts. First, alternative splicing, in contrast to constitutive splicing, necessarily results from implicit or explicit competition between splice sites. (For example, cassette exon recognition can involve competition between the 3′ splice sites of the cassette exon itself and a downstream constitutive exon.) Second, mutant and wild-type U2AF1 complexes have different binding specificities, largely due to their preferences for distinct nucleotides at the -3 position, that lead them to preferentially bind distinct 3′ splice sites. Third, mutant and wild-type U2AF1 are likely functionally equivalent once they bind to RNA. Therefore, altering the cellular ratio of mutant and wild-type U2AF1 changes the amount of total U2AF1 bound to a given 3′ splice site in a sequence-specific manner, thereby promoting or repressing that splice site relative to a competing 3′ splice site.

Our proposed model is not exclusive of other possible contributing effects, such as competitive binding of mutant and wild-type U2AF1 to a factor with low stoichiometry (S18 Fig), or effects of U2AF1S34F on the kinetics of co-transcriptional splicing as suggested recently [32]. Future studies are needed to resolve these possibilities.

### How are cells with the U2AF1 S34F mutation selected during oncogenesis?

*U2AF1*S34F is recurrently found in LUAD, other solid tumors, and myeloid disorders, suggesting that the mutant allele confers a physiological property that provides a selective advantage during neoplasia. A gene ontology (GO) analysis for genes that show S34F-associated alterative splicing in HBEC3kt-derived isogenic cells shows significant alterations in many biological processes such as mRNA processing, RNA splicing, G2/M transition of mitotic cell cycle, double-strand break repair and organelle assembly (FDR-adjusted p-values < 0.001). However, we did not observe signs of neoplastic transformation or changes in cell proliferation after introducing *U2AF1*S34F into the endogenous *U2AF1* locus in HBEC3kt cells (S13 Fig). Moreover, targeted inactivation of *U2AF1*S34F in LUAD cell lines does not diminish, and in one case even increases, clonogenic growth in culture (Fig 6).

The lack of a testable cellular phenotype has been a major hindrance to understanding the functional significance of mutant *U2AF1* in carcinogenesis. Cell proliferation is only one of the many hallmarks of cancer, so careful examination of other cell properties in the isogenic cells may be needed to establish the presumptive role of *U2AF1*S34F in carcinogenesis.

More recently, after completion of our study [33], Park *et al* reported that tumorigenic cells emerge after *U2AF1*S34F is ectopically produced in Ba/F3 pro-B cells or in an immortalized line of small airway epithelial cells [34]. The authors attributed the transformation events by mutant U2AF1 to the consequences of altered 3′ processing of mRNA’s. In particular, they observed an increase in the length of the 3′ untranslated region of *ATG7* mRNA and a reduction in the amount of ATG7 protein, proposing that the anticipated defect in autophagy predisposes cells to mutations, some of which are transforming. This “hit-and-run” type of mechanism is consistent with our observation and theirs that mutant U2AF1 is dispensable for maintenance of the transformed phenotype in LUAD cell lines (Fig 6) and in their cell lines [34]. Their observations do not, moreover, exclude a role for S34F-associated splicing in the oncogenic mechanism.

### Can cancer cells carrying the S34F mutation be targeted therapeutically?

We have shown that the wild-type *U2AF1* allele is absolutely required for the growth of lung epithelial and LUAD cells that carry a mutant *U2AF1* allele (Fig 6). This result indicates that mutant U2AF1 cannot complement a deficiency of wild-type U2AF1; it may also explain why tumors homozygous for the *U2AF1*S34F mutation have not been observed, although the number of tumors found to have even a heterozygous mutation is still relatively small, so the analysis may not be adequately powered. Still, the frequent occurrence of a low ratio of S34F:WT *U2AF1* mRNA, accompanied by increased copy number of wild-type *U2AF1* alleles in lung adenocarcinoma cell lines and possibly LUADs, suggests that there may be selection for a lower ratio of S34F:WT in addition to the likely selection, perhaps at an earlier stage of tumorigenesis, for the S34F mutant.

These results are consistent with a study of mutations affecting the splicing factor SF3B1, which reported that cancer cells harboring recurrent *SF3B1* mutations also depend on wild-type SF3B1 for growth [35]. Finally, a recent study similarly found that a wild-type copy of *SRSF2* is required for leukemic cell survival, and that *SRSF2* mutations generated a therapeutic index for treatment with a small molecule that inhibits 3′ splice site recognition [36]. Together, our results and these recent studies provide a genetic rationale for targeting wild-type splicing factors (or the splicing machinery more generally) in cancers harboring spliceosomal mutations.

## Materials and Methods

### Ethics statement

The ethics statement is available in S1 Text.

### Cell culture, reagents, and assays

The HBEC3kt cells (a gift from Dr. John Minna, UT Southwestern Medical Center), H441, A549 (ATCC) and HCC78 cells (DMSZ) were cultured as previously described [22] or according to vendors’ instructions. The primary antibodies for immunoblots are: rabbit anti U2AF1 (1:5000, # NBP1-19121, Novus), rabbit anti GFP (1:5000, #A-11122, Invitrogen), mouse anti ACTB (1:5000, Clone 8H10D10, Cell Signaling). Lentiviruses were produced and titered in HEK293T cells as previously described [37]. An MOI (multiplicity of infection) of 1–5 were used for all assays.

Total RNA was extracted and reverse transcribed as previously described [38]. Splicing alterations were measured by quantitative PCR on reverse-transcribed cDNA (RT-qPCR), using isoform-specific primers (S7 Table). These primers were designed following a previously described method [39]. The PCR efficiency and specificity of each primer set were determined before they were used for measuring splicing changes (See Supplemental Materials and Methods for details).

Clonogenic assay was performed by infecting cells with lentiviruses two days before seeding them into 100 mm dishes (1000 live cells per dish) to grow colonies. Growth media were supplemented with puromycin (1 μlg/ml) for selecting infected cells and were changed once a week for up to three weeks. Cell colonies were stained with 0.03% methylene blue (in 20% methanol) for 5 min. Clonogenicity is defined as colony numbers formed as a percent of those in control cells.

Details of all DNA constructs used in the study and the genome editing approaches for creating the *U2AF1*S34F allele and allelic-specific disruption of *U2AF1* are described in the Supplemental Materials and Methods.

### mRNA sequencing and analysis

High throughput mRNA sequencing (RNA-seq) was conducted in the Sequencing Facility of the National Cancer Institute. RNA quality was assessed by 2100 Bioanalyzer (Agilent). Total RNA with good integrity values (RIN > 9.0) was used for poly A selection and library preparation using the Illumina TruSeq RNA library prep kit. Two or four samples were pooled per lane and ran on the HiSeq2000 or HiSeq2500 instrument using TruSeq V3 chemistry. All samples were sequenced to the depth of 100 million pair-end 101 bp reads per sample.

Splicing analysis of RNA-seq data from the TCGA LUAD cohort as well as engineered HBEC3kt, H441, and HCC78 cell lines was performed as previously described [17]. A brief description of the method was provided in the Supplemental Materials and Methods.

### Purification of U2AF1 protein complexes and RNA affinity measurement

Purification of U2AF1 complexes, as illustrated in Fig 5, was explained in Supplemental Materials and Methods. Sequences of synthetic 5′-labeled fluorescein RNAs (GE Healthcare Dharmacon) and binding curves are given in the Supplementary S12 Fig. Apparent equilibrium affinity constant of the purified U2AF1 complexes with RNA was measured based on changes in fluorescence anisotropy as previously described [18]. A brief description of this method was provided in the Supplemental Materials and Methods.

### Statistics

All experiments were independently performed at least three times unless otherwise stated. Statistical significance was determined by two-tailed Student's *t* test or otherwise stated. In all analyses, *p* values ≤ 0.05 are considered statistically significant.

## Supporting Information

S1 Text Supplemental Materials and Methods (DOCX)

S1 Fig Sequence logos from 3′ splice sites reveal the influence of *U2AF*S34F on some but not all reported LUAD transcriptomes. As in Fig 1A, sequence logos of the region flanking 3′ splice sites were computed from LUAD transcriptomes reported by TCGA. Panel **A**: Logos from tumors carrying a *U2AF1*S34F mutation and showing “typical S34F” consensus 3′ splice sites. Panel **B**: Logos from tumors carrying the *U2AF1*S34F mutation but with compromised S34F-associated features at the -3 position (“quasi-WT” pattern). Panel **C**: Logos from tumors without a *U2AF1* mutation. Sequence logos deduced from tumor transcriptomes #7903 and #7727 are also displayed in Fig 1A.
(PDF)

S2 Fig Characteristics of LUAD carrying a *U2AF1*S34F mutation relevant to splicing patterns. Tumors with transcriptomes that display “typical S34F” or “quasi-WT” consensus 3′ splice sites (Figs 1A, S1A and S1B) are plotted to compare with the level of *U2AF1*S34F mRNA (panel **A**), the level of total *U2AF1* mRNA (panel **B**), or the percentage of tumor nuclei in the tissue samples (panel **C**). The definitions of the shape and color of each dot are the same as in Fig 1, panels **B**—**D**. (**D**), The amount of *U2AF1* mRNA is displayed in relation to gains and losses of *U2AF1* DNA copy number. Box indicates *U2AF1* mRNA levels from the 25th to 75th percentiles within the group. The median value is represented as a line in box. Error bars represent the range of values. TPM, transcripts per million. ns, not significant as determined by student’s t test (p value > 0.05).
(PDF)

S3 Fig S34F:WT mRNA ratios correlate with exon inclusion or repression of three representative cassette exons. Pearson’s correlation was calculated to seek relationships between the inclusion levels of five representative splicing events and four factors in several LUAD tumors in the TCGA data set: the S34F:WT mRNA ratio, the level of *U2AF1*S34F mRNA, total *U2AF1* mRNA, or percent tumor nuclei. Panels **A**–**D**, cassette exon in *STRAP* mRNA that is preferentially skipped in the presence of the *U2AF1*S34F mutation. Panels **E**–**H**, cassette exon in *ASUN* mRNA that is preferentially skipped in the presence of the *U2AF1 S34F* mutation; Panels **I**–**L**, cassette exon in *ATR* mRNA that is preferentially included in the presence of the *U2AF1*S34F mutation; Panels **M**–**P**, the 5' extension of the *FMR1* exon that is less frequently included in the presence of the *U2AF1*S34F mutation; Panel **Q**–**T**, the 5' extension of the *CASP8* exon that is less frequently included in the presence of the *U2AF1*S34F mutation. *U2AF1*-mutant LUAD transcriptomes containing sufficient number of informative reads to calculate the inclusion frequency were included in the analysis. The median usage of the indicated isoform for all transcriptomes from tumors without the mutation is shown as a triangle. The definitions of the shape and color of each dot are the same as in Fig 1, panels **B**—**D**. r, Pearson’s association coefficient. Panel **A** is also shown in Fig 1D. TPM, transcripts per million.
(PDF)

S4 Fig Characterization of intermediate and final cell clones for generating isogenic mutant and wild-type HBEC3kt cell lines. **(A).** Strategy for identifying intermediate cell clones by Southern blot. Left Panel: Southern blot probes and the expected fragment sizes after restriction enzyme digestion of genomic DNA. Right Panel: Details of the Southern blot strategy. E, EcoR I; X, Xho I; E1, E2, E8, exons 1, 2, and 8 (black boxes); E2/mE2, wild-type or S34F-mutant exon 2; yellow box, drug selection cassette (PGK-Hygro∆TK) flanked by inverted terminal repeat sequences for recognition by the Piggybac transposase (green lines); thick black lines, Southern blot probes. **(B).** Drug-resistant intermediate clones were identified by Southern blot. Cell clones with the expected pattern of restriction fragments are marked with a star. **(C)**. Top panel: Strategy for identifying mutant and wild-type intermediate clones by PCR amplicon sequencing. The PCR primer P1-F is upstream of the 5' homology arm sequence from the donor vector, while P1-R is within the drug selection cassette. Therefore, the PCR amplicon is specific for the intermediate allele. Bottom Panel: Sanger sequencing results show that one of the three intermediate clones, #21, carried the S34F mutation, while clones #12 and #16 were wild-type for *U2AF1*. The codons (on the reverse strand) for Ser34 or Phe34 are underlined. **(D)**. The relationship of the intermediate and final cell clones. **(E)**. U2AF1 mRNA and protein levels were similar in the final cell clones. Top Panel: *U2AF1* mRNA was measured by RT-qPCR and normalized to levels of *GAPDH* mRNA. The relative *U2AF1* mRNA level in WT1 cells was set to 1.0. (Bottom) Immunoblots for U2AF1 and ACTB using total cell lysates from the indicated cell lines.
(PDF)

S5 Fig The allele-sensitive S34F/WT Single Nucleotide Polymorphism (SNP) Taqman assay measures the ratio of S34F:WT DNA and mRNA quantitatively. **(A).** Upper Panel: Sequences of the primers and probes used in the allele-sensitive S34F/WT SNP Taqman assay. The nucleotide corresponding to the S34F missense mutation (reverse strand) is underlined. Bottom Panel: Diagram to show that all the primers (arrows) and probes (bar) are located within exon 2, allowing detection of the S34F:WT *U2AF1* ratio for both genomic DNA and mRNA. **(B).** The S34F and wild-type (WT) probes are specific for their targets. Characterization of probe specificity was performed using plasmid DNA carrying either the WT or S34F mutant exon 2 of *U2AF1*. Eight 10-fold serial dilutions of the plasmid DNA templates were used (starting concentration: 2 x 10^8 molecules per 10 μl reaction), and the assay was performed in triplicate. According to the qPCR amplification curves (delta Rn vs. cycle), the WT probe is specific for wild-type *U2AF1*, while the S34F probe can detect wild-type *U2AF1* at a lower efficiency (a mean difference of 3.5 cycles (equivalent to 11-fold) for DNA templates at the same concentration). **(C)**. The mRNA levels of S34F and wild-type *U2AF1* are similar in MUT1a and MUT1b cells, as revealed by the allele-sensitive S34F/WT SNP Taqman Assay (the S34F:WT mRNA ratio approximated 1). This result is consistent with the RNA-seq result in Fig 2B.
(PDF)

S6 Fig Alternative usage of cassette exons associated with U2AF1 S34F-mutant cancers is confirmed in isogenic HBEC cell lines. The relative amounts of short and long isoforms of the designated genes were measured by RT-qPCR to estimate changes in the inclusion of cassette exons, that were previously reported to be affected by the *U2AF1*S34F mutation as judged by RNA-seq of LUAD and AML tumor samples from TCGA [19]. The splicing alterations of the *ASUN* and *STRAP* cassette exons are also shown in Fig 2C. Asterisks represent statistically significant changes compared to WT1 cells. Error bars represent s.e.m. (n = 4).
(PDF)

S7 Fig Knockdown of total *U2AF1* mRNA in MUT1a cells does not affect S34F-associated splicing. Additional assays used in the studies shown in Fig 3A were performed on WT1 and MUT1a cell lines. **(A)**. Knockdown of total *U2AF1* mRNA with the indicated shRNAs does not affect S34F:WT mRNA ratios in MUT1a cells, as measured by the allele-sensitive Taqman S34F/WT SNP assay. **(B)**. The indicated *U2AF1* shRNAs reduced total *U2AF1* mRNA levels. **(C, D).** Reduction of *U2AF1* mRNA does not affect the inclusion of cassette exons in *USP25* and *AXL* mRNAs. Both of the cassette exons showed increased inclusion in the presence of *U2AF1*S34F (S1 Table). **(E)**. Reduction of total *U2AF1* mRNA favors the selection of the isoform without the 5' extended exon (“short” isoform) in a competing 3′ splice site event in *SLC25C2* mRNA in both WT1 and MUT1a cells. Selection of the isoform containing the 5' extended exon (“long” isoform) was shown to be dependent on U2AF1 in cells expressing only wild-type U2AF1 [24]. The cartoon above each panel depicts the type of alternative splicing being measured. Asterisks represent statistically significant changes compared to shScbr-transduced conditions in respective cell lines. Error bars represent s.e.m (n = 3).
(PDF)

S8 Fig Sequence alignment of sgRNA-WT and sgRNA-S34F with human and mouse *U2AF1* DNA sequences. DNA sequences encoding sgRNA-WT and sgRNA-S34F are identical to human *U2AF1* genomic DNA except at the middle position of the S34 codon (underlined) in the mutant sequence. The CGG sequence (in red) served as the protospacer adjacent motif (PAM) for CRISPR-Cas9. The corresponding mouse *U2af1* genomic sequence, which was used to construct the *U2AF1* cDNA for overexpression (Fig 3B) and rescue assays (Fig 6B), is also shown. The two differences between the mouse and human sequences are marked in grey in the mouse sequence.
(PDF)

S9 Fig sgRNA-WT and sgRNA-S34F preferentially target the WT and mutant *U2AF1* alleles. MUT1a cells were transduced with lentiviruses expressing Cas9 and either sgRNA-GFP, sgRNA-WT, or sgRNA-S34F. Cells were harvested 6 days later for (panel **A**) quantifying the S34F:WT mRNA ratios by the allele-sensitive S34F/WT SNP Taqman assay or (panel **B**) RNAseq for counting the number of reads supporting either wild-type or mutant *U2AF1*. Error bars represent standard deviation (s.d.) from a representative experiment in panel **A**.
(PDF)

S10 Fig Increasing the ratio of S34F:WT gene products increases S34F-associated splicing at sites other than cassette exons. The analysis of experiments shown in Fig 4, Panels A and B, was extended to several different types of alterative splicing events including competing 5' splice sites, competing 3′ splice sites, retained introns and other types of alternative splicing described in S1 Table. Events with increased or decreased PSI (Percent Spliced In) are shown separately in panel **A** or **B**. Cassette exon events (displayed alone in Fig 4, panels A and B) are not included in this analysis.
(PDF)

S11 Fig S34F-associated alternative splicing is enhanced by disrupting the wild-type *U2AF1* locus to increase the ratio of S34F:WT gene products. Samples from the experiment shown in Fig 3C were used to measure the frequency of alternative splicing of selected transcripts (Panel **A**. *ATR*; Panel **B**. *MED15*; Panel **C**. *H2AFY*; Panel **D**. *CASP8*; Panel **E**. *SRP19*), using RT-qPCR with isoform-specific primers in a droplet digital PCR instrument. The cartoon above each panel depicts the type of alternative splicing being measured. The relative isoform ratio in control WT1 cells (transduced with sgRNA-WT and Cas9) is set to one. Student’s t test was used for comparing the difference in the indicated comparisons. Asterisks represent statistically significant changes. Error bar represents s.e.m. (n = 3).
(PDF)

S12 Fig Binding curves of the RNA binding assay in Fig 5. The wild-type or mutant (S34F) U2AF1 protein complexes with U2AF2 and SF1 were titrated into 5' fluorescein-tagged RNA oligonucleotides over the indicated range of concentrations. The average fluorescence anisotropy data points and error bars of three independent titrations are overlaid with the nonlinear fits as described in the Supplemental Materials and Methods. The RNA sequences are indicated below each graph title. The average values and standard deviations of the apparent equilibrium dissociation constants (K_D_) that resulted from three replicates are inset.
(PDF)

S13 Fig *U2AF1 S34F* does not affect cell proliferation or viability. (**A**, **B**). Equal numbers of isogenic HBEC cells (WT1, WT2, MUT1a and MUT1b) were plated and the numbers of live and dead cells were quantified over the course of three days. Cell viability was determined by Trypan Blue staining. (**C**). Cell viability was calculated as the ratio of dead versus live cells. Error bars represent s.d. from a representative experiment.
(PDF)

S14 Fig Two LUAD cell lines naturally carry the *U2AF1*S34F allele and are subject to disruption of the mutant allele by CRISPR-Cas9. **(A)**. *U2AF1*S34F is the minor allele in H441 and HCC78 lung adenocarcinoma cell lines. Genomic PCR was performed for H441 and HCC78 cells, using a PCR primer pair that flanks the codon Serine 34 (forward, GCAAGGAAGAGGAGGTGCTTA; reverse, AAGTCGATCACCTGCCTCAC). The PCR amplicon was subject to Sanger sequencing. A region containing the codon Serine 34 (in grey) is shown. “TCT” (encoding Serine) is the wild-type sequence, while “TTT” encodes Phenylalanine. (**B**). Polyclonal cell lines were established for H441, HCC78 and A549 cells transduced with Cas9 and either sgRNA-GFP or sgRNA-S34F, followed by RNA extraction and RT-qPCR to determine the ratio of mutant and wild-type *U2AF1* mRNA by the allele-sensitive S34F/WT SNP Taqman assay. **(C)**. The relative short/long isoform ratios of *ASUN* and *STRAP* mRNAs were measured as in Fig 1D. The isoform ratio in A549 cells transduced with Cas9 and sgRNA-GFP was set to 1. Asterisks represent statistically significant changes compared to the respective cell lines transduced with Cas9 and sgRNA-GFP. Error bars represent s.e.m. (n = 3).
(PDF)

S15 Fig Characterization of clonal cell lines derived from H441, HCC78, or isogenic HBEC cells after allelic-specific disruption of the *U2AF1* loci. (**A**). The S34F:WT DNA ratios was examined in clonal cell lines derived from H441 and HCC78 LUAD lines and MUT1a cells after transduction with Cas9 and sgRNA-GFP (boxed lines) or sgRNA-S34F. The clones were expanded and the genomic DNA was harvested to measure the ratio of mutant and WT *U2AF1* DNA by the allele-sensitive S34F/WT SNP Taqman assay. (**B**). Levels of U2AF1 and ACTB protein were measured by immunoblots in extracts from selected MUT1a-derived cell clones from Panel **A**, as well as from two clones derived from WT1 cells. The WT1-derived cell clones lack one intact WT *U2AF1* allele and were generated by transiently transfecting WT1 cells with Cas9 and sgRNA-GFP or sgRNA-WT. **(C)**. As in Fig 2C, the isoform ratios of *ASUN* and *STRAP* mRNAs were measured in MUT1a- or WT1-derived cell clones. Error bars represent s.d. from a representative experiment.
(PDF)

S16 Fig Comparison of isogenic U2AF1-mutant versus U2AF1-wild-type H441 LUAD cell lines show features of S34F-associated splicing. Whole transcriptome of selected H441 cell clones were measured by RNA-seq. The changes in cassette exon inclusion levels were compared as Fig 2, panels D and E. (**A**). Heat map depicting the inclusion levels of altered cassette exons. Dendrograms were constructed from an unsupervised cluster analysis based on all cassette exons that showed at least a 10% change in use between the indicated cell lines. **(B).** Sequence logos from 3′ splice sites preceding cassette exons with altered use in control H441 cells (a polyclone line transduced with Cas9 and sgRNA-GFP), comparing to H441 cells with the *U2AF1*S34F allele disrupted (a polyclone line transduced with Cas9 and sgRNA-S34F), display typical S34F-associated features. Logos were constructed as in Fig 1A. Comparisons of transcriptomes from the clonal lines (H441 cells sgRNA-GFP clone #1 vs. sgRNA-S34F clone #1) yield similar consensus 3′ splice site sequences.
(PDF)

S17 Fig Comparison of isogenic U2AF1-mutant versus U2AF1-wild-type HCC78 LUAD cell lines show features of S34F-associated splicing. Same as S16 Fig but selected HCC78-derived cell clones were used.
(PDF)

S18 Fig Working models of S34F-associated splicing program. **A**. Competition occurs between the proximal and distal 3′ splice sites in the presence of wild-type U2AF1 and its co-factors (yellow ellipse), resulting in alternative usage of the cassette exon (black box) under steady state. Mutant U2AF1 and its co-factors (red ellipse) have different affinities for the proximal and/or distal 3′ splice sites, altering the usage of the cassette exon. The brackets indicate regions surrounding the proximal and distal 3′ splice sites. **B**. In this alternative mode, wild-type U2AF1 binds to a co-factor (or co-factors, represented by a blue circle) that is presented at a lower stoichiometry. The wild-type complex recognizes the 3′ splice sites to mediate the recognition of the cassette exon. Mutant U2AF1 competes with wild-type U2AF1 for this rating-limiting co-factor. Mutant U2AF1-containing complex has different affinities for the splice sites, resulting in alteration in the inclusion of the cassette exon. In these cartoons, blank boxes represent constitutive exons flanking the cassette exon. Introns are the black lines connecting the exons. Arrows with solid or dotted lines represent affinities of the splicing factors to RNA. Grey lines represent possible splices and the weight of the lines represents altered splicing efficiency.
(PDF)

S1 Table Differentially spliced events in HBEC3kt derivatives with or without a *U2AF1 S34F* mutation. Each row of the table corresponds to an isoform of a splicing event that is differentially spliced in at least one of the indicated comparisons between cell lines that do or do not express *U2AF1*S34F. In each case, the isoform for which information is given is as follows: the inclusion isoform for cassette exons (type “se”), the intron-proximal isoform for competing 5' or 3′ splice site events (types “a5ss” or “a3ss”), inclusion of the upstream exon for mutually exclusive exon events (type “mxe”), splicing of retained introns annotated as alternative (type “ri”) or constitutive (type “ci”), or canonical splicing of an annotated constitutive junction (type “cj”). The columns specify relevant annotations for each event as follows: “Coordinates” specifies the genomic coordinates of the event; “Gene” specifies the Ensembl gene ID; “Gene Name” specifies the gene name; “Gene Description” is a description of the gene as given by Ensembl; “Type” specifies the type of splicing event; “NMD Target” specifies whether the isoform is a predicted substrate for degradation by nonsense mediated decay (NMD) (NA indicates that either all or no isoforms of the event are predicted NMD substrates). The columns prefixed by “Psi_” indicate the inclusion frequency of the above-mentioned isoforms in a given sample. The columns prefixed by “deltaPsi_” give the absolute difference in isoform ratio (Percent Spliced In or “Psi” value) for the indicated comparisons. Table is restricted to events that are differentially spliced in at least one comparison.
(XLS)

S2 Table Differentially spliced events in LUAD samples with or without a U2AF1 S34F mutation. Same as S1 Table, but for splicing events that are differentially spliced in the thirteen LUAD samples with the *U2AF1*S34F mutation versus all WT samples by group comparison. “p-value”, p-value computed with the Mann-Whitney U test for group comparisons.
(XLS)

S3 Table Changes in affinity largely explain S34F-associated splicing. Summary of RNA binding affinity changes (Figs 5 and S12) in relationship with observed splicing alterations in isogenic HBEC3kt cells and LUAD samples (S1 and S2 Tables). The columns “Gene Name”, “Coordinates”, “Type” and those prefixed by “deltaPsi_” are defined the same way as those in S1 Table. The remaining columns of the table are defined as follows: “Proximal -3 base” and “Distal -3 base”, nucleotide base immediate upstream of the invariable AG of the corresponding splice site (See Fig 5B for the location of proximal and distal sites); “Proximal S34F vs. WT Ka Fold Change” and “Distal S34F vs. WT Ka Fold Change”, fold change in affinities of the mutant and wild-type U2AF1 complexes for the corresponding splice junction sequence; ns, no significant difference by two tailed t test; “Predicted Effect by U2AF1S34F”, increased or decreased inclusion of the cassette exon or the intron-proximal 5' end of an exon by mutant U2AF1 that is predicted based on fold changes in affinity for both proximal and distal splice site sequences.
(XLS)

S4 Table Differentially spliced events in H441 derivatives with or without a *U2AF1 S34F* mutation. Same as S1 Table, but for H441-derived cell clones.
(XLS)

S5 Table Differentially spliced events in HCC78 derivatives with or without a *U2AF1 S34F* mutation. Same as S1 Table, but for HCC78-derived cell clones.
(XLS)

S6 Table *U2AF1*S34F is not required for xenograft tumor growth. The indicated cell lines were derived after transducing them with Cas9 and sgRNA-GFP or sgRNA-S34F (see S14–S17 Figs for characterization). Equal number of cells was transplanted into both flanks of CD1-nude mice and tumor growth was monitored. In Experiment #1, 1 million cells were inoculated in each flank and tumor growth was monitored for up to 21 weeks (4 or 5 mice per group). In Experiment #2, 2 million cells (H441-derived lines) or 5 million cells (HCC78-derived lines) were inoculated in each flank and tumor growth was monitored for 12 weeks (4 mice per group). At the end of the experiment, an average tumor volume of 100 mm^3^ or more (per flank) was considered successful tumor engraftment. The numbers of mice with successful vs. unsuccessful tumor engraftment (Tumor / No Tumor) were shown.
(XLS)

S7 Table List of primer sequences used for examining splicing alterations. The columns “Gene Name”, “Coordinates” and “Type” are defined the same way as those in S1 Table. The “short” isoforms refer to those with skipping of cassette exon or selection of an intron-distal 3′ splice site. The “long” isoforms refer to those with increased inclusion of cassette exon or selection of an intron-proximal 3′ splice site.
(XLS)

## References

[1] Yoshida K, Sanada M, Shiraishi Y, Nowak D, Nagata Y, Yamamoto R, et al Frequent pathway mutations of splicing machinery in myelodysplasia. Nature. 2011;478: 64–69. 10.1038/nature10496 21909114

[2] Graubert TA, Shen D, Ding L, Okeyo-Owuor T, Lunn CL, Shao J, et al Recurrent mutations in the U2AF1 splicing factor in myelodysplastic syndromes. Nature Genetics. 2012;44: 53–57. 22158538 10.1038/ng.1031 PMC3247063

[3] Imielinski M, Berger AH, Hammerman PS, Hernandez B, Pugh TJ, Hodis E, et al Mapping the hallmarks of lung adenocarcinoma with massively parallel sequencing. Cell. 2012;150: 1107–1120. 10.1016/j.cell.2012.08.029 22980975 PMC3557932

[4] Furney SJ, Pedersen M, Gentien D, Dumont AG, Rapinat A, Desjardins L, et al SF3B1 Mutations Are Associated with Alternative Splicing in Uveal Melanoma. Cancer Discovery. 2013;3: 1122–1129. 10.1158/2159-8290.CD-13-0330 23861464 PMC5321577

[5] Wang L, Lawrence MS, Wan Y, Stojanov P, Sougnez C, Stevenson K, et al SF3B1and Other Novel Cancer Genes in Chronic Lymphocytic Leukemia. N Engl J Med. 2011;365: 2497–2506. 10.1056/NEJMoa1109016 22150006 PMC3685413

[6] Waterfall JJ, Arons E, Walker RL, Pineda M, Roth L, Killian JK, et al High prevalence of MAP2K1 mutations in variant and IGHV4-34-expressing hairy-cell leukemias. Nature Genetics. 2014;46: 8–10. 10.1038/ng.2828 24241536 PMC3905739

[7] Cancer Genome Atlas Network. Comprehensive molecular portraits of human breast tumours. Nature. 2012;490: 61–70. 10.1038/nature11412 23000897 PMC3465532

[8] Biankin AV, Waddell N, Kassahn KS, Gingras M-C, Muthuswamy LB, Johns AL, et al Pancreatic cancer genomes reveal aberrations in axon guidance pathway genes. Nature. 2012;491: 399–405. 10.1038/nature11547 23103869 PMC3530898

[9] Cancer Genome Atlas Research Network. Comprehensive molecular profiling of lung adenocarcinoma. Nature. 2014;511: 543–550. 10.1038/nature13385 25079552 PMC4231481

[10] Ruskin B, Zamore PD, Green MR. A factor, U2AF, is required for U2 snRNP binding and splicing complex assembly. Cell. 1988;52: 207–219. 2963698 10.1016/0092-8674(88)90509-0

[11] Zamore PD, Green MR. Identification, purification, and biochemical characterization of U2 small nuclear ribonucleoprotein auxiliary factor. Proc Natl Acad Sci U S A. 1989;86: 9243–9247. 2531895 10.1073/pnas.86.23.9243 PMC298470

[12] Krämer A, Utans U. Three protein factors (SF1, SF3 and U2AF) function in pre-splicing complex formation in addition to snRNPs. The EMBO Journal. 1991;10: 1503–1509. 1827409 10.1002/j.1460-2075.1991.tb07670.x PMC452814

[13] Berglund JA, Abovich N, Rosbash M. A cooperative interaction between U2AF65 and mBBP/SF1 facilitates branchpoint region recognition. Genes & Development. 1998;12: 858–867. 9512519 10.1101/gad.12.6.858 PMC316625

[14] Merendino L, Guth S, Bilbao D, Martínez C, Valcárcel J. Inhibition of msl-2 splicing by Sex-lethal reveals interaction between U2AF35 and the 3′ splice site AG. Nature. 1999;402: 838–841. 10.1038/45602 10617208

[15] Wu S, Romfo CM, Nilsen TW, Green MR. Functional recognition of the 3′ splice site AG by the splicing factor U2AF35. Nature. 1999;402: 832–835. 10.1038/45590 10617206

[16] Zorio DA, Blumenthal T. Both subunits of U2AF recognize the 3′ splice site in Caenorhabditis elegans. Nature. 1999;402: 835–838. 10.1038/45597 10617207

[17] Ilagan JO, Ramakrishnan A, Hayes B, Murphy ME, Zebari AS, Bradley P, et al U2AF1 mutations alter splice site recognition in hematological malignancies. Genome Res. 2015;25: 14–26. 10.1101/gr.181016.114 25267526 PMC4317169

[18] Okeyo-Owuor T, White BS, Chatrikhi R, Mohan DR, Kim S, Griffith M, et al U2AF1 mutations alter sequence specificity of pre-mRNA binding and splicing. Leukemia. 2015;29: 909–917. 10.1038/leu.2014.303 25311244 PMC4391984

[19] Brooks AN, Choi PS, de Waal L, Sharifnia T, Imielinski M, Saksena G, et al A Pan-Cancer Analysis of Transcriptome Changes Associated with Somatic Mutations in U2AF1 Reveals Commonly Altered Splicing Events. Ast G, editor. PLoS ONE. 2014;9: e87361 10.1371/journal.pone.0087361 24498085 PMC3909098

[20] Przychodzen B, Jerez A, Guinta K, Sekeres MA, Padgett R, Maciejewski JP, et al Patterns of missplicing due to somatic U2AF1 mutations in myeloid neoplasms. Blood. 2013;122: 999–1006. 10.1182/blood-2013-01-480970 23775717 PMC3739042

[21] Yoshida H, Park S-Y, Oda T, Akiyoshi T, Sato M, Shirouzu M, et al A novel 3′ splice site recognition by the two zinc fingers in the U2AF small subunit. Genes & Development. 2015;29: 1649–1660. 26215567 10.1101/gad.267104.115 PMC4536312

[22] Ramirez RD, Sheridan S, Girard L, Sato M, Kim Y, Pollack J, et al Immortalization of human bronchial epithelial cells in the absence of viral oncoproteins. Cancer Res. 2004;64: 9027–9034. 10.1158/0008-5472.CAN-04-3703 15604268

[23] Yusa K. Seamless genome editing in human pluripotent stem cells using custom endonuclease-based gene targeting and the piggyBac transposon. Nat Protoc. 2013;8: 2061–2078. 10.1038/nprot.2013.126 24071911

[24] Kralovicova J, Vorechovsky I. Allele-specific recognition of the 3′ splice site of INS intron 1. Hum Genet. 2010;128: 383–400. 10.1007/s00439-010-0860-1 20628762 PMC2939332

[25] Liu Z, Luyten I, Bottomley MJ, Messias AC, Houngninou-Molango S, Sprangers R, et al Structural basis for recognition of the intron branch site RNA by splicing factor 1. Science. 2001;294: 1098–1102. 10.1126/science.1064719 11691992

[26] Agrawal AA, Salsi E, Chatrikhi R, Henderson S, Jenkins JL, Green MR, et al An extended U2AF(65)-RNA-binding domain recognizes the 3′ splice site signal. Nat Commun. 2016;7: 10950 10.1038/ncomms10950 26952537 PMC4786784

[27] Forbes SA, Beare D, Gunasekaran P, Leung K, Bindal N, Boutselakis H, et al COSMIC: exploring the world's knowledge of somatic mutations in human cancer. Nucleic Acids Res. 2015;43: D805–11. 10.1093/nar/gku1075 25355519 PMC4383913

[28] Shirai CL, Ley JN, White BS, Kim S, Tibbitts J, Shao J, et al Mutant U2AF1 Expression Alters Hematopoiesis and Pre-mRNA Splicing In Vivo. Cancer Cell. Elsevier Inc; 2015;27: 631–643. 10.1016/j.ccell.2015.04.008 25965570 PMC4430854

[29] Lu G, Hall TMT. Alternate modes of cognate RNA recognition by human PUMILIO proteins. Structure. 2011;19: 361–367. 10.1016/j.str.2010.12.019 21397187 PMC3063405

[30] Miller MT, Higgin JJ, Hall TMT. Basis of altered RNA-binding specificity by PUF proteins revealed by crystal structures of yeast Puf4p. Nat Struct Mol Biol. 2008;15: 397–402. 10.1038/nsmb.1390 18327269 PMC2802072

[31] Shao C, Yang B, Wu T, Huang J, Tang P, Zhou Y, et al Mechanisms for U2AF to define 3′ splice sites and regulate alternative splicing in the human genome. Nat Struct Mol Biol. 2014;21: 997–1005. 10.1038/nsmb.2906 25326705 PMC4429597

[32] Coulon A, Ferguson ML, de Turris V, Palangat M, Chow CC, Larson DR. Kinetic competition during the transcription cycle results in stochastic RNA processing. Elife. 2014;3 25271374 10.7554/eLife.03939 PMC4210818

[33] Fei DL, Motowski H, Chatrikhi R, Prasad S, Yu J, Gao S, et al Wild-type U2AF1 antagonizes the splicing program characteristic of U2AF1-mutant tumors and is required for cell survival. Preprint. Available from bioRxiv. 2016 4 10.1101/048553 PMC5077151 27776121

[34] Park SM, Ou J, Chamberlain L, Simone TM, Yang H, Virbasius C-M, et al U2AF35(S34F) Promotes Transformation by Directing Aberrant ATG7 Pre-mRNA 3. Mol Cell. Elsevier Inc; 2016;: 1–34. 27184077 10.1016/j.molcel.2016.04.011 PMC5012111

[35] Zhou Q, Derti A, Ruddy D, Rakiec D, Kao I, Lira M, et al A Chemical Genetics Approach for the Functional Assessment of Novel Cancer Genes. Cancer Res. 2015;75: 1949–1958. 10.1158/0008-5472.CAN-14-2930 25788694

[36] Lee SC-W, Dvinge H, Kim E, Cho H, Micol J-B, Chung YR, et al Modulation of splicing catalysis for therapeutic targeting of leukemia with mutations in genes encoding spliceosomal proteins. Nat Med. 2016 27135740 10.1038/nm.4097 PMC4899191

[37] Fei DL, Sanchez-Mejias A, Wang Z, Flaveny C, Long J, Singh S, et al Hedgehog Signaling Regulates Bladder Cancer Growth and Tumorigenicity. Cancer Res. 2012;72: 4449–4458. 10.1158/0008-5472.CAN-11-4123 22815529 PMC3809830

[38] Fei DL, Li H, Kozul CD, Black KE, Singh S, Gosse JA, et al Activation of Hedgehog signaling by the environmental toxicant arsenic may contribute to the etiology of arsenic-induced tumors. Cancer Res. 2010;70: 1981–1988. 10.1158/0008-5472.CAN-09-2898 20179202 PMC2831120

[39] Brosseau JP, Lucier JF, Lapointe E, Durand M, Gendron D, Gervais-Bird J, et al High-throughput quantification of splicing isoforms. RNA. 2010;16: 442–449. 10.1261/rna.1877010 20038630 PMC2811672

