# Supplementary material for: Wild-Type U2AF1 Antagonizes the Splicing Program Characteristic of U2AF1-Mutant Tumors and Is Required for Cell Survival
Source: PLoS Genet. 2016 Oct 24;12(10):e1006384. doi: 10.1371/journal.pgen.1006384 (PMC5077151; doi:10.1371/journal.pgen.1006384)
Supplement: S1 Text — (DOCX) [file pgen.1006384.s001.docx]

**Supplemental Materials and Methods**

***Plasmid DNA***

*Plasmids for engineering the U2AF1*S34F *mutation*. The plasmids used for creating the endogenous S34F mutation in HBEC3kt cells were custom designed and synthesized by Transposagen. They include the donor vector (pU2AF1-S34F_Target), the TALEN (Transcription Activator-like Effector Nucleases)-expressing vectors (pU2AF1-FXTN, pU2AF1-RXTN) and the *PiggyBac*-expressing vector (pCMV-Pbo).

*shRNA constructs*. The shRNA constructs for *U2AF1* knockdown were obtained from OpenBiosystems (shU2AF1#1, TRCN0000001155; shU2AF1#4, TRCN0000001158). The control shRNAs (shScbr and shGFP) were described previously [1].

*Lentiviral constructs for overexpression of transgenes*. The lentiviral constructs for overexpressing mutant or WT U2AF1 were constructed in multiple steps. Firstly, the open reading frame of mouse *U2af1* (GenBank BC115479) was PCR amplified from a cDNA clone and cloned into the pENTR/D-TOPO vector (Invitrogen) following vendor’s instruction. Because mouse U2af1 (RefSeq NP_077149) is identical to human U2AF1 (RefSeq NP_006749) except lacking a Gly in the Gly rich domain (amino acid residues 212-222), a codon for Gly (GGA) was inserted into the corresponding *U2af1* DNA sequence via site-directed mutagenesis (Agilent). The S34F (TCT to TTT) missense mutation was also created by site-directed mutagenesis. The resulting WT and S34F mutant *U2af1* constructs were then cloned into a lentiviral destination vector, pLenti-CMV-Blast-DEST (Addgene # 17451, [5]), via LR Gateway reaction (Invitrogen). Entry vectors expressing enhanced green fluorescence protein (pENTR1A-GFP-N2, Addgene #19364, [2]) or DsRed-Express 2 [3] was used to clone the GFP or DsRed-Express 2 sequence into the same destination vector.

*pU2AF1-WT-S34F-e2*. This plasmid was used to serve as a reference to quantify the S34F:WT *U2AF1* ratio via quantitative PCR (qPCR). It was constructed by inserting a WT *U2AF1* exon 2 DNA fragment (GRCh37/hg19, Chr21: 44524425-44524512) or the same sequence with the Ser34 codon mutated to Phe34 (TCT to TTT) into the miniTK-Luc plasmid [4]. One copy of the wild-type *U2AF1* sequence was inserted 5´ of the *Luciferase* gene sequence via the Kpn I site. One copy of the mutant U2AF1 sequence was inserted 3´ of the *Luciferase* gene sequence via the BamH I site.

*CRISPR-Cas9 constructs*. The plasmids for expressing Cas9 and sgRNA-WT or sgRNA-S34F were constructed by inserting the relevant sgRNA sequences (WT, GTCATGGAGACAGGTGCTCT; S34F, GTCATGGAGACAGGTGCTTT) into lentiCRISPR (Addgene #49535, [5]) via a method described by the depositing investigator. The same lentiviral vector backbone with an sgRNA against GFP was obtained from Addgene (#51760, [5]).

pU2AF1-WT. Site-direct mutagenesis was performed on the donor vector, pU2AF1-S34F_Target, to convert S34F mutation to WT sequence (TTT to TCT). Both the pU2AF1-S34F_Target and pU2AF1-WT were used to characterize the specificity of the allele-sensitive S34F/WT SNP Taqman assay in Supplemental Fig. S5.

pCR-5´Probe and pCR-3´Probe. Genomic DNA fragments corresponding to the 5´and 3´ probe sequences (Supplemental Fig. S4) were PCR amplified by the primers (5´probe-forward, CGCTGAAAGTTGCCTGGAG; 5´probe-reverse, TAAACTACAGCCAACTCACTGG; 3´probe-forward, GATTTATGAACCGAATCTCCC; 3´probe-reverse, TCCATAAGCCGCCAAGTAC), and cloned into the pCR2.1-TOPO or pCR-Blunt vector. For Southern blot analysis, the probe fragments were released from the vectors by EcoR I digestion, separately by agarose gel electrophoresis, and purified by a gel extraction kit (Qiagen).

All plasmids mentioned above were sequence verified.

***Genome editing approaches***

The creation of a *U2AF1*S34F allele from the endogenous *U2AF1* locus was conducted as described in Fig. 2A following an previously reported method [6]. The donor vector (pU2AF1-S34F_Target) contains the S34F missense mutation (TCT to TTT) flanked by homology arm sequences (HA) of roughly 1000 bp on each side of the point mutation. A PGK-Hygro∆TK drug cassette, flanked by *PiggyBac* recognition sequences (ITR, inverted terminal repeat), was located 3´ of the S34F mutation at an endogenous AATT site that is essential for *PiggyBac* recognition. The homologous recombination was facilitated by cutting the *U2AF1* genomic DNA by a pair of TALENs, which bind to the *U2AF1* genomic sequences located in intron 2 (underlined):

TAAGTGTGTTCTTTTATTTAAATAATAGTGAGGCAGGTGATCGACTTCCA

The donor vector and the TALENs were co-transfected into HBEC3kt cells in 6-well dishes by Lipofectamine 2000 (Invitrogen). The cells were transferred to 150 mm dishes and cultured in growth media containing Hygromycin B (50 µg/ml, Invitrogen). Cell clones (≥ 50 cells) were harvested using cloning rings (Thermo Fisher Scientific) and expanded. Genomic DNA from these clones were harvested and screened for mutant and WT intermediate clones by Sanger sequencing and Southern blot as described in Supplemental Fig. S4 (P1-F, TGTCCTAATTCATCAGAGATCG; P1-R, TAAACCTCGATATACAGACCGATA). The correct intermediate cell clones were co-transfected with plasmids expressing an excision-only *PiggyBac* transposase (pCMV-Pbo), to remove the PGK-Hygro∆TK drug cassette, and eGFP (pEGFP-C1). GFP positive cells were sorted on a BD FACSAria and cultured in the presence of Ganciclovir (10 µg/ml, Sigma). Cell clones were isolated by cloning rings and expanded. The DNA and RNA from these cells were used to confirm the removal of the drug cassette (by genomic PCR using the P1-F and P1-R primers as described in Supplemental Fig. S4), as well as expression of *U2AF1*S34F (by allele-specific qPCR and RNA-seq).

Disruption of the wild-type or mutant *U2AF1* alleles from H441, HCC78 and MUT1a cells were conducted by transducing these cells with lentiviruses expressing Cas9 and either sgRNA-WT or sgRNA-S34F. Virus-infected cells were selected by puromycin (1 µg/ml) except in HBEC3kt-derived cells since HBEC3kt cells were intrinsically resistant to puromycin [7]. A separate transduction with GFP lentiviruses at a similar titer confirmed that the infection efficiency in HBEC3kt-derived cells was nearly 100%. Polyclonal and clonal cells were derived from these infected cells. CRISPR-mediated induction of InDels in the *U2AF1* locus was confirmed by fluorescence PCR (data not shown) [8]. The disruption of the wild-type or mutant U2AF1 allele was further confirmed by changes in the ratios of S34F:WT gene products and subsequent changes in S34F-associated splicing.

***U2AF1 copy number analysis in LUAD***

The U2AF1 copy number status for all LUAD samples was annotated by GISTIC2.0 [9] and obtained directly from cBioPortal (www.cbioportal.org/). The copy number change is defined as possessing deletion (-2), shallow deletion (-1), diploid (0), gain (+1) and high gain (+2) in each sample using sample-specific thresholds. "Deletion", copy numbers that are below the minimum median chromosomal arm copy number for that sample by at least 0.1; "Shallow deletion", copy numbers between the “Deletion” threshold and 1.9; "Diploid", copy numbers between 1.9 and 2.1; "Gain", copy numbers between 2.1 and the “High Gain” threshold; "High Gain", copy numbers that exceed the maximum median chromosomal arm copy number for that sample by at least 0.1.

***Southern Blot***

Southern blot was performed as previously described [10]. Briefly, high molecular weight genomic DNA was extract from cells using a lysis buffer (10 mM Tris, pH 8.0, 100 mM NaCl, 10 mM EDTA, 0.5% SDS, 0.2 µl/ml protease K), followed by phenol/chloroform extraction, isopropanol precipitation, and washing with 70% ethanol. DNA was dissolved in TE buffer (10 mM Tris, pH 8.0, 1 mM EDTA). Up to 25 µg DNA was digested by the appropriate restriction enzymes (Supplemental Fig. S4), separated on a 1% agarose gel, and transferred onto positive-charged nylon membranes (Ambion) using an alkaline buffer (0.4 M NaOH, 1 M NaCl). The 5´and 3´ probe DNA fragments were ^32^P-labeled by the Ready-To-Go DNA Labelling Beads (-dCTP) (GE Healthcare) and hybridized to the nylon membrane. Radioactive signal was acquired by a phosphorimager (Fuji).

***RNA extraction, reverse transcription, and quantitative PCR (RT-qPCR)***

RNA was extracted using Trizol (Molecular Research Center or Invitrogen) followed by a column cleanup step using an RNeasy kit (Qiagen). cDNA was synthesized from 1-2 μg total RNA using a High-Capacity cDNA Reverse Transcription (RT) kit (Applied Biosystems). Quantitative PCR (qPCR) was done in a 7900HT real-time PCR system (Applied Biosystems) (other than those described in Supplemental Fig. S11). The mRNA level of total *U2AF1* and *GAPDH* was measured by inventoried Taqman assays (Hs01597469_m1, Hs99999905_m1, Applied Biosystems) in a standard Taqman qPCR master mix (Applied Biosystems). The allele-sensitive S34F/WT SNP Taqman assay were custom synthesized and was characterized in Supplemental Fig. S5.

Splicing alterations were measured by isoform-specific primers (listed in Supplemental Table S7) in a SYBR Green qPCR master mix (Affymetrix). These primers were designed following a previously described method using a web tool: http://designs.lgfus.ca/[11]. PCR efficiency of each primer set was estimated by performing the qPCR assay using at least four 10-fold serial dilutions of the cDNA template. The slope of the standard curve was then translated into a PCR efficiency value by the formula: (10^(-1/slope)^ – 1) x 100%. The PCR end products were separated on a 2% agarose gel to check specificity. The specificity of a subset of the primers was further confirmed by Sanger sequencing using the PCR end products. All the primers used in the study have a PCR efficiency of 85% or more and are specific for the targets they measure.

RT-qPCR results were quantified using a relative quantification method (∆∆Ct) in the RQ Manager 1.2 software (Applied Biosystems). In most cases, the reference sample was cells that underwent control treatment. For allele-sensitive S34F/WT SNP Taqman assay, the reference sample was the plasmid DNA pU2AF1-WT-S34F-e2, which contains one copy of each of the *U2AF1* WT and S34F mutant sequences.

A subset of splicing events, as described in Supplemental Fig. S11, were quantified by the QX200 Droplet Digital PCR system (ddPCR, Bio-Rad) using isoform-specific primers (listed in Supplemental Table S7). The concentration of each isoform was calculated in the QuantaSoft Software (Bio-Rad) and the presented as the relative ratio between two alternatively spliced isoforms.

**Bioinformatics**

MISO v2.0 annotations were used for all splicing analyses

[12], as was a set of annotated constitutive junctions defined as junctions with no evidence of alternative splicing in the UCSC knownGene database [13]. A genome annotation for read mapping was created by merging transcript annotations from MISO v2.0 annotations

[12], the UCSC knownGene database and the Ensembl 71 database [14]. Reads were first mapped to these transcripts using RSEM [15]. Remaining unaligned reads were then mapped to a database of possible junctions between all 5' and 3' splice sites of those transcripts, and then subsequently to the GRCh37/hg19 human genome assembly, using TopHat [16].

Differentially spliced events were identified using MISO to quantify reads supporting distinct isoforms and/or junction-spanning reads. These read counts were then subjected to Wagenmakers’s Bayesian alternative to the binomial proportion test [17]. Differentially spliced events were defined as those that exhibited a difference in absolute isoform ratio of at least 10% with an associated Bayes factor of at least 5. Most analyses were restricted to events with at least 20 relevant reads in the samples being compared. The exceptions were for estimating the inclusion levels of the 5' extensions of exons in *FMR1* and *CASP8* mRNA (Supplemental Fig. S3, panels **M** - **T**), for which only 10 or more relevant reads were required.

Sequences logos were created using the seqLogo package from Bioconductor [18]. The invariant AG at the 3' splice site was not plotted in scale in order to highlight the consensus sequences at the -3 position. Cluster analysis was performed using Ward’s method. The information of percent tumor nuclei for each LUAD sample that carries a *U2AF1*S34F mutation was downloaded from TCGA data portal (http://cancergenome.nih.gov/). For samples with more than one percent tumor nuclei value, an average value was calculated and used for comparisons.

Relative expression of the S34F and WT alleles was estimated from the mapped RNA-seq data by identifying aligned reads that overlapped the *U2AF1*S34 codon and then counting the numbers of reads that matched the TTT versus TCT codons.

***Purification of U2AF1 protein complexes***

The U2AF2 protein subunit (residues 85–471, where residue 471 is the C-terminus of U2AF2 isoform b, NCBI RefSeq NP_001012496) was co-expressed with either the wild-type U2AF1 subunit (residues 1–193 of NCBI RefSeq NP_006749) or the S34F mutant subunit, in *E coli* as respective fusions with GST (in pGEX-6p2) or MBP (in a variant of pCDF-1b). The U2AF1 and U2AF2 proteins are full length with the exception of nonspecific RS domains and the SF1 protein includes the U2AF2- and RNA-binding domains. Prior to mixing the subunits, MBP-tag was cleaved and separated from the U2AF-heterodimer by GST affinity chromatography. Next, the GST-tag was cleaved and separated from the U2AF-heterodimer by a subtractive GST affinity step. The SF1 protein subunit (residues 1–255 of NCBI RefSeq NP_004621) was expressed separately in *E coli* as a GST fusion; the GST-tag was cleaved from SF1 and separated by cation-exchange chromatography. The purified U2AF-heterodimer (containing either wild-type or S34F mutant U2AF1) was mixed with the SF1 subunit to prepare the ternary protein complexes by size-exclusion chromatography in 25 mM HEPES pH 6.8, 150 mM NaCl, 3% glycerol, 20 μM ZnCl_2_, 3 mM βME and 0.5 mM TCEP. The final step of size-exclusion chromatography ensured homogenous complexes for affinity determination.

***RNA affinity measurement of purified U2AF1 complexes***

The recombinant U2AF1 complexes comprised either wild-type or S34F mutant U2AF1 (residues 1-193), with two additional proteins, U2AF2 (residues 85-471 of isoform b) and SF1 (residues 1-255). Sequences of synthetic 5′-labeled fluorescein RNAs (GE Healthcare Dharmacon) are given in the Supplementary Fig. S12. Both the protein complex and the RNA stocks were prepared and diluted in the same buffer used for size-exclusion chromatography. Fluorescence anisotropy changes were monitored over a concentration series of purified U2AF1 protein complexes (final concentrations shown in Supplementary Fig. S12) mixed with fluorescein-labeled RNAs (20 nM final concentration) in 384-well flat bottom assay plates (Corning). Fluorescence polarization changes were measured at 520 n following excitation at 490 nm using Envision high-throughput plate reader (PerkinElmer). The data were fit by non-linear regression to obtain the apparent equilibrium dissociation constant (*K_D_*) using the following equation, where *X* is the total protein concentration, [RNA] is the total RNA concentration, *r* is the observed anisotropy at the *i^th^* titration, *r_B_* is the anisotropy at zero protein concentration, and *r_F_* is the anisotropy at saturating protein concentration:

$$r=r_{F}+\frac{r_{B}-r_{F}}{2\left[ RNA \right]} \left( K_{D}+X+[RNA] \right)- \sqrt{{(K_{D}+X+\left[ RNA \right])}^{2}-\left( 4\left[ RNA \right]X \right)}$$

The reciprocal of the K_D_ value provided the apparent equilibrium affinity constant (K_A_).

***Xenograft tumors in nude mice***

The mouse study was approved by and done in accordance with the policies of the Animal Care and Use Committee at the National Human Genome Research Institute (Protocol #G-10-6 and G-13-1). CD-1 nude mice (7 week old, male or female, Charles River) were housed for one more week upon delivery before tumor cell inoculation. One to five million live cells were mixed with an equal volume of Matrigel (5 mg/ml, BD Biosciences) and injected subcutaneously in the flanks of nude mice. The width (W) and length (L) of the tumor were measured by a caliper. Tumor volume was estimated by the formula (W×W×L)/2 as described previously [1].

**References**

1. Fei DL, Sanchez-Mejias A, Wang Z, Flaveny C, Long J, Singh S, et al. Hedgehog Signaling Regulates Bladder Cancer Growth and Tumorigenicity. Cancer Res. 2012;72: 4449–4458. doi:10.1158/0008-5472.CAN-11-4123

2. Campeau E, Ruhl VE, Rodier F, Smith CL, Rahmberg BL, Fuss JO, et al. A versatile viral system for expression and depletion of proteins in mammalian cells. PLoS ONE. 2009;4: e6529. doi:10.1371/journal.pone.0006529

3. Strack RL, Strongin DE, Bhattacharyya D, Tao W, Berman A, Broxmeyer HE, et al. A noncytotoxic DsRed variant for whole-cell labeling. Nat Methods. 2008;5: 955–957. doi:10.1038/nmeth.1264

4. Fei DL, Li H, Kozul CD, Black KE, Singh S, Gosse JA, et al. Activation of Hedgehog signaling by the environmental toxicant arsenic may contribute to the etiology of arsenic-induced tumors. Cancer Res. 2010;70: 1981–1988. doi:10.1158/0008-5472.CAN-09-2898

5. Shalem O, Sanjana NE, Hartenian E, Shi X, Scott DA, Mikkelsen TS, et al. Genome-Scale CRISPR-Cas9 Knockout Screening in Human Cells. Science. 2014;343: 84–87. doi:10.1126/science.1247005

6. Yusa K. Seamless genome editing in human pluripotent stem cells using custom endonuclease-based gene targeting and the piggyBac transposon. Nat Protoc. 2013;8: 2061–2078. doi:10.1038/nprot.2013.126

7. Ramirez RD, Sheridan S, Girard L, Sato M, Kim Y, Pollack J, et al. Immortalization of human bronchial epithelial cells in the absence of viral oncoproteins. Cancer Res. 2004;64: 9027–9034. doi:10.1158/0008-5472.CAN-04-3703

8. Carrington B, Varshney GK, Burgess SM, Sood R. CRISPR-STAT: an easy and reliable PCR-based method to evaluate target-specific sgRNA activity. Nucleic Acids Res. 2015;43: e157. doi:10.1093/nar/gkv802

9. Mermel CH, Schumacher SE, Hill B, Meyerson ML, Beroukhim R, Getz G. GISTIC2.0 facilitates sensitive and confident localization of the targets of focal somatic copy-number alteration in human cancers. Genome Biol. 2011;12: R41. doi:10.1186/gb-2011-12-4-r41

10. Avilés Mendoza GJ, Seidel NE, Otsu M, Anderson SM, Simon-Stoos K, Herrera A, et al. Comparison of five retrovirus vectors containing the human IL-2 receptor gamma chain gene for their ability to restore T and B lymphocytes in the X-linked severe combined immunodeficiency mouse model. Mol Ther. 2001;3: 565–573. doi:10.1006/mthe.2001.0292

11. Brosseau JP, Lucier JF, Lapointe E, Durand M, Gendron D, Gervais-Bird J, et al. High-throughput quantification of splicing isoforms. RNA. 2010;16: 442–449. doi:10.1261/rna.1877010

12. Katz Y, Wang ET, Airoldi EM, Burge CB. Analysis and design of RNA sequencing experiments for identifying isoform regulation. Nat Methods. 2010;7: 1009–1015. doi:10.1038/nmeth.1528

13. Meyer LR, Zweig AS, Hinrichs AS, Karolchik D, Kuhn RM, Wong M, et al. The UCSC Genome Browser database: extensions and updates 2013. Nucleic Acids Res. 2013;41: D64–9. doi:10.1093/nar/gks1048

14. Flicek P, Ahmed I, Amode MR, Barrell D, Beal K, Brent S, et al. Ensembl 2013. Nucleic Acids Res. 2013;41: D48–55. doi:10.1093/nar/gks1236

15. Li B, Dewey CN. RSEM: accurate transcript quantification from RNA-Seq data with or without a reference genome. BMC Bioinformatics. 2011;12: 323. doi:10.1186/1471-2105-12-323

16. Trapnell C, Pachter L, Salzberg SL. TopHat: discovering splice junctions with RNA-Seq. Bioinformatics. 2009;25: 1105–1111. doi:10.1093/bioinformatics/btp120

17. Wagenmakers E-J, Lodewyckx T, Kuriyal H, Grasman R. Bayesian hypothesis testing for psychologists: a tutorial on the Savage-Dickey method. Cogn Psychol. 2010;60: 158–189. doi:10.1016/j.cogpsych.2009.12.001

18. Gentleman RC, Carey VJ, Bates DM, Bolstad B, Dettling M, Dudoit S, et al. Bioconductor: open software development for computational biology and bioinformatics. Genome Biol. 2004;5: R80. doi:10.1186/gb-2004-5-10-r80
