# Supplementary material for: Wild-Type U2AF1 Antagonizes the Splicing Program Characteristic of U2AF1-Mutant Tumors and Is Required for Cell Survival
Source: PLoS Genet. 2016 Oct 24;12(10):e1006384. doi: 10.1371/journal.pgen.1006384 (PMC5077151; doi:10.1371/journal.pgen.1006384)

A. "Typical S34F" consensus 3' splice sites from mutant LUAD Transcriptomes

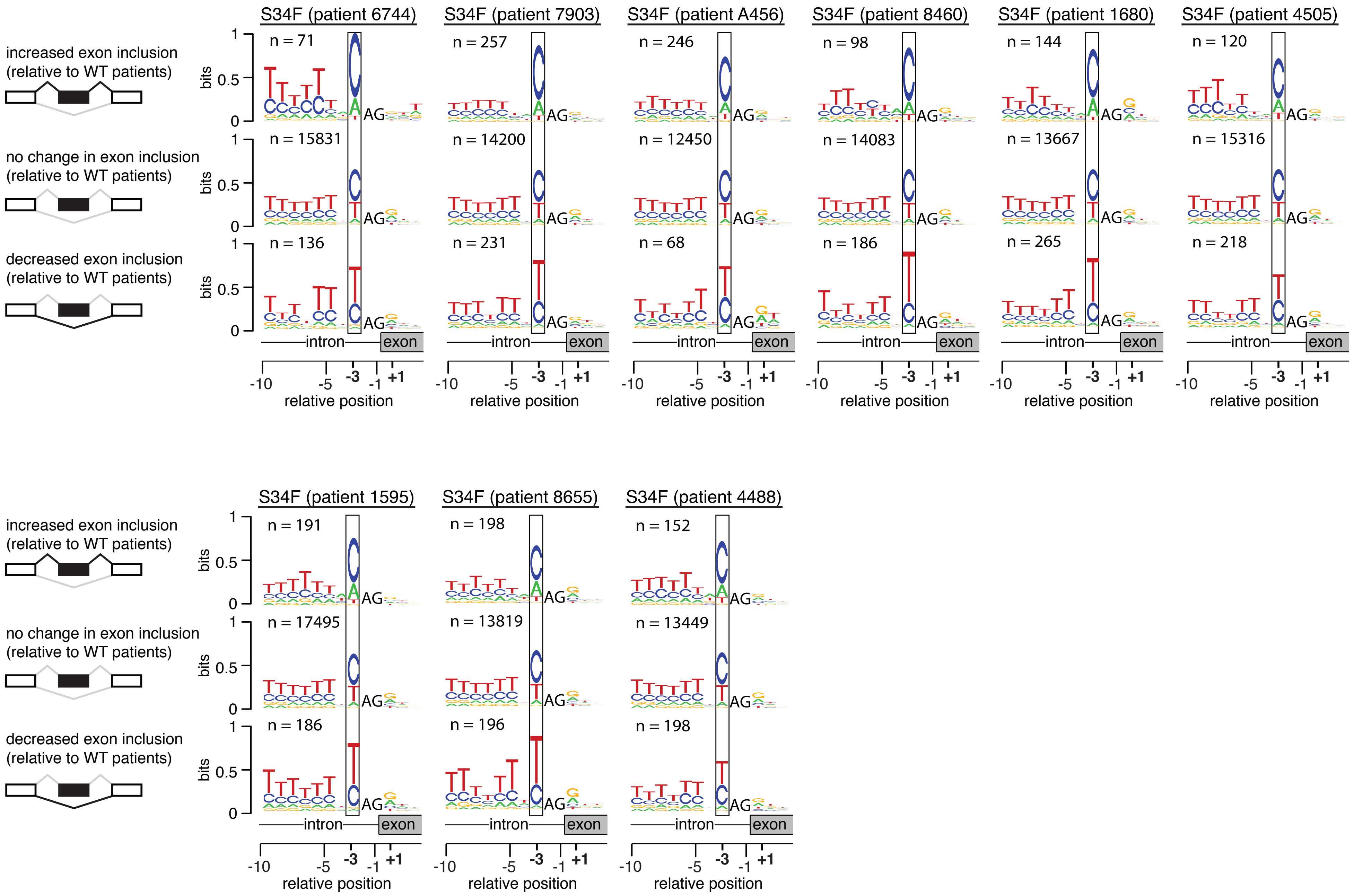

(to be continued on the next page)

B. “Quasi-WT” consensus 3' splice sites from mutant LUAD Transcriptomes

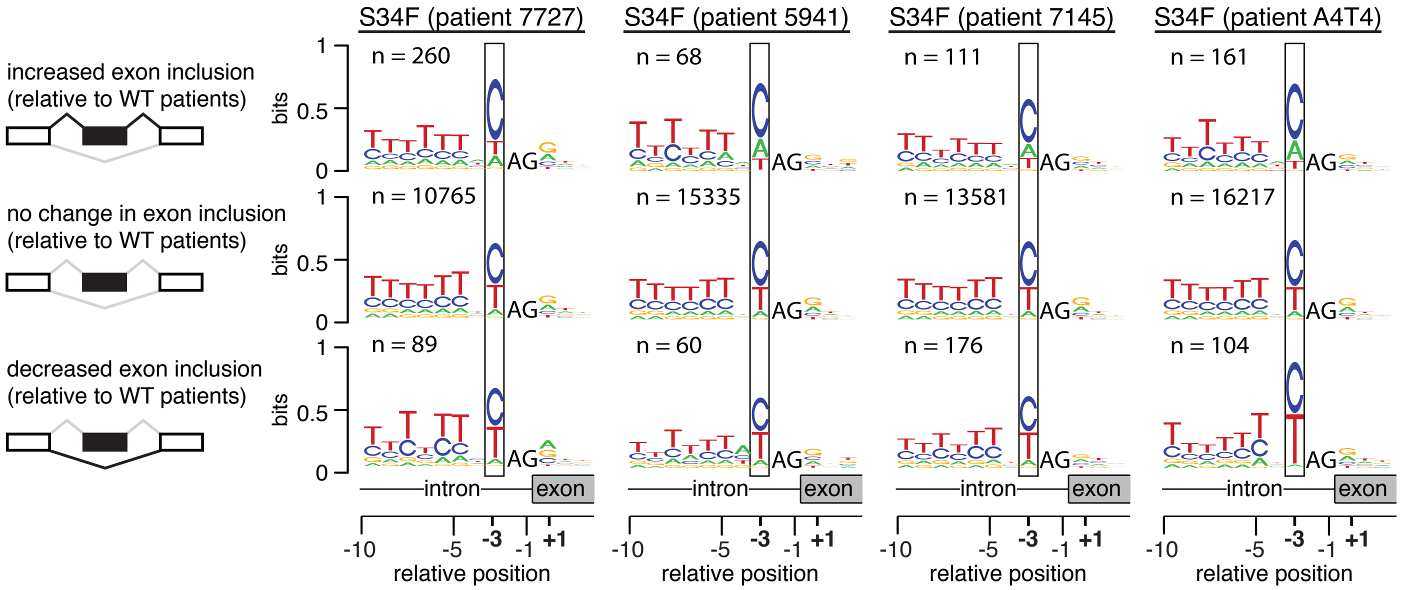

(to be continued on the next page)

C. Wild-type consensus 3' splice sites from wild-type LUAD Transcriptomes

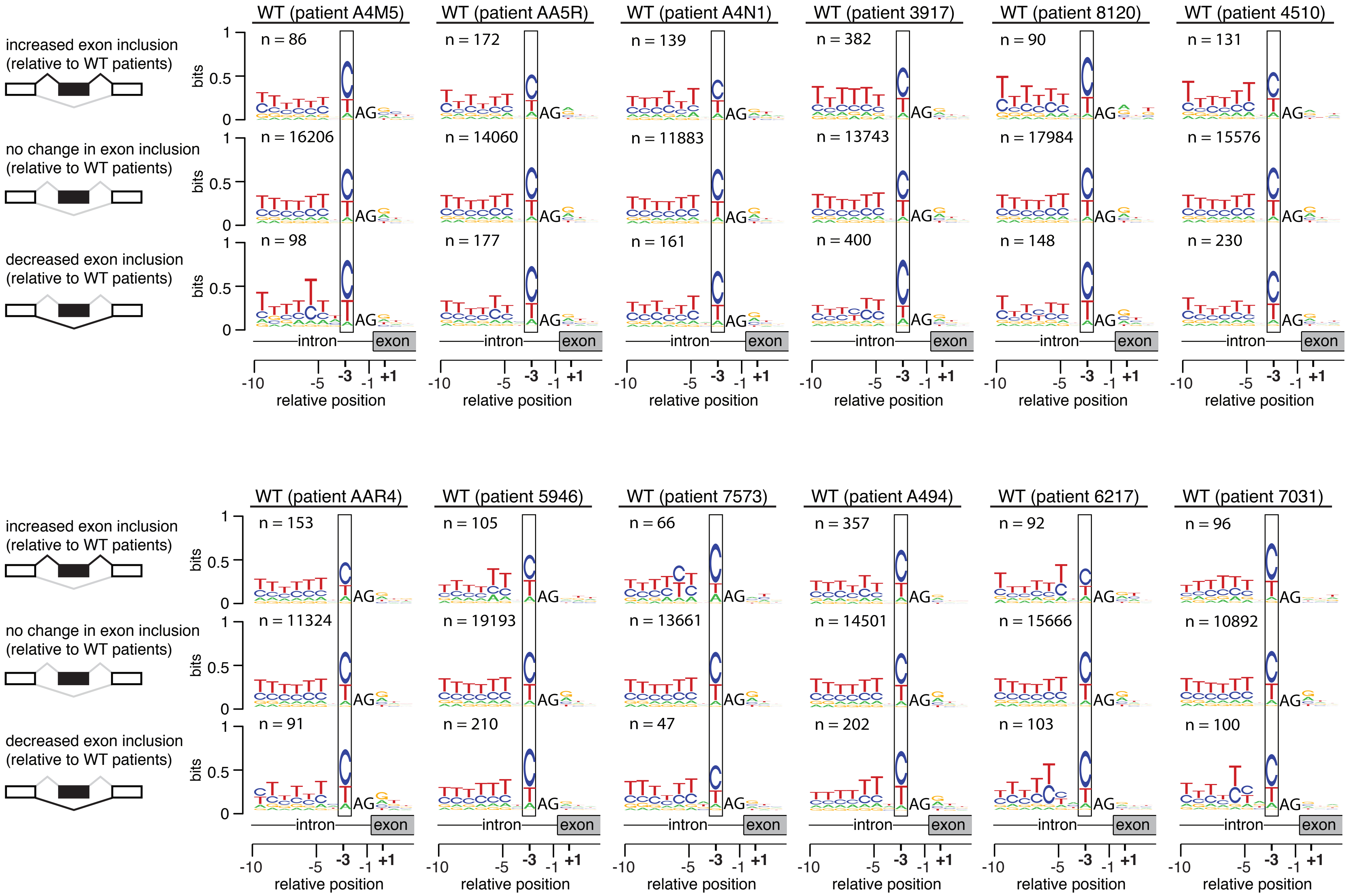

(to be continued on the next page)

C. Wild-type consensus 3' splice sites from wild-type LUAD Transcriptomes

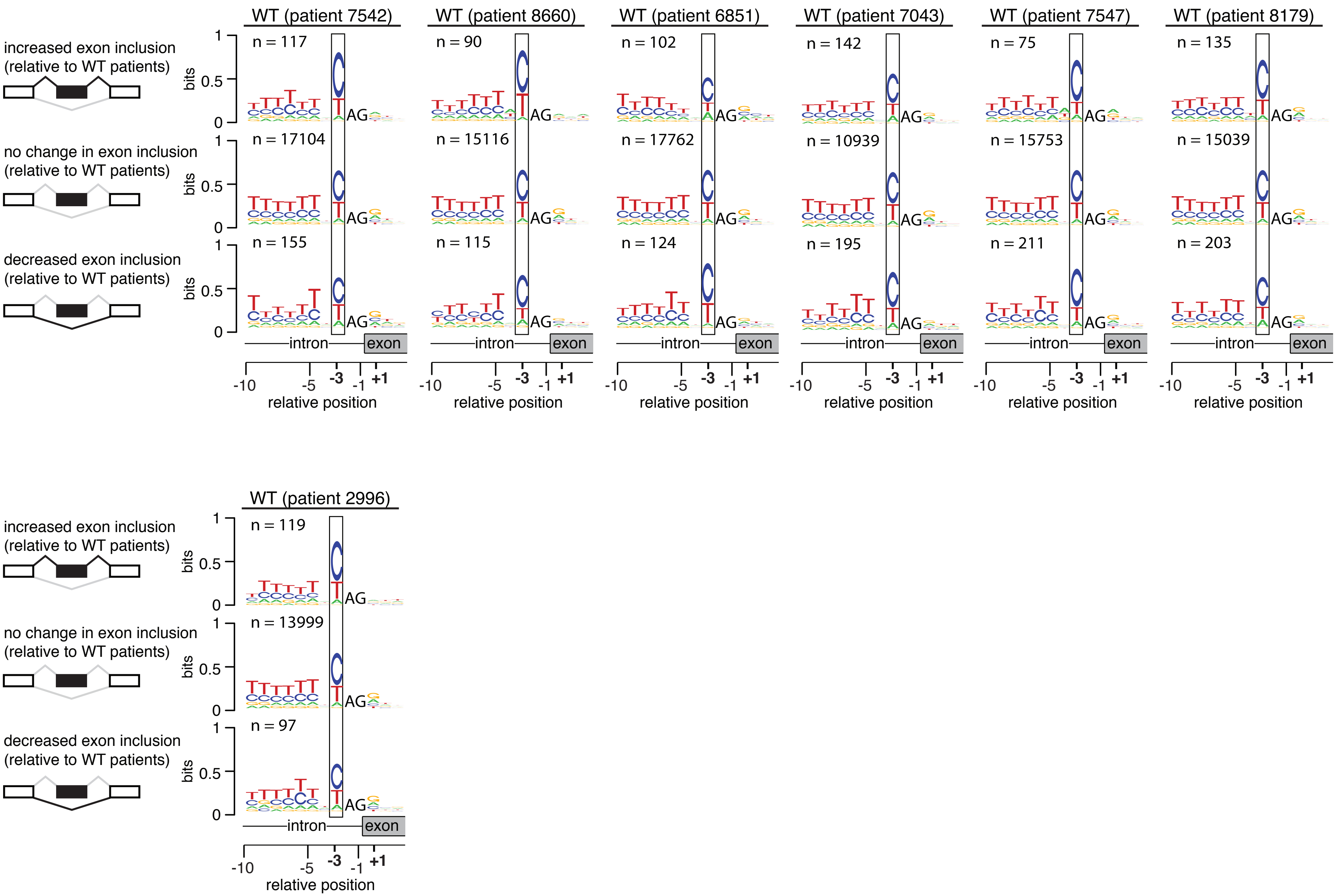

Supplement: S1 Fig — As in Fig 1A, sequence logos of the region flanking 3′ splice sites were computed from LUAD transcriptomes reported by TCGA. Panel A: Logos from tumors carrying a U2AF1S34F mutation and showing “typical S34F” consensus 3′ splice sites. Panel B: Logos from tumors carrying the U2AF1S34F mutation but with compromised S34F-associated features at the -3 position (“quasi-WT” pattern). Panel C: Logos from tumors without a U2AF1 mutation. Sequence logos deduced from tumor transcriptomes #7903 and #7727 are also displayed in Fig 1A. (PDF) [file pgen.1006384.s002.pdf]
