# Supplementary figures and images for: Wild-Type U2AF1 Antagonizes the Splicing Program Characteristic of U2AF1-Mutant Tumors and Is Required for Cell Survival
Source: PLoS Genet. 2016 Oct 24;12(10):e1006384. doi: 10.1371/journal.pgen.1006384 (PMC5077151; doi:10.1371/journal.pgen.1006384)

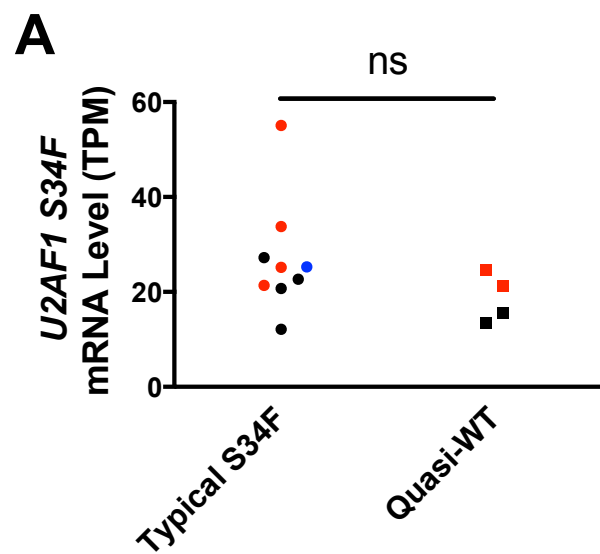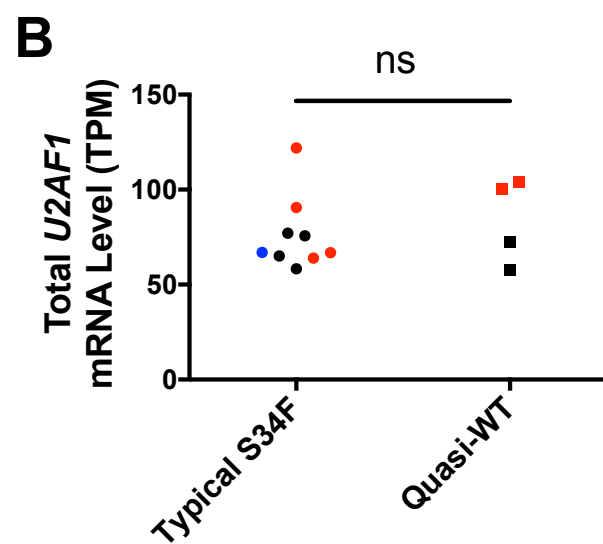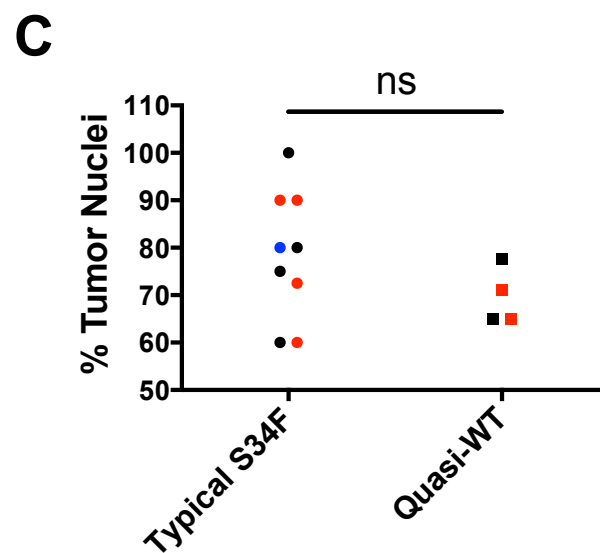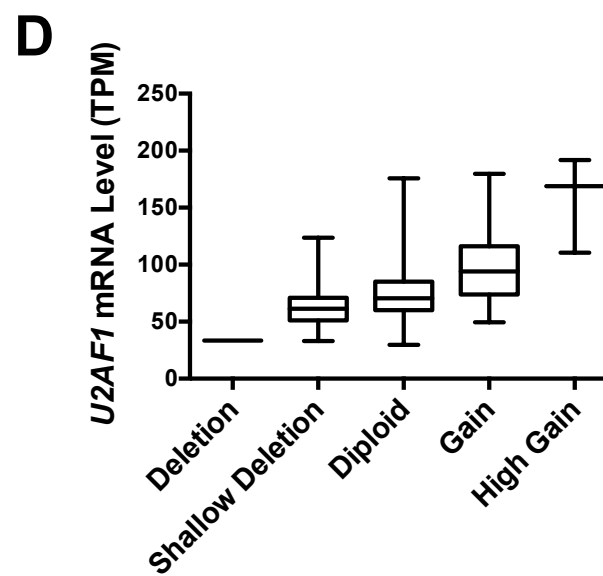

Supplement: S2 Fig — Tumors with transcriptomes that display “typical S34F” or “quasi-WT” consensus 3′ splice sites (Figs 1A, S1A and S1B) are plotted to compare with the level of U2AF1S34F mRNA (panel A), the level of total U2AF1 mRNA (panel B), or the percentage of tumor nuclei in the tissue samples (panel C). The definitions of the shape and color of each dot are the same as in Fig 1, panels B—D. (D), The amount of U2AF1 mRNA is displayed in relation to gains and losses of U2AF1 DNA copy number. Box indicates U2AF1 mRNA levels from the 25th to 75th percentiles within the group. The median value is represented as a line in box. Error bars represent the range of values. TPM, transcripts per million. ns, not significant as determined by student’s t test (p value > 0.05). (PDF) [file pgen.1006384.s003.pdf]

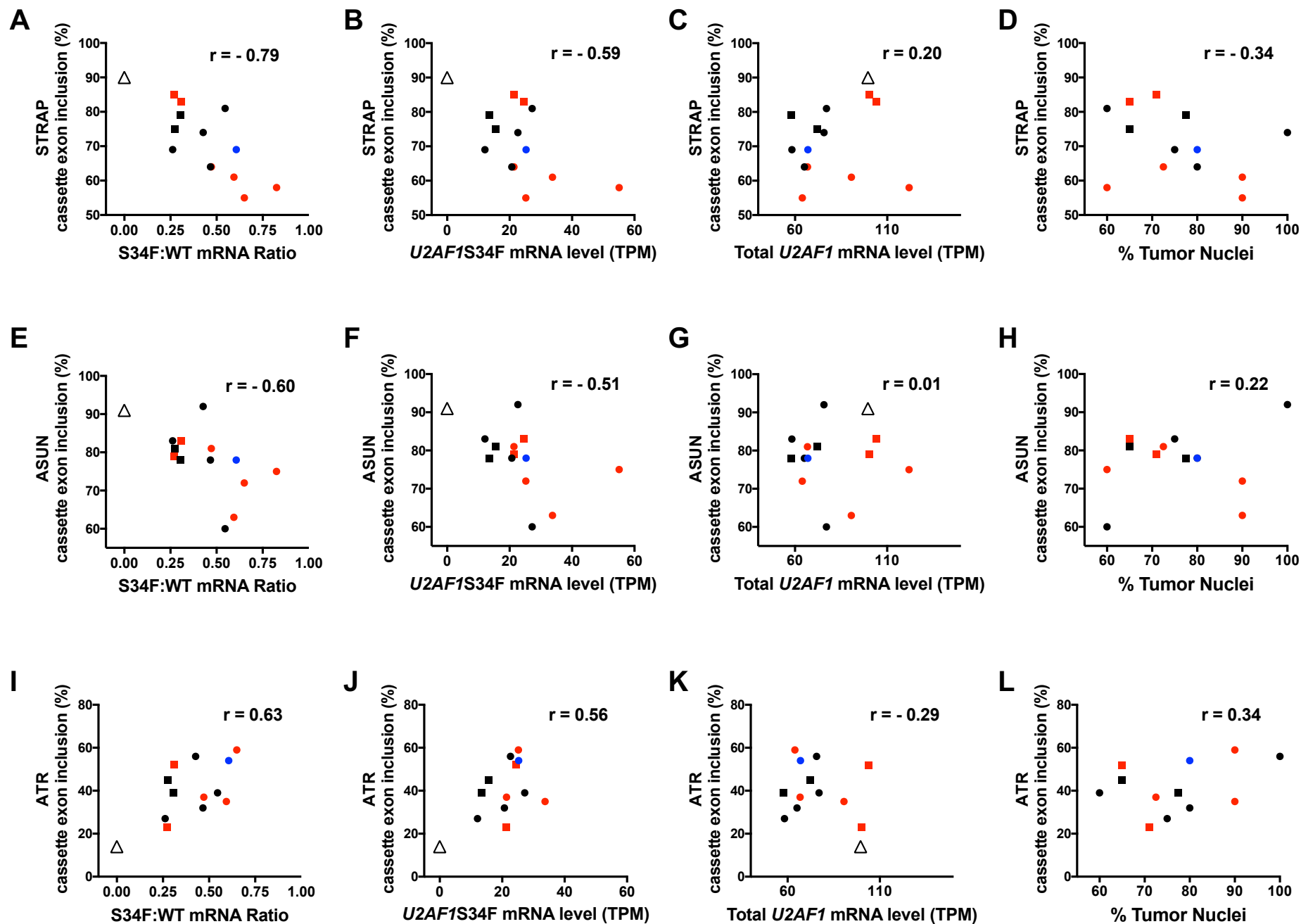

(to be continued on the next page)

**M**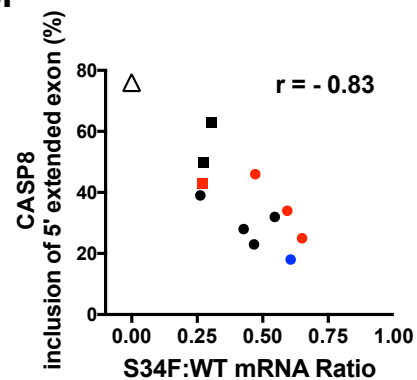**N**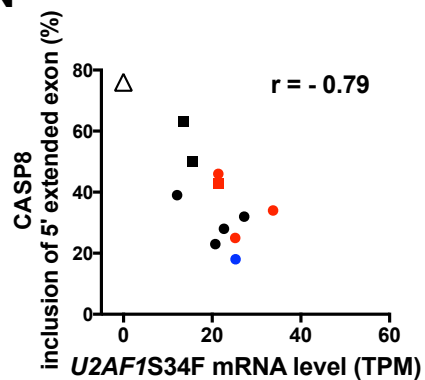**O**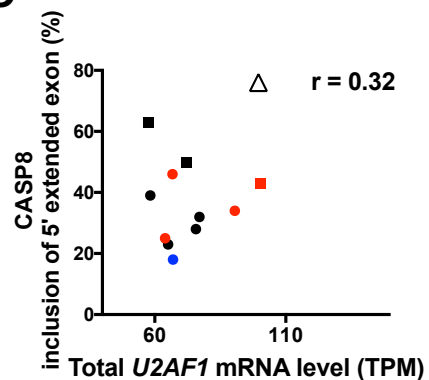**P**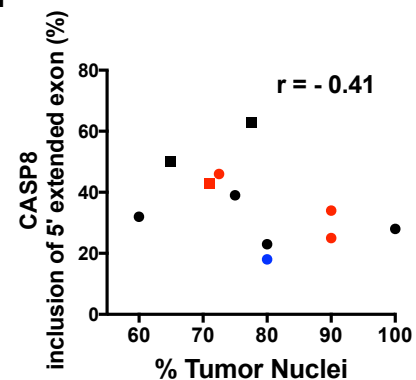**Q**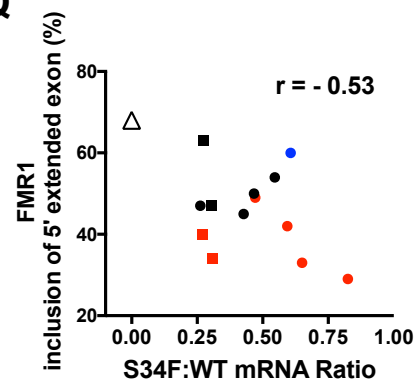**R**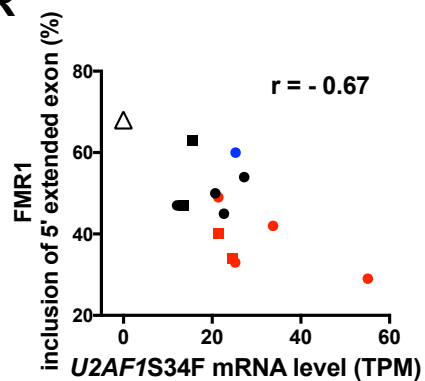**S**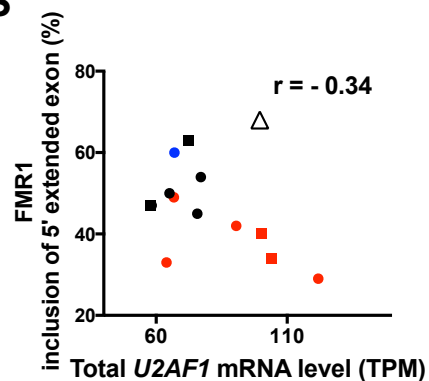**T**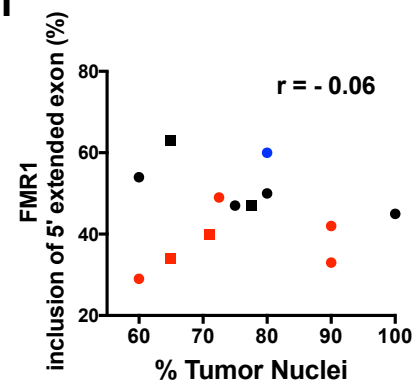

Supplement: S3 Fig — Pearson’s correlation was calculated to seek relationships between the inclusion levels of five representative splicing events and four factors in several LUAD tumors in the TCGA data set: the S34F:WT mRNA ratio, the level of U2AF1S34F mRNA, total U2AF1 mRNA, or percent tumor nuclei. Panels A–D, cassette exon in STRAP mRNA that is preferentially skipped in the presence of the U2AF1S34F mutation. Panels E–H, cassette exon in ASUN mRNA that is preferentially skipped in the presence of the U2AF1 S34F mutation; Panels I–L, cassette exon in ATR mRNA that is preferentially included in the presence of the U2AF1S34F mutation; Panels M–P, the 5' extension of the FMR1 exon that is less frequently included in the presence of the U2AF1S34F mutation; Panel Q–T, the 5' extension of the CASP8 exon that is less frequently included in the presence of the U2AF1S34F mutation. U2AF1-mutant LUAD transcriptomes containing sufficient number of informative reads to calculate the inclusion frequency were included in the analysis. The median usage of the indicated isoform for all transcriptomes from tumors without the mutation is shown as a triangle. The definitions of the shape and color of each dot are the same as in Fig 1, panels B—D. r, Pearson’s association coefficient. Panel A is also shown in Fig 1D. TPM, transcripts per million. (PDF) [file pgen.1006384.s004.pdf]

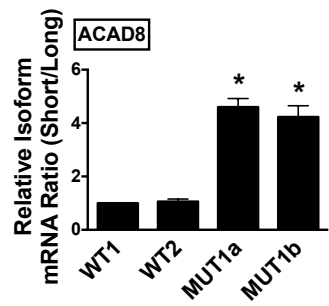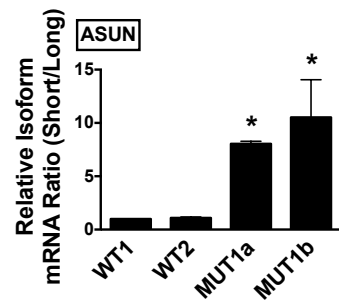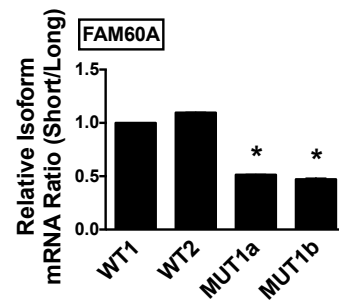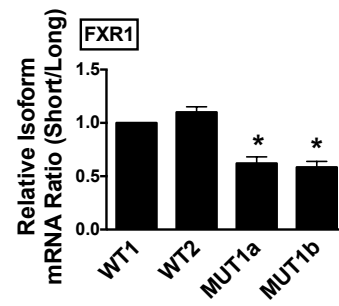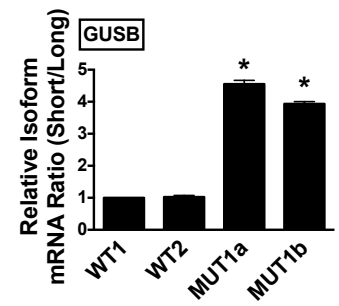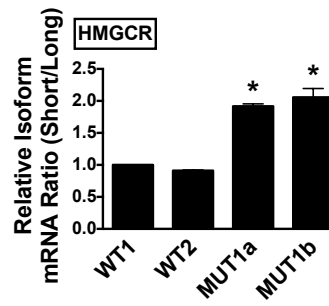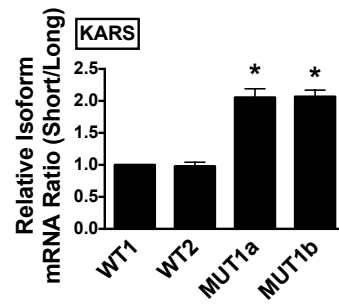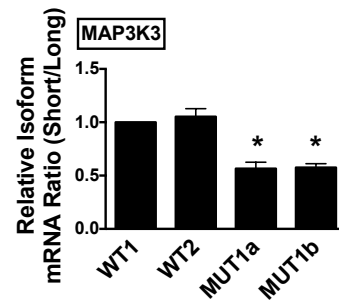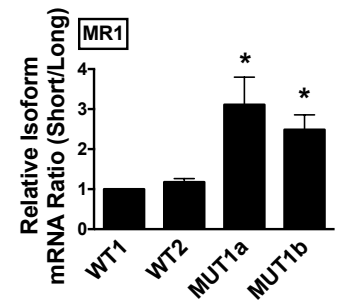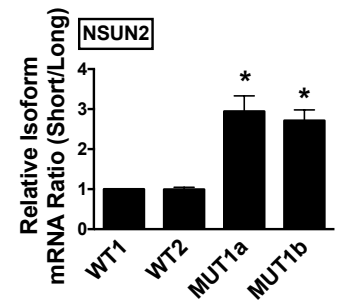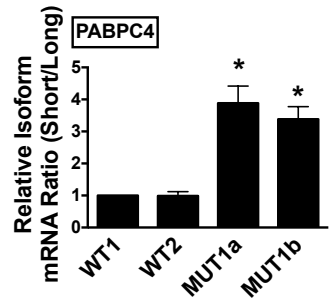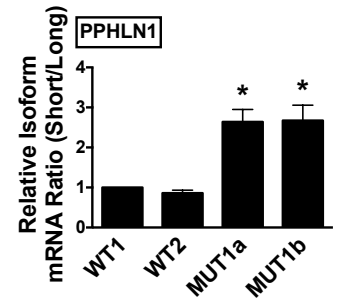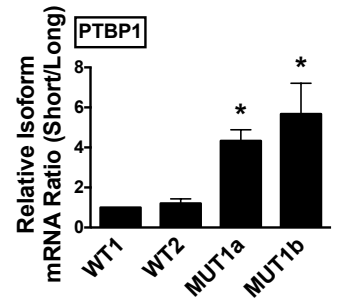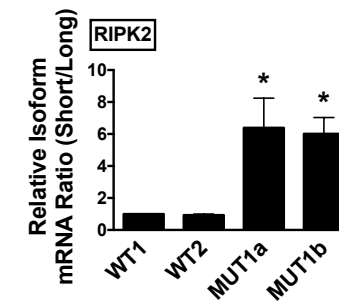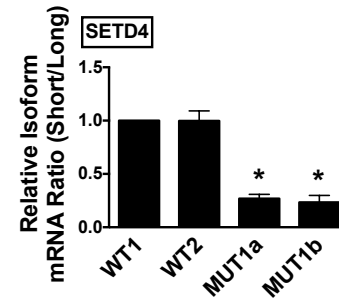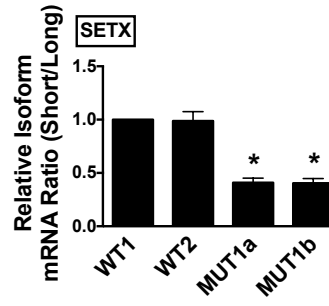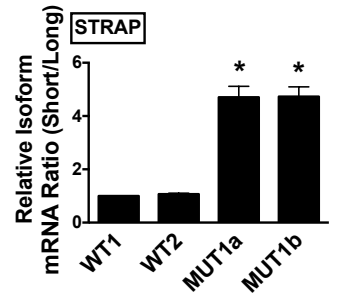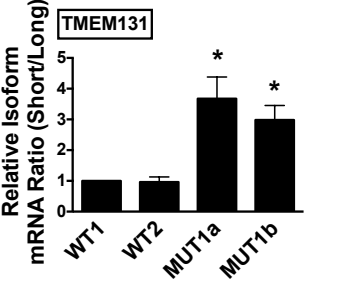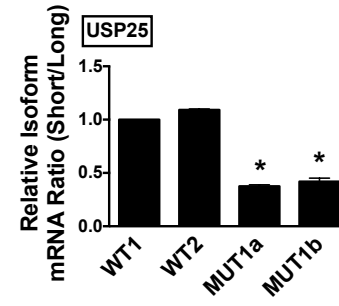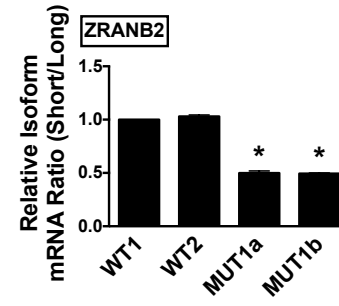

Supplement: S6 Fig — The relative amounts of short and long isoforms of the designated genes were measured by RT-qPCR to estimate changes in the inclusion of cassette exons, that were previously reported to be affected by the U2AF1S34F mutation as judged by RNA-seq of LUAD and AML tumor samples from TCGA [19]. The splicing alterations of the ASUN and STRAP cassette exons are also shown in Fig 2C. Asterisks represent statistically significant changes compared to WT1 cells. Error bars represent s.e.m. (n = 4). (PDF) [file pgen.1006384.s007.pdf]

**A**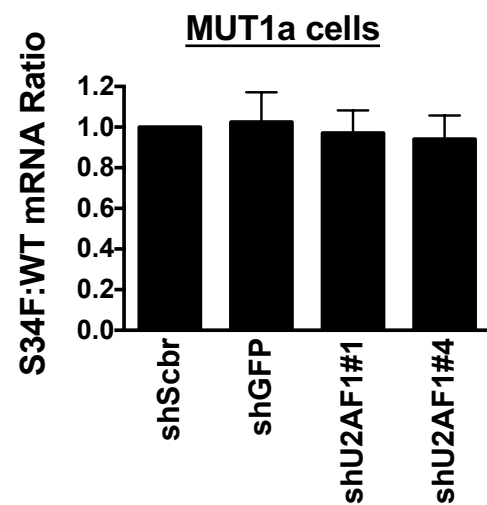**B**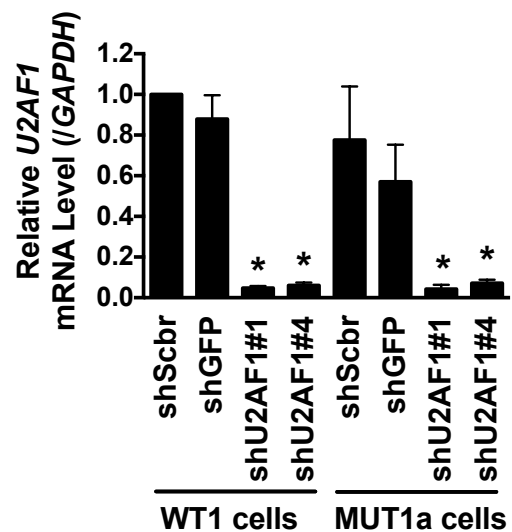**C**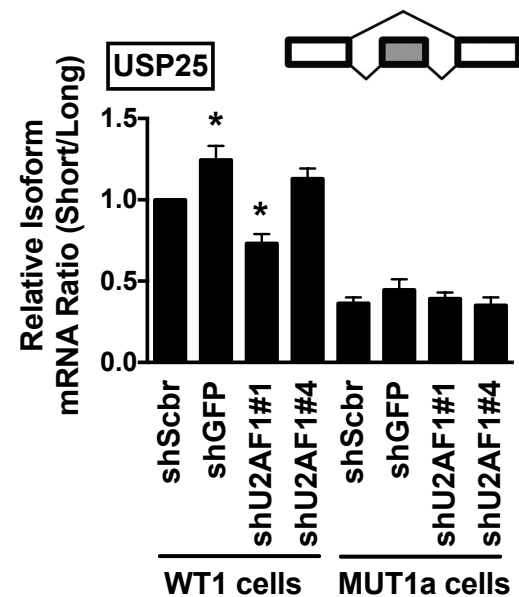**D**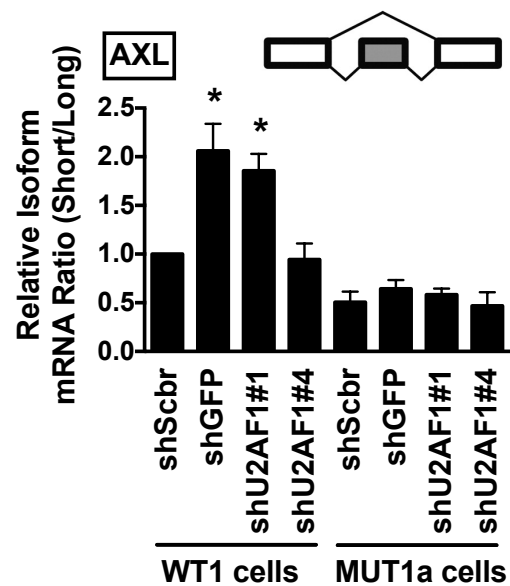**E**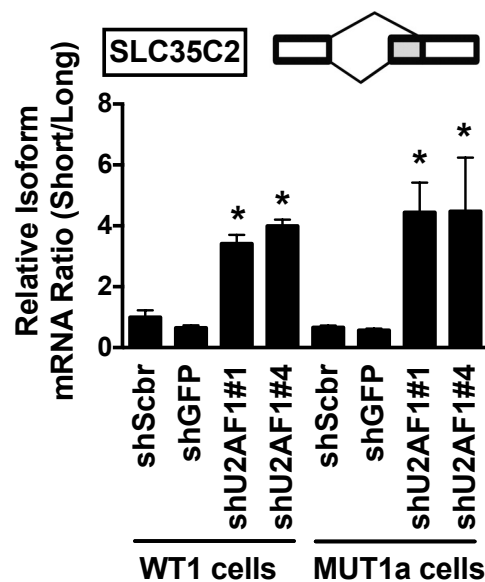

Supplement: S7 Fig — Additional assays used in the studies shown in Fig 3A were performed on WT1 and MUT1a cell lines. (A). Knockdown of total U2AF1 mRNA with the indicated shRNAs does not affect S34F:WT mRNA ratios in MUT1a cells, as measured by the allele-sensitive Taqman S34F/WT SNP assay. (B). The indicated U2AF1 shRNAs reduced total U2AF1 mRNA levels. (C, D). Reduction of U2AF1 mRNA does not affect the inclusion of cassette exons in USP25 and AXL mRNAs. Both of the cassette exons showed increased inclusion in the presence of U2AF1S34F (S1 Table). (E). Reduction of total U2AF1 mRNA favors the selection of the isoform without the 5' extended exon (“short” isoform) in a competing 3′ splice site event in SLC25C2 mRNA in both WT1 and MUT1a cells. Selection of the isoform containing the 5' extended exon (“long” isoform) was shown to be dependent on U2AF1 in cells expressing only wild-type U2AF1 [24]. The cartoon above each panel depicts the type of alternative splicing being measured. Asterisks represent statistically significant changes compared to shScbr-transduced conditions in respective cell lines. Error bars represent s.e.m (n = 3). (PDF) [file pgen.1006384.s008.pdf]

**A**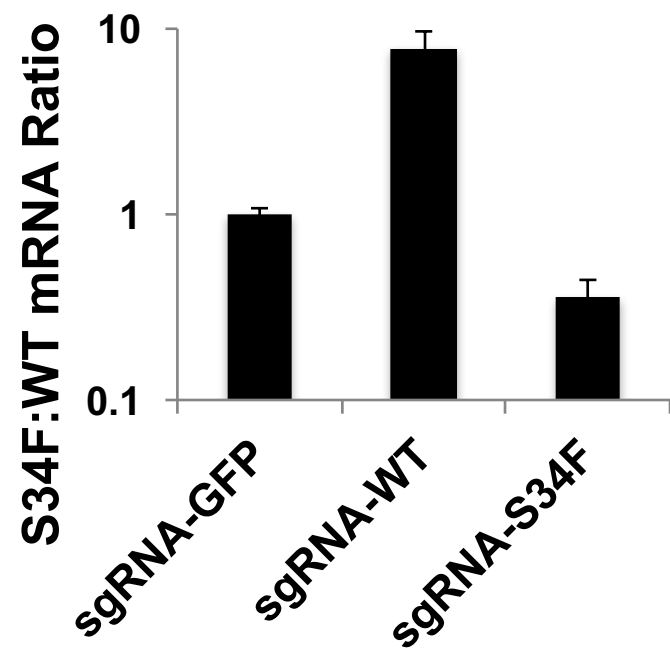**B**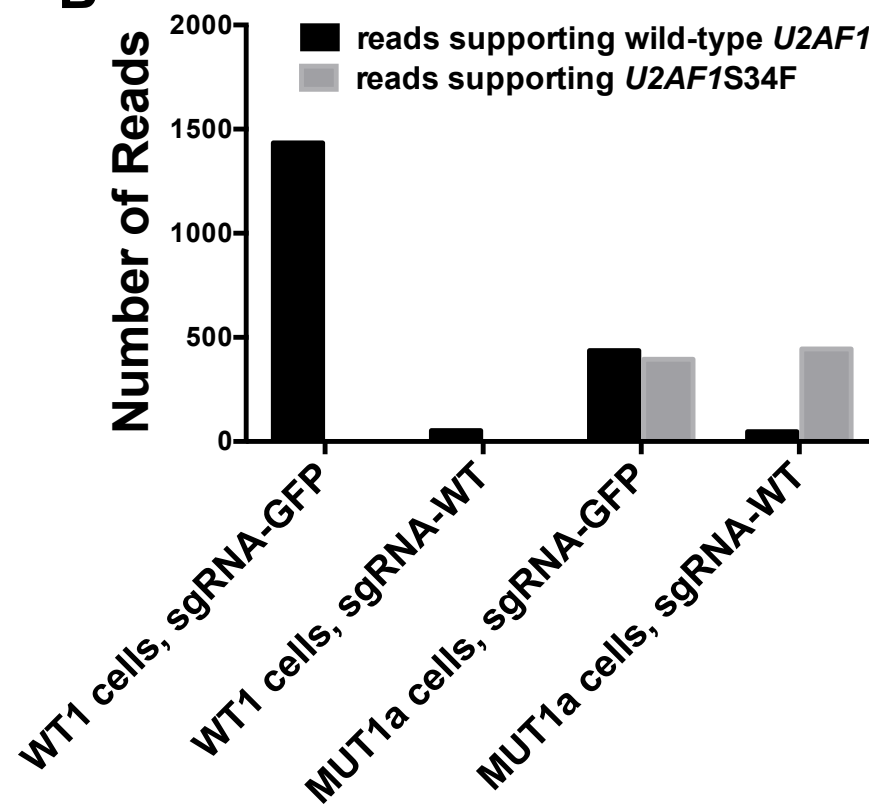

Supplement: S9 Fig — MUT1a cells were transduced with lentiviruses expressing Cas9 and either sgRNA-GFP, sgRNA-WT, or sgRNA-S34F. Cells were harvested 6 days later for (panel A) quantifying the S34F:WT mRNA ratios by the allele-sensitive S34F/WT SNP Taqman assay or (panel B) RNAseq for counting the number of reads supporting either wild-type or mutant U2AF1. Error bars represent standard deviation (s.d.) from a representative experiment in panel A. (PDF) [file pgen.1006384.s010.pdf]

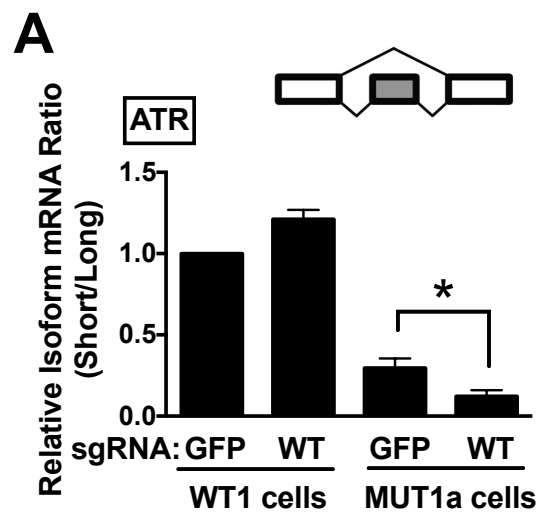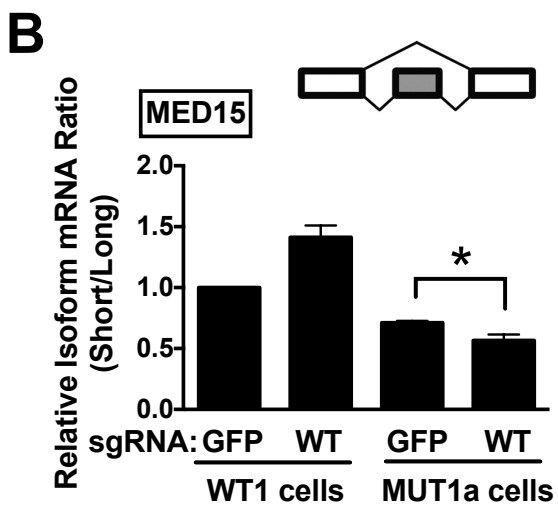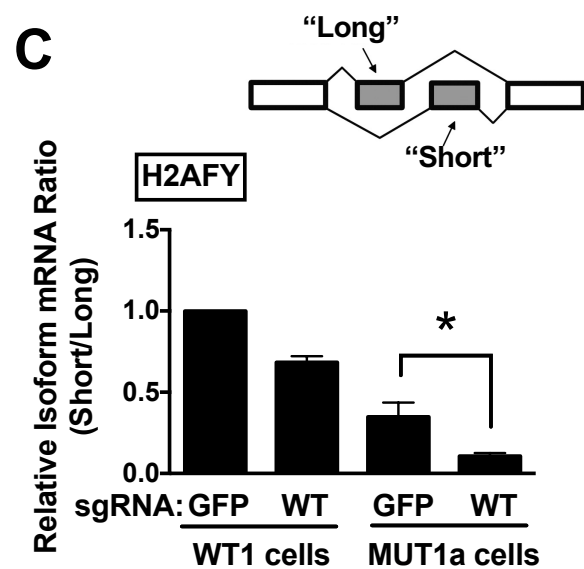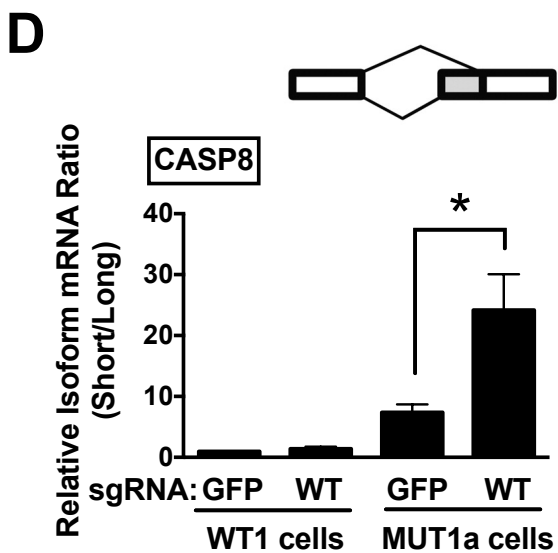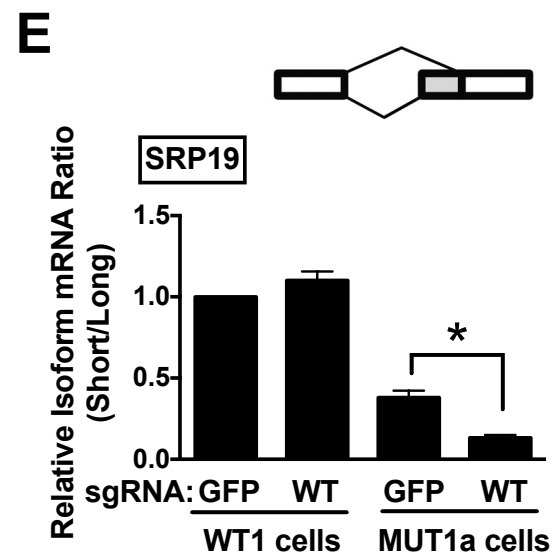

Supplement: S11 Fig — Samples from the experiment shown in Fig 3C were used to measure the frequency of alternative splicing of selected transcripts (Panel A. ATR; Panel B. MED15; Panel C. H2AFY; Panel D. CASP8; Panel E. SRP19), using RT-qPCR with isoform-specific primers in a droplet digital PCR instrument. The cartoon above each panel depicts the type of alternative splicing being measured. The relative isoform ratio in control WT1 cells (transduced with sgRNA-WT and Cas9) is set to one. Student’s t test was used for comparing the difference in the indicated comparisons. Asterisks represent statistically significant changes. Error bar represents s.e.m. (n = 3). (PDF) [file pgen.1006384.s012.pdf]

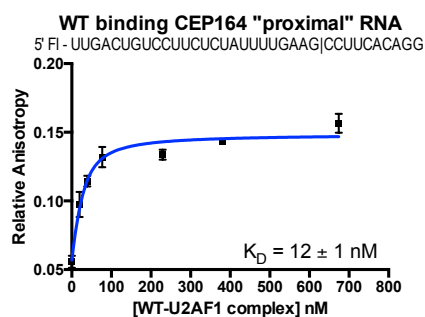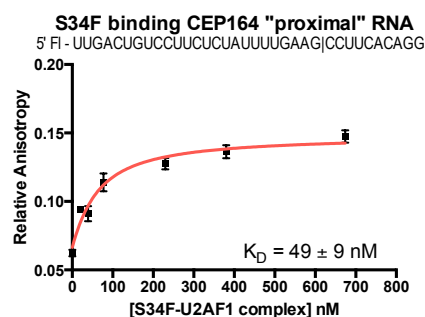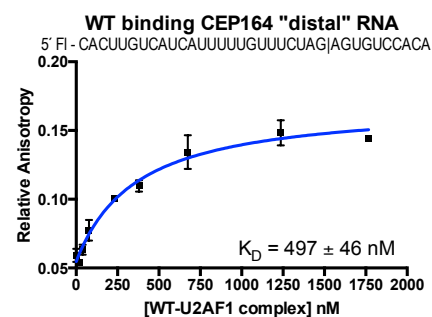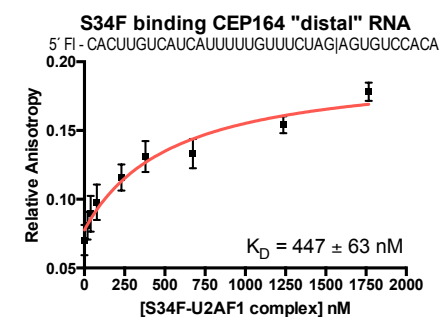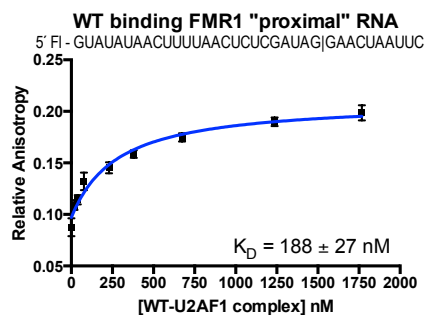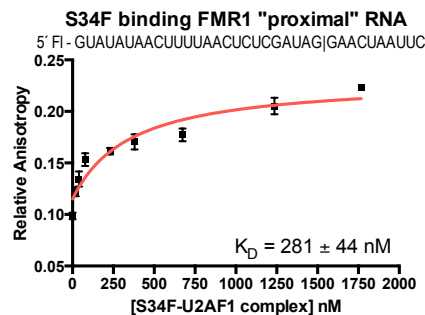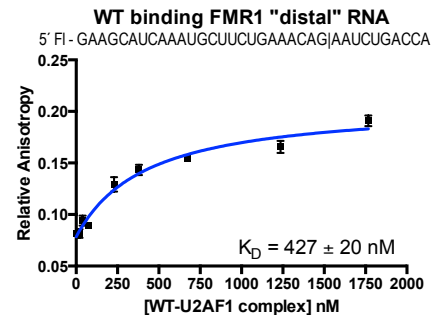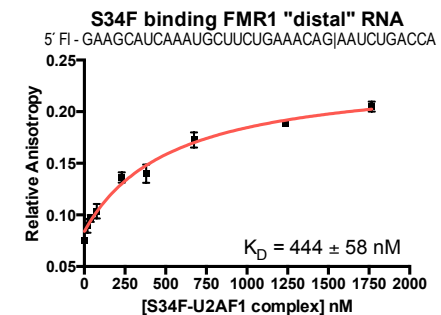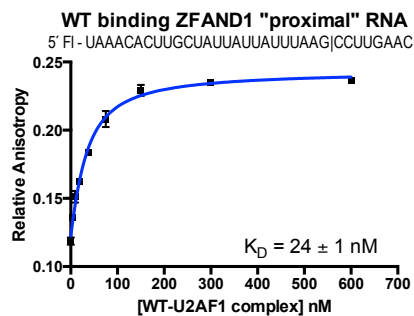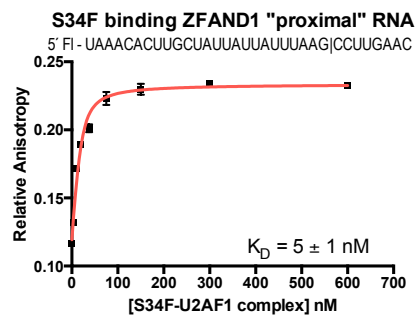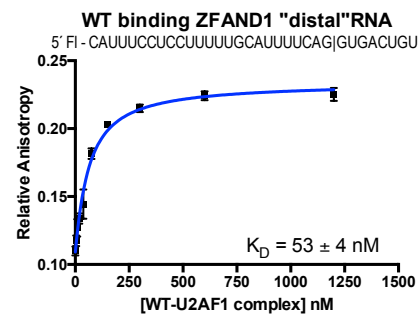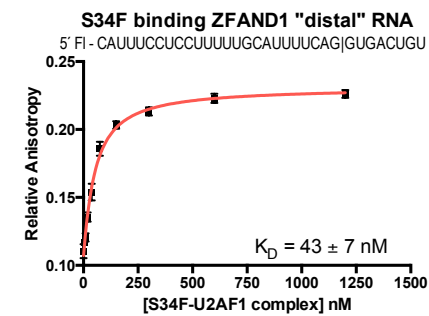

**(to be continued on the next page)**

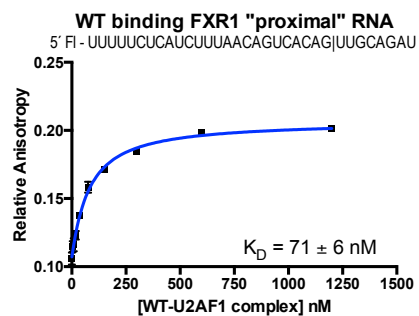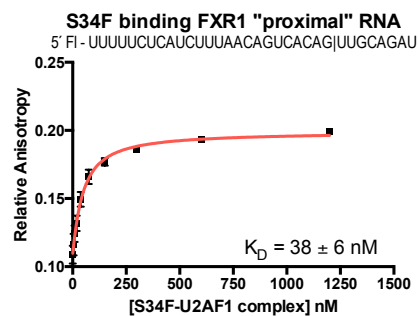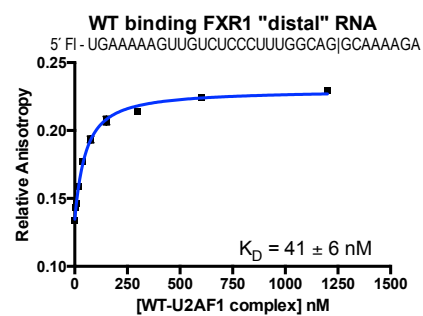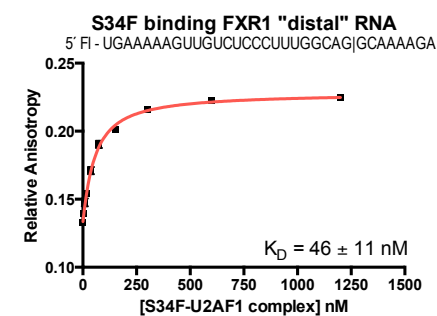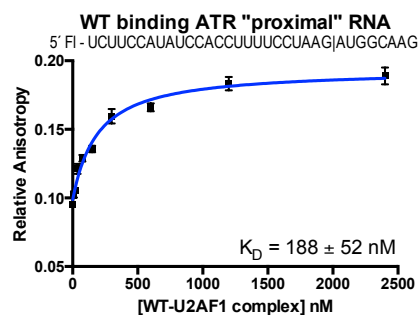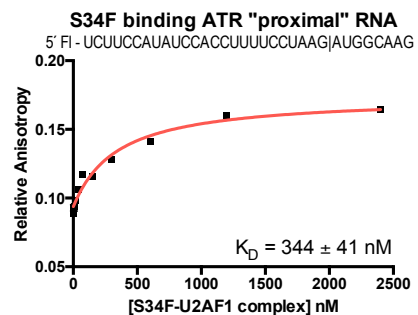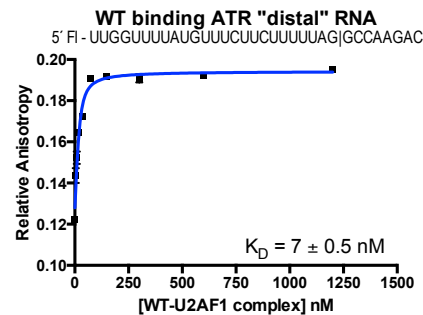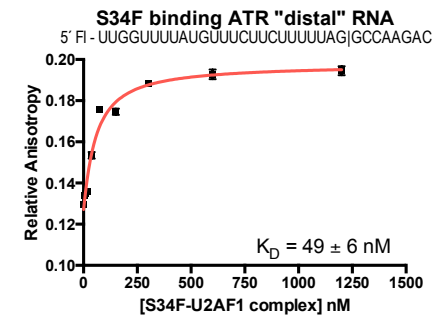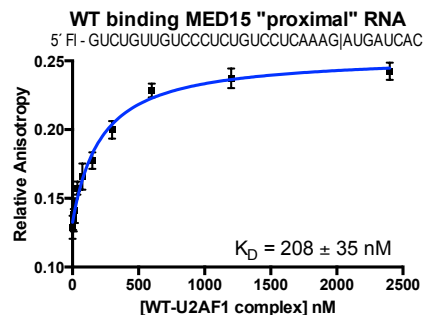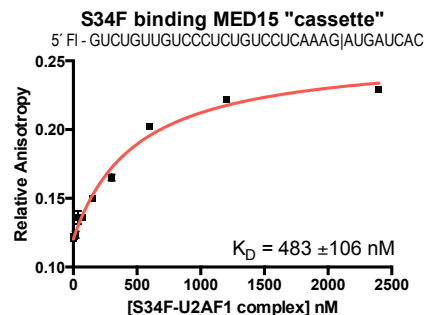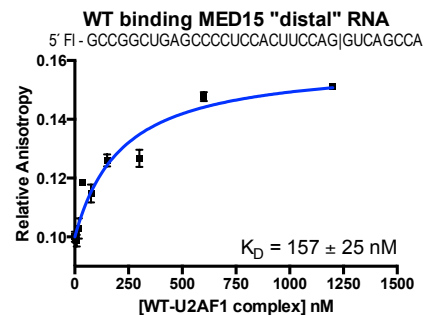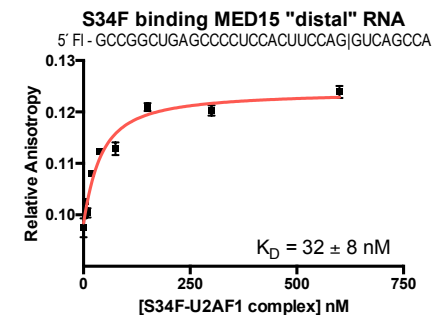

Supplement: S12 Fig — The wild-type or mutant (S34F) U2AF1 protein complexes with U2AF2 and SF1 were titrated into 5' fluorescein-tagged RNA oligonucleotides over the indicated range of concentrations. The average fluorescence anisotropy data points and error bars of three independent titrations are overlaid with the nonlinear fits as described in the Supplemental Materials and Methods. The RNA sequences are indicated below each graph title. The average values and standard deviations of the apparent equilibrium dissociation constants (KD) that resulted from three replicates are inset. (PDF) [file pgen.1006384.s013.pdf]

**A** Number Of Live Cells

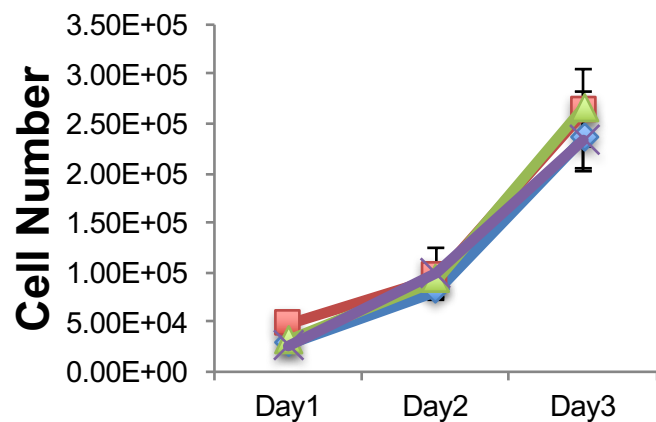

**B** Number Of Dead Cells

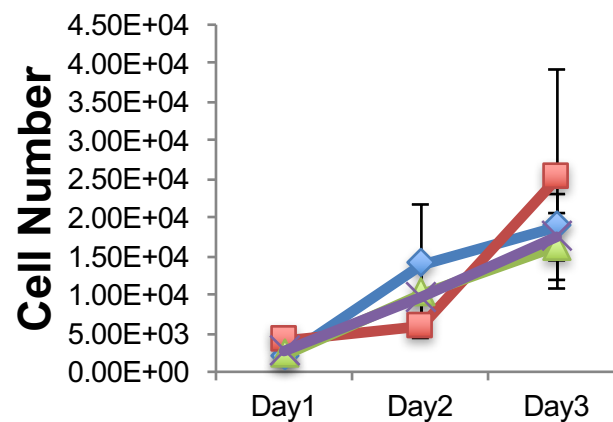

**C** % Viability

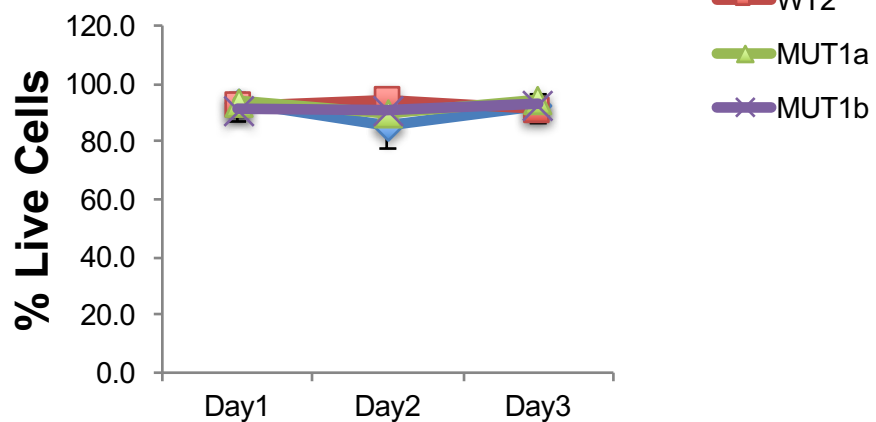

Supplement: S13 Fig — (A, B). Equal numbers of isogenic HBEC cells (WT1, WT2, MUT1a and MUT1b) were plated and the numbers of live and dead cells were quantified over the course of three days. Cell viability was determined by Trypan Blue staining. (C). Cell viability was calculated as the ratio of dead versus live cells. Error bars represent s.d. from a representative experiment. (PDF) [file pgen.1006384.s014.pdf]

**A**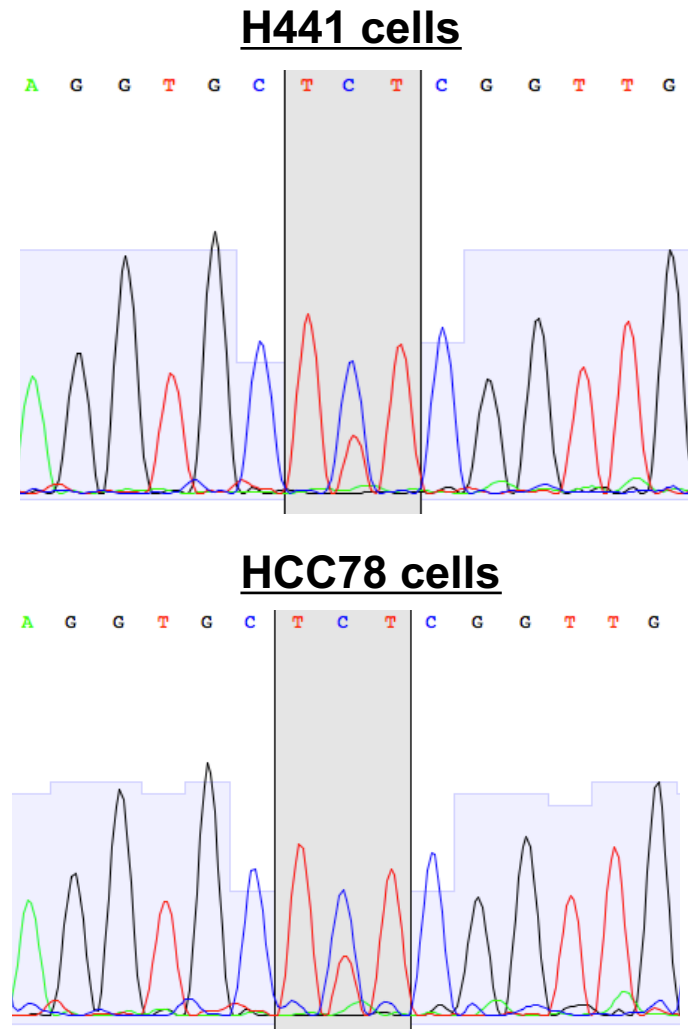**B**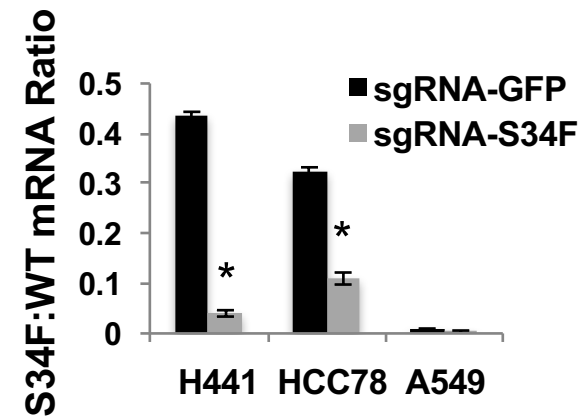**C**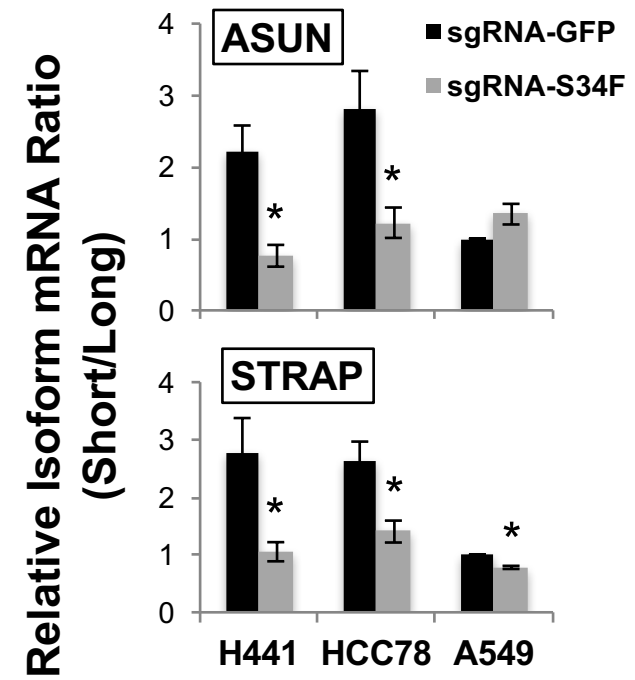

Supplement: S14 Fig — (A). U2AF1S34F is the minor allele in H441 and HCC78 lung adenocarcinoma cell lines. Genomic PCR was performed for H441 and HCC78 cells, using a PCR primer pair that flanks the codon Serine 34 (forward, GCAAGGAAGAGGAGGTGCTTA; reverse, AAGTCGATCACCTGCCTCAC). The PCR amplicon was subject to Sanger sequencing. A region containing the codon Serine 34 (in grey) is shown. “TCT” (encoding Serine) is the wild-type sequence, while “TTT” encodes Phenylalanine. (B). Polyclonal cell lines were established for H441, HCC78 and A549 cells transduced with Cas9 and either sgRNA-GFP or sgRNA-S34F, followed by RNA extraction and RT-qPCR to determine the ratio of mutant and wild-type U2AF1 mRNA by the allele-sensitive S34F/WT SNP Taqman assay. (C). The relative short/long isoform ratios of ASUN and STRAP mRNAs were measured as in Fig 1D. The isoform ratio in A549 cells transduced with Cas9 and sgRNA-GFP was set to 1. Asterisks represent statistically significant changes compared to the respective cell lines transduced with Cas9 and sgRNA-GFP. Error bars represent s.e.m. (n = 3). (PDF) [file pgen.1006384.s015.pdf]

**A**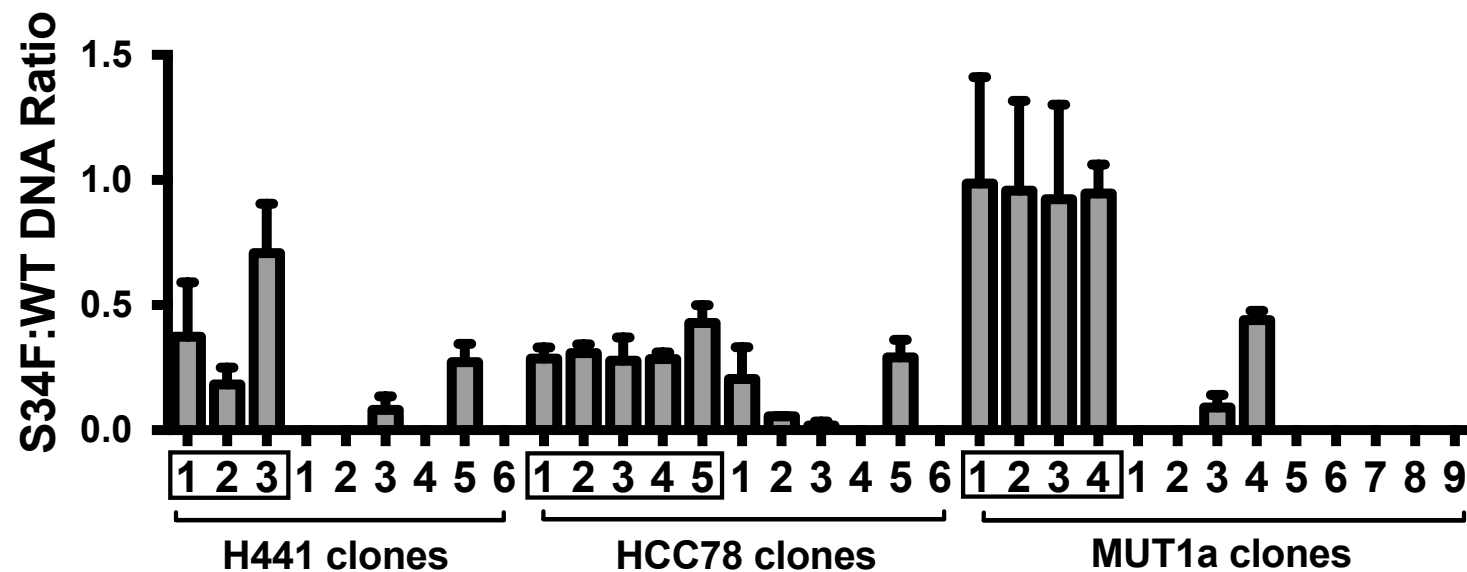**B**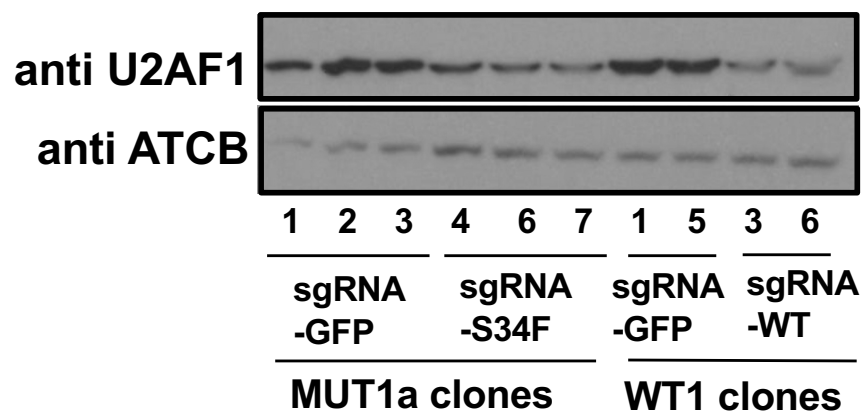**C**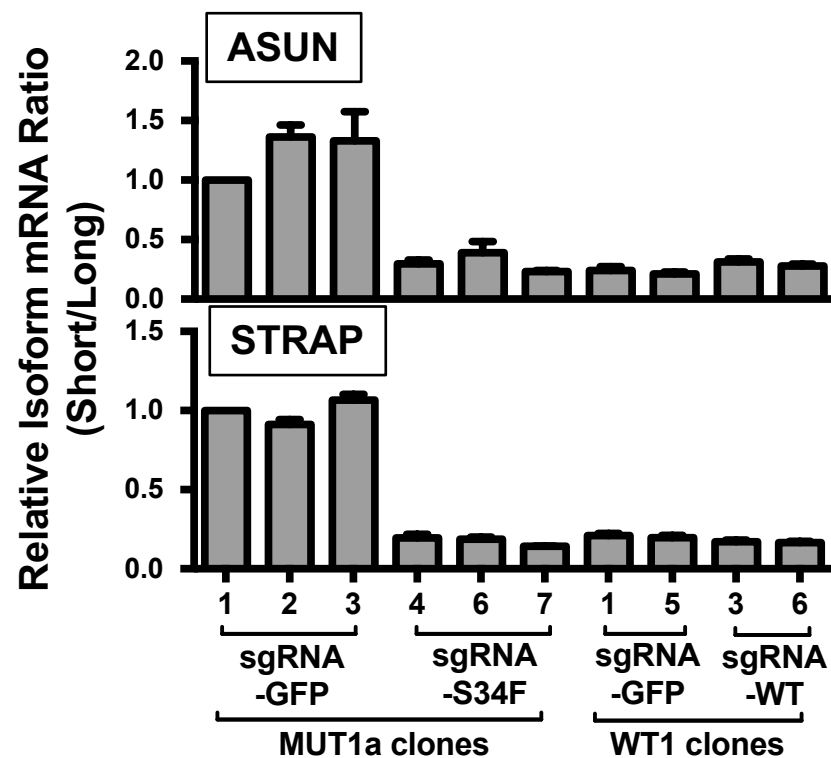

Supplement: S15 Fig — (A). The S34F:WT DNA ratios was examined in clonal cell lines derived from H441 and HCC78 LUAD lines and MUT1a cells after transduction with Cas9 and sgRNA-GFP (boxed lines) or sgRNA-S34F. The clones were expanded and the genomic DNA was harvested to measure the ratio of mutant and WT U2AF1 DNA by the allele-sensitive S34F/WT SNP Taqman assay. (B). Levels of U2AF1 and ACTB protein were measured by immunoblots in extracts from selected MUT1a-derived cell clones from Panel A, as well as from two clones derived from WT1 cells. The WT1-derived cell clones lack one intact WT U2AF1 allele and were generated by transiently transfecting WT1 cells with Cas9 and sgRNA-GFP or sgRNA-WT. (C). As in Fig 2C, the isoform ratios of ASUN and STRAP mRNAs were measured in MUT1a- or WT1-derived cell clones. Error bars represent s.d. from a representative experiment. (PDF) [file pgen.1006384.s016.pdf]

**A**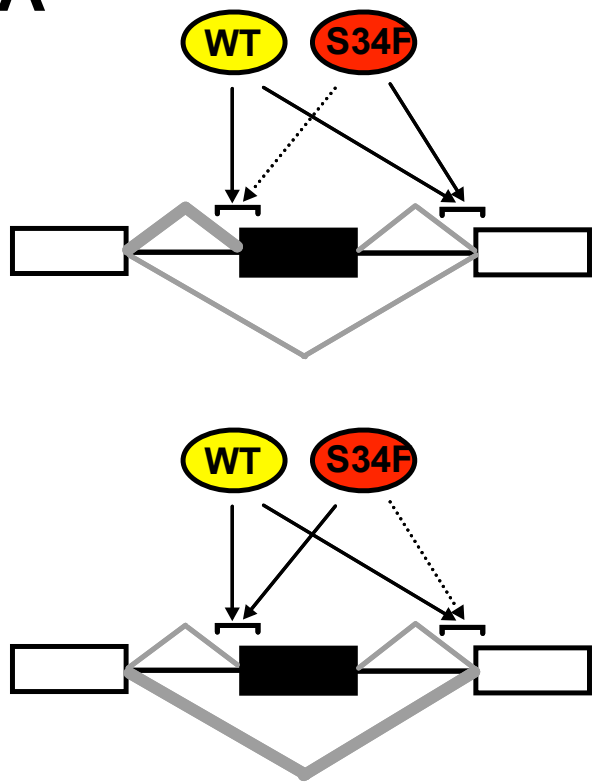**B**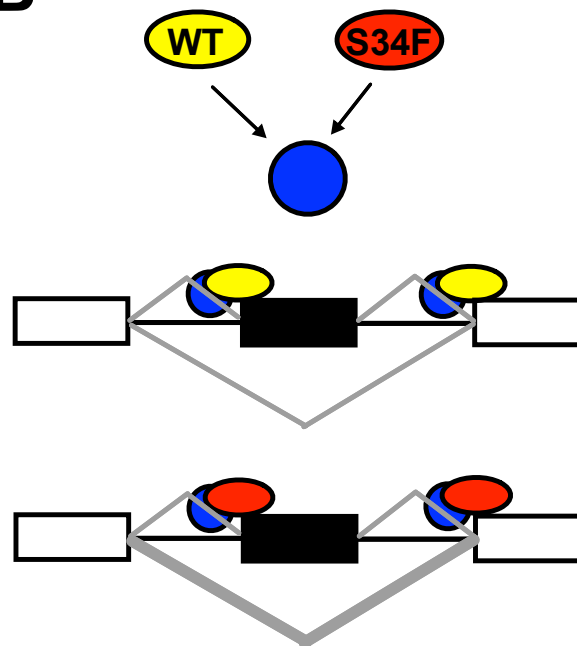

Supplement: S18 Fig — A. Competition occurs between the proximal and distal 3′ splice sites in the presence of wild-type U2AF1 and its co-factors (yellow ellipse), resulting in alternative usage of the cassette exon (black box) under steady state. Mutant U2AF1 and its co-factors (red ellipse) have different affinities for the proximal and/or distal 3′ splice sites, altering the usage of the cassette exon. The brackets indicate regions surrounding the proximal and distal 3′ splice sites. B. In this alternative mode, wild-type U2AF1 binds to a co-factor (or co-factors, represented by a blue circle) that is presented at a lower stoichiometry. The wild-type complex recognizes the 3′ splice sites to mediate the recognition of the cassette exon. Mutant U2AF1 competes with wild-type U2AF1 for this rating-limiting co-factor. Mutant U2AF1-containing complex has different affinities for the splice sites, resulting in alteration in the inclusion of the cassette exon. In these cartoons, blank boxes represent constitutive exons flanking the cassette exon. Introns are the black lines connecting the exons. Arrows with solid or dotted lines represent affinities of the splicing factors to RNA. Grey lines represent possible splices and the weight of the lines represents altered splicing efficiency. (PDF) [file pgen.1006384.s019.pdf]
