# Supplementary material for: Wild-Type U2AF1 Antagonizes the Splicing Program Characteristic of U2AF1-Mutant Tumors and Is Required for Cell Survival
Source: PLoS Genet. 2016 Oct 24;12(10):e1006384. doi: 10.1371/journal.pgen.1006384 (PMC5077151; doi:10.1371/journal.pgen.1006384)
Supplement: S4 Fig — (A). Strategy for identifying intermediate cell clones by Southern blot. Left Panel: Southern blot probes and the expected fragment sizes after restriction enzyme digestion of genomic DNA. Right Panel: Details of the Southern blot strategy. E, EcoR I; X, Xho I; E1, E2, E8, exons 1, 2, and 8 (black boxes); E2/mE2, wild-type or S34F-mutant exon 2; yellow box, drug selection cassette (PGK-Hygro∆TK) flanked by inverted terminal repeat sequences for recognition by the Piggybac transposase (green lines); thick black lines, Southern blot probes. (B). Drug-resistant intermediate clones were identified by Southern blot. Cell clones with the expected pattern of restriction fragments are marked with a star. (C). Top panel: Strategy for identifying mutant and wild-type intermediate clones by PCR amplicon sequencing. The PCR primer P1-F is upstream of the 5' homology arm sequence from the donor vector, while P1-R is within the drug selection cassette. Therefore, the PCR amplicon is specific for the intermediate allele. Bottom Panel: Sanger sequencing results show that one of the three intermediate clones, #21, carried the S34F mutation, while clones #12 and #16 were wild-type for U2AF1. The codons (on the reverse strand) for Ser34 or Phe34 are underlined. (D). The relationship of the intermediate and final cell clones. (E). U2AF1 mRNA and protein levels were similar in the final cell clones. Top Panel: U2AF1 mRNA was measured by RT-qPCR and normalized to levels of GAPDH mRNA. The relative U2AF1 mRNA level in WT1 cells was set to 1.0. (Bottom) Immunoblots for U2AF1 and ACTB using total cell lysates from the indicated cell lines. (PDF) [file pgen.1006384.s005.pdf]

A

Southern Blot Strategy

	5' Probe (Xho I cut)	3' Probe (EcoR I cut)
Wild type allele	3.3 kb	10.7 kb
Intermediate allele	6.1 kb	3.0 kb

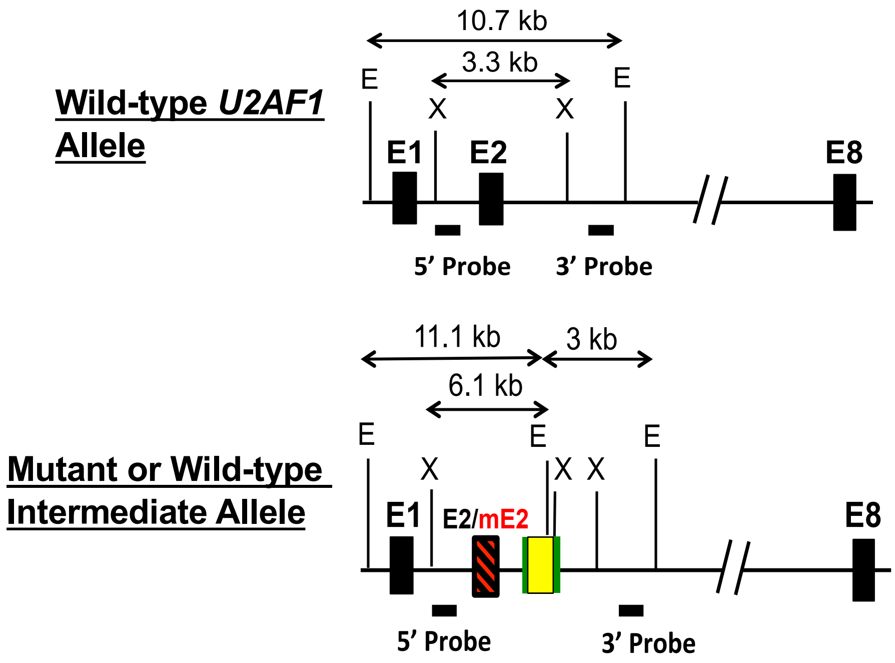

B

Southern Blot

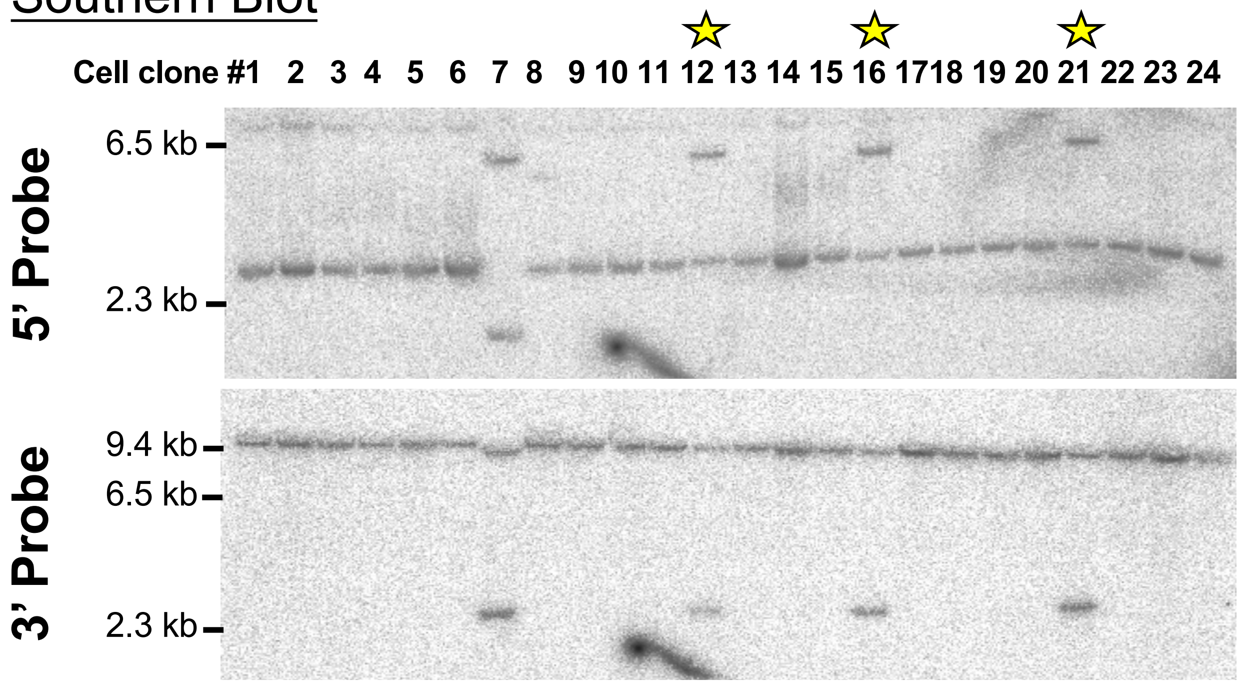

(to be continued on the next page)

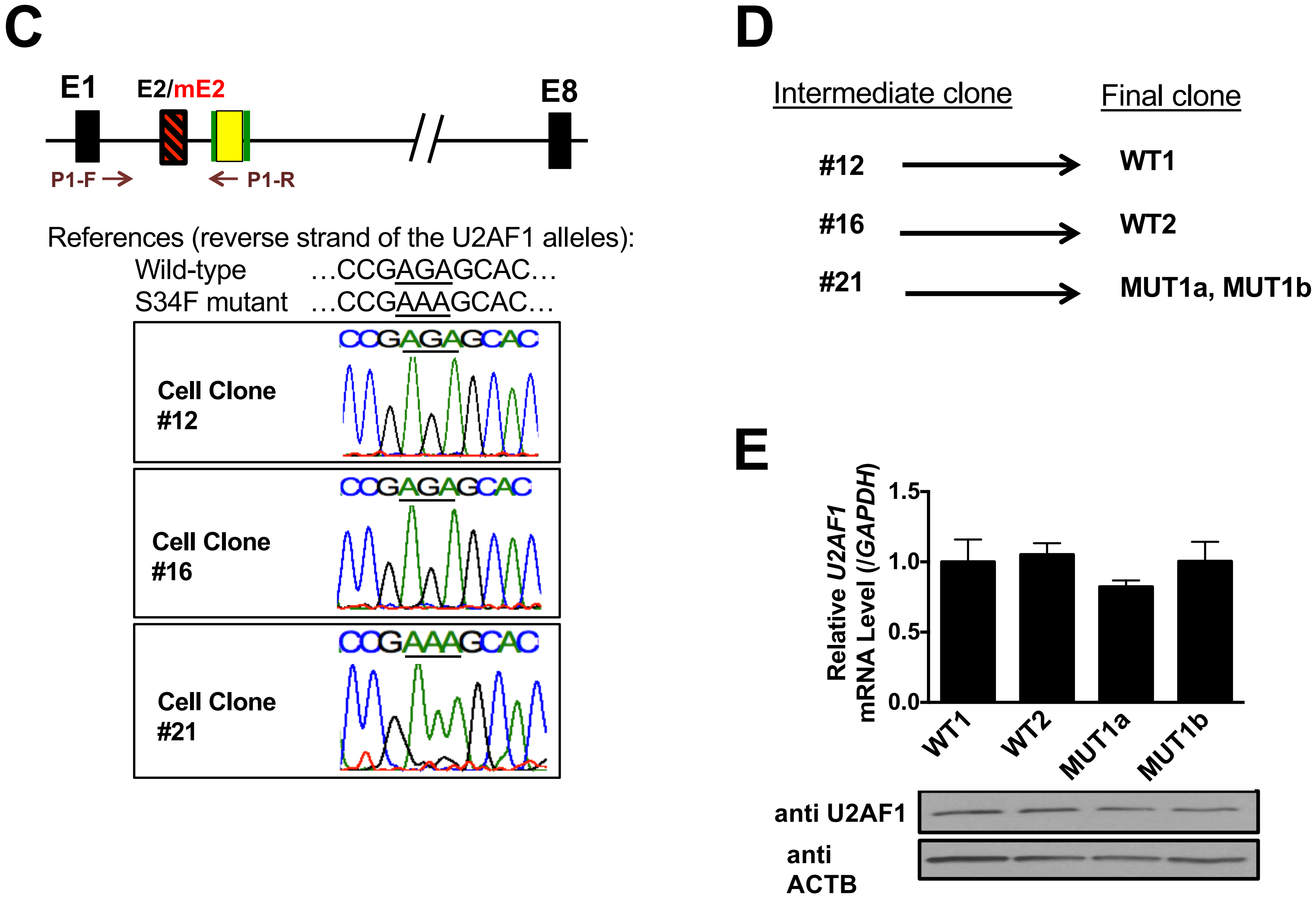
C
E1
E2/mE2
E8
P1-F
P1-R
References (reverse strand of the U2AF1 alleles):
Wild-type ...CCGAGAGCAC...
S34F mutant ...CCGAAAGCAC...
CCGAGAGCAC
Cell Clone #12
CCGAGAGCAC
Cell Clone #16
CCGAAAGCAC
Cell Clone #21
D
Intermediate clone
Final clone
#12
WT1
#16
WT2
#21
MUT1a, MUT1b
E
Relative U2AF1 mRNA Level (/GAPDH)
1.5
1.0
0.5
0.0
WT1
WT2
MUT1a
MUT1b
anti U2AF1
anti ACTB
