# Supplementary material for: Wild-Type U2AF1 Antagonizes the Splicing Program Characteristic of U2AF1-Mutant Tumors and Is Required for Cell Survival
Source: PLoS Genet. 2016 Oct 24;12(10):e1006384. doi: 10.1371/journal.pgen.1006384 (PMC5077151; doi:10.1371/journal.pgen.1006384)
Supplement: S5 Fig — (A). Upper Panel: Sequences of the primers and probes used in the allele-sensitive S34F/WT SNP Taqman assay. The nucleotide corresponding to the S34F missense mutation (reverse strand) is underlined. Bottom Panel: Diagram to show that all the primers (arrows) and probes (bar) are located within exon 2, allowing detection of the S34F:WT U2AF1 ratio for both genomic DNA and mRNA. (B). The S34F and wild-type (WT) probes are specific for their targets. Characterization of probe specificity was performed using plasmid DNA carrying either the WT or S34F mutant exon 2 of U2AF1. Eight 10-fold serial dilutions of the plasmid DNA templates were used (starting concentration: 2 x 10^8 molecules per 10 μl reaction), and the assay was performed in triplicate. According to the qPCR amplification curves (delta Rn vs. cycle), the WT probe is specific for wild-type U2AF1, while the S34F probe can detect wild-type U2AF1 at a lower efficiency (a mean difference of 3.5 cycles (equivalent to 11-fold) for DNA templates at the same concentration). (C). The mRNA levels of S34F and wild-type U2AF1 are similar in MUT1a and MUT1b cells, as revealed by the allele-sensitive S34F/WT SNP Taqman Assay (the S34F:WT mRNA ratio approximated 1). This result is consistent with the RNA-seq result in Fig 2B. (PDF) [file pgen.1006384.s006.pdf]

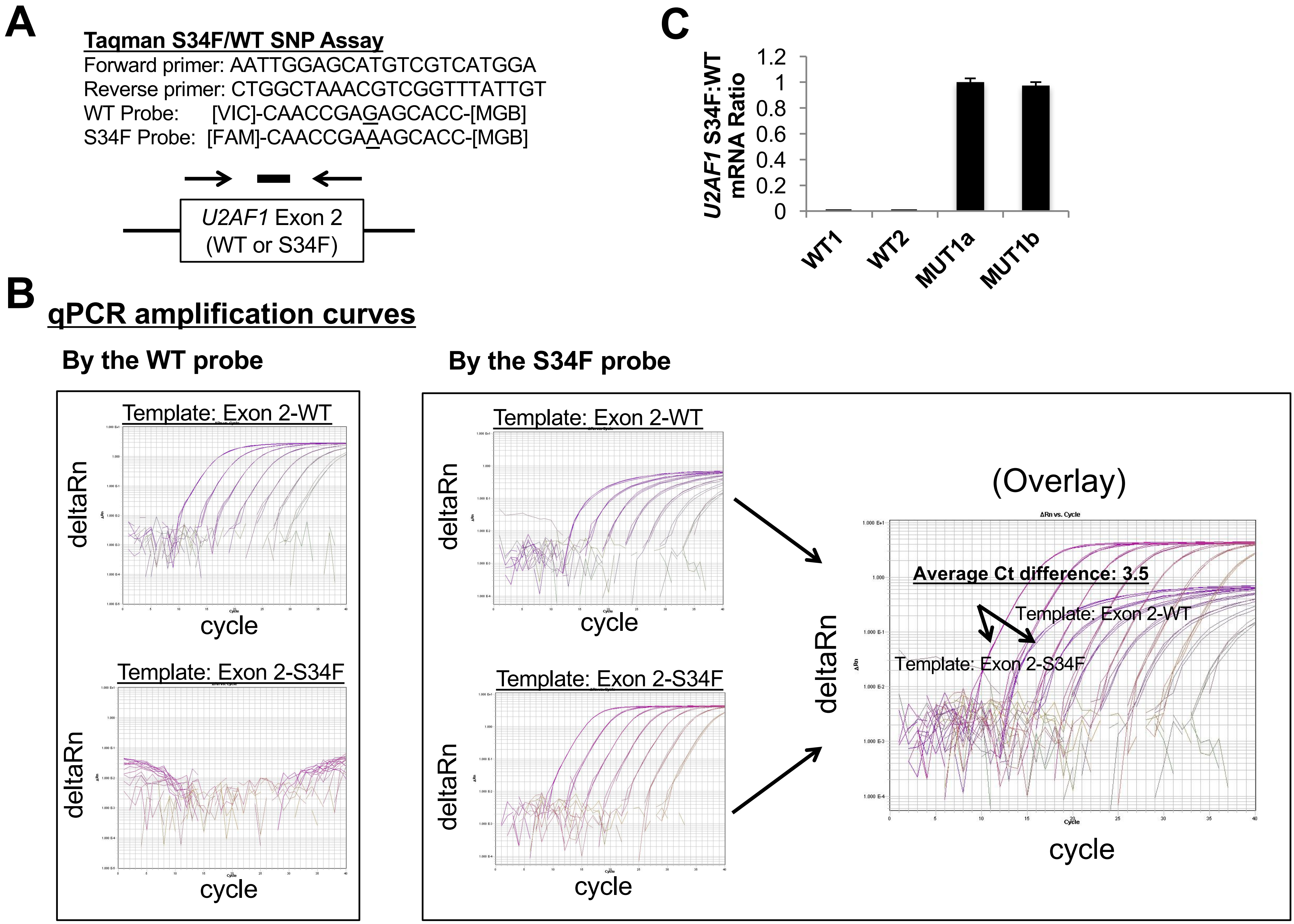
A
Taqman S34F/WT SNP Assay
Forward primer: AATTGGAGCATGTCGTCATGGA
Reverse primer: CTGGCTAAACGTCGGTTTATTGT
WT Probe: [VIC]-CAACCGAGAGCACC-[MGB]
S34F Probe: [FAM]-CAACCGAAAGCACC-[MGB]
U2AF1 Exon 2
(WT or S34F)
B
qPCR amplification curves
By the WT probe
Template: Exon 2-WT
deltaRn
cycle
Template: Exon 2-S34F
deltaRn
cycle
By the S34F probe
Template: Exon 2-WT
deltaRn
cycle
Template: Exon 2-S34F
deltaRn
cycle
(Overlay)
ΔRn vs. Cycle
Average Ct difference: 3.5
Template: Exon 2-WT
Template: Exon 2-S34F
deltaRn
cycle
C
U2AF1 S34F:WT mRNA Ratio
1.2
1
0.8
0.6
0.4
0.2
0
WT1
WT2
MUT1a
MUT1b
