# Supplementary material for: Wild-Type U2AF1 Antagonizes the Splicing Program Characteristic of U2AF1-Mutant Tumors and Is Required for Cell Survival
Source: PLoS Genet. 2016 Oct 24;12(10):e1006384. doi: 10.1371/journal.pgen.1006384 (PMC5077151; doi:10.1371/journal.pgen.1006384)
Supplement: S8 Fig — DNA sequences encoding sgRNA-WT and sgRNA-S34F are identical to human U2AF1 genomic DNA except at the middle position of the S34 codon (underlined) in the mutant sequence. The CGG sequence (in red) served as the protospacer adjacent motif (PAM) for CRISPR-Cas9. The corresponding mouse U2af1 genomic sequence, which was used to construct the U2AF1 cDNA for overexpression (Fig 3B) and rescue assays (Fig 6B), is also shown. The two differences between the mouse and human sequences are marked in grey in the mouse sequence. (PDF) [file pgen.1006384.s009.pdf]

```
                                   sgRNA-WT 5´----------GTCATGGAGACAGGTGCTCT----------3´
                                 sgRNA-S34F 5´----------GTCATGGAGACAGGTGCTTT----------3´
 U2AF1_Human (hg19, chr21:44524445-44524484) 5´GGAGCATGTCGTCATGGAGACAGGTGCTCTCGGTTGCACA3´
U2AF1_Mouse (mm10, chr17:31654999-31655038) 5´GGAGCATGTCGTCATGGAGACAGATGTTCTCGGTTGCACA3´
```
