# Supplementary material for: Wild-Type U2AF1 Antagonizes the Splicing Program Characteristic of U2AF1-Mutant Tumors and Is Required for Cell Survival
Source: PLoS Genet. 2016 Oct 24;12(10):e1006384. doi: 10.1371/journal.pgen.1006384 (PMC5077151; doi:10.1371/journal.pgen.1006384)
Supplement: S10 Fig — The analysis of experiments shown in Fig 4, Panels A and B, was extended to several different types of alterative splicing events including competing 5' splice sites, competing 3′ splice sites, retained introns and other types of alternative splicing described in S1 Table. Events with increased or decreased PSI (Percent Spliced In) are shown separately in panel A or B. Cassette exon events (displayed alone in Fig 4, panels A and B) are not included in this analysis. (PDF) [file pgen.1006384.s011.pdf]

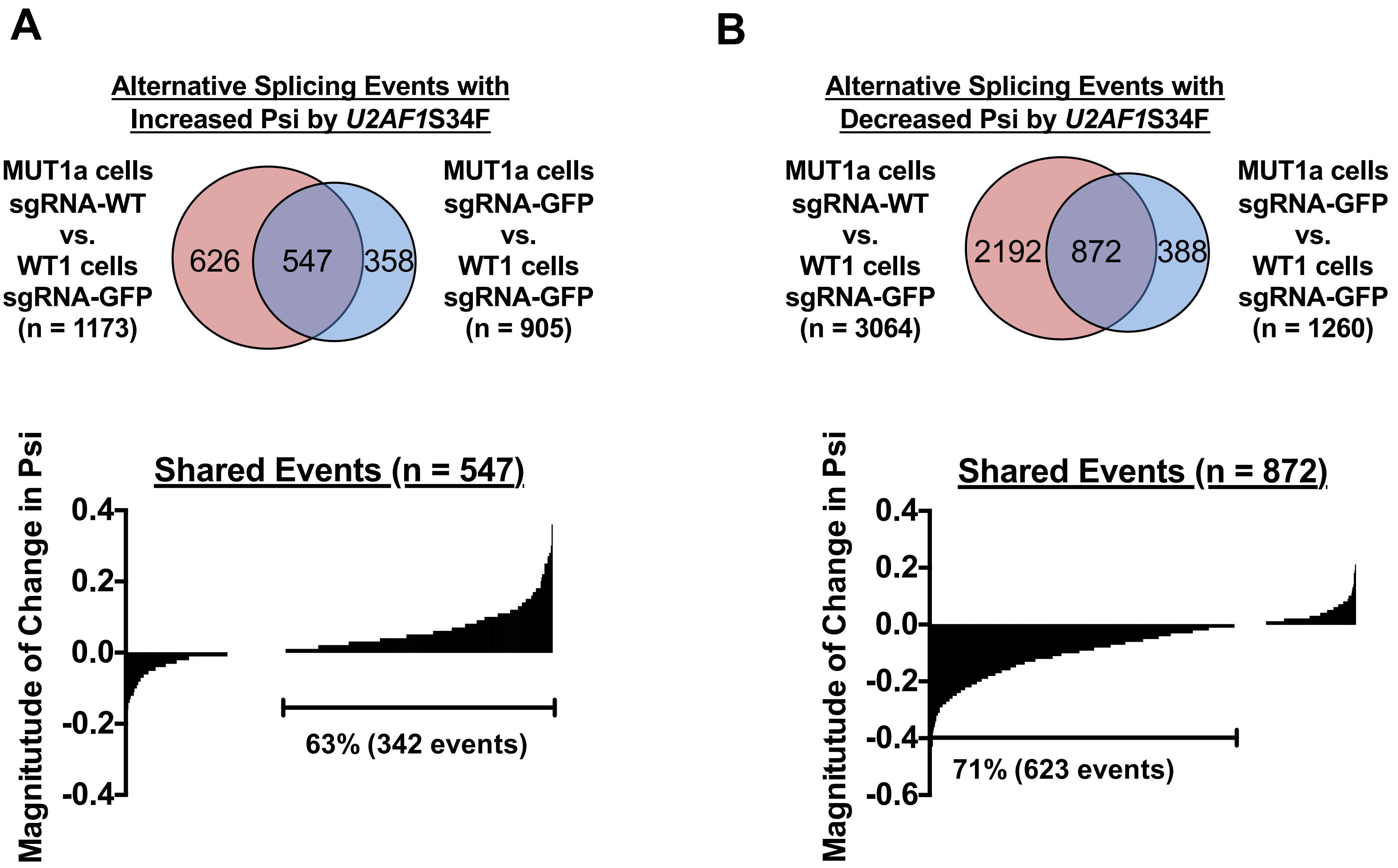
A
Alternative Splicing Events with
Increased Psi by U2AF1S34F
MUT1a cells
sgRNA-WT
vs.
WT1 cells
sgRNA-GFP
(n = 1173)
626
547
358
MUT1a cells
sgRNA-GFP
vs.
WT1 cells
sgRNA-GFP
(n = 905)
Shared Events (n = 547)
Magnitude of Change in Psi
0.4
0.2
0.0
-0.2
-0.4
63% (342 events)
B
Alternative Splicing Events with
Decreased Psi by U2AF1S34F
MUT1a cells
sgRNA-WT
vs.
WT1 cells
sgRNA-GFP
(n = 3064)
2192
872
388
MUT1a cells
sgRNA-GFP
vs.
WT1 cells
sgRNA-GFP
(n = 1260)
Shared Events (n = 872)
Magnitude of Change in Psi
0.4
0.2
0.0
-0.2
-0.4
-0.6
71% (623 events)
