# Supplementary material for: Wild-Type U2AF1 Antagonizes the Splicing Program Characteristic of U2AF1-Mutant Tumors and Is Required for Cell Survival
Source: PLoS Genet. 2016 Oct 24;12(10):e1006384. doi: 10.1371/journal.pgen.1006384 (PMC5077151; doi:10.1371/journal.pgen.1006384)
Supplement: S16 Fig — Whole transcriptome of selected H441 cell clones were measured by RNA-seq. The changes in cassette exon inclusion levels were compared as Fig 2, panels D and E. (A). Heat map depicting the inclusion levels of altered cassette exons. Dendrograms were constructed from an unsupervised cluster analysis based on all cassette exons that showed at least a 10% change in use between the indicated cell lines. (B). Sequence logos from 3′ splice sites preceding cassette exons with altered use in control H441 cells (a polyclone line transduced with Cas9 and sgRNA-GFP), comparing to H441 cells with the U2AF1S34F allele disrupted (a polyclone line transduced with Cas9 and sgRNA-S34F), display typical S34F-associated features. Logos were constructed as in Fig 1A. Comparisons of transcriptomes from the clonal lines (H441 cells sgRNA-GFP clone #1 vs. sgRNA-S34F clone #1) yield similar consensus 3′ splice site sequences. (PDF) [file pgen.1006384.s017.pdf]

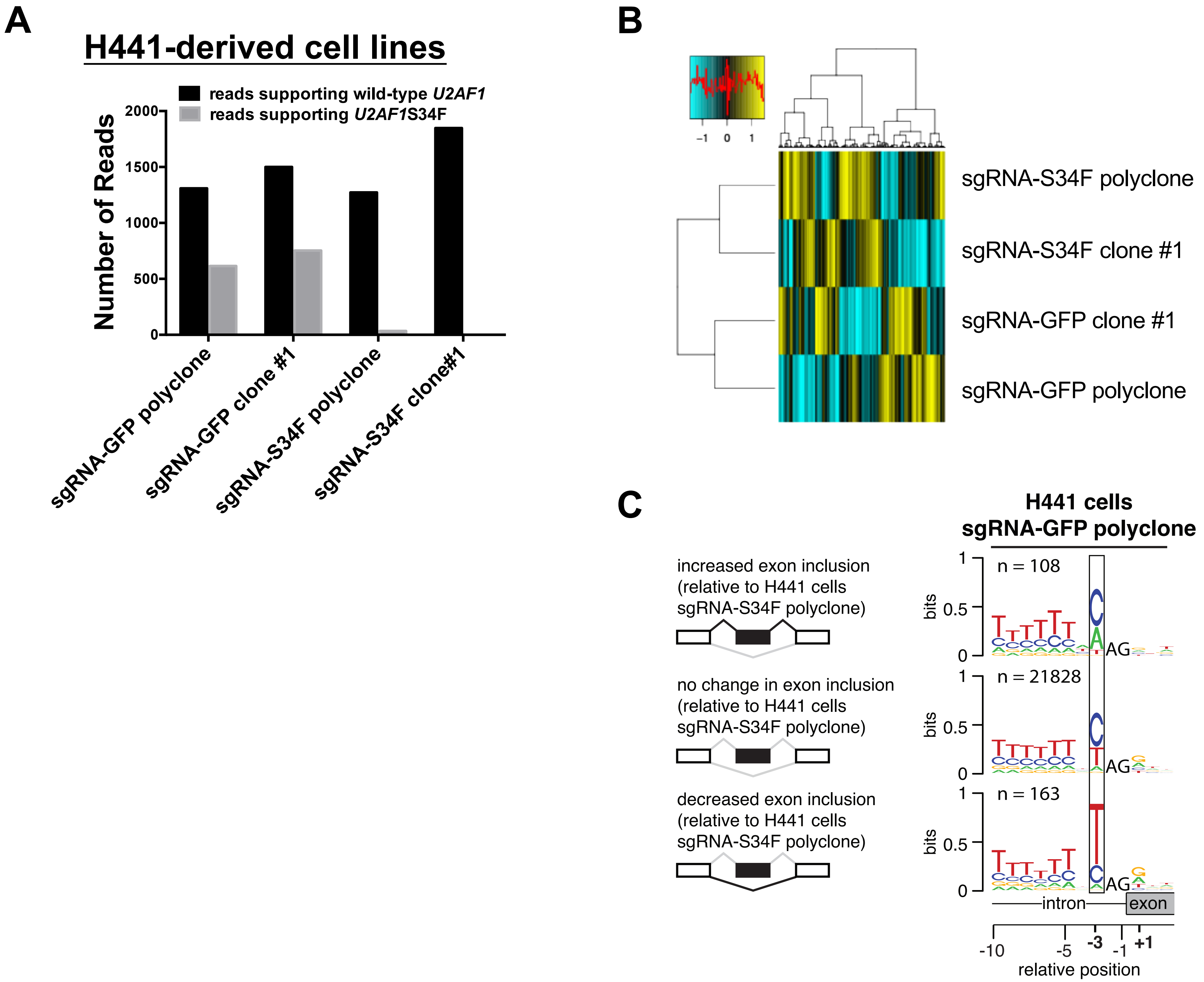

A
H441-derived cell lines
reads supporting wild-type U2AF1
reads supporting U2AF1S34F
Number of Reads
2000
1500
1000
500
0
sgRNA-GFP polyclone
sgRNA-GFP clone #1
sgRNA-S34F polyclone
sgRNA-S34F clone#1
B
−1 0 1
sgRNA-S34F polyclone
sgRNA-S34F clone #1
sgRNA-GFP clone #1
sgRNA-GFP polyclone
C
H441 cells
sgRNA-GFP polyclone
increased exon inclusion
(relative to H441 cells
sgRNA-S34F polyclone)
no change in exon inclusion
(relative to H441 cells
sgRNA-S34F polyclone)
decreased exon inclusion
(relative to H441 cells
sgRNA-S34F polyclone)
n = 108
n = 21828
n = 163
bits
1
0.5
0
AG
intron
exon
-10
-5
-3
-1
+1
relative position
