# Supplementary material for: Wild-Type U2AF1 Antagonizes the Splicing Program Characteristic of U2AF1-Mutant Tumors and Is Required for Cell Survival
Source: PLoS Genet. 2016 Oct 24;12(10):e1006384. doi: 10.1371/journal.pgen.1006384 (PMC5077151; doi:10.1371/journal.pgen.1006384)
Supplement: S17 Fig — Same as S16 Fig but selected HCC78-derived cell clones were used. (PDF) [file pgen.1006384.s018.pdf]

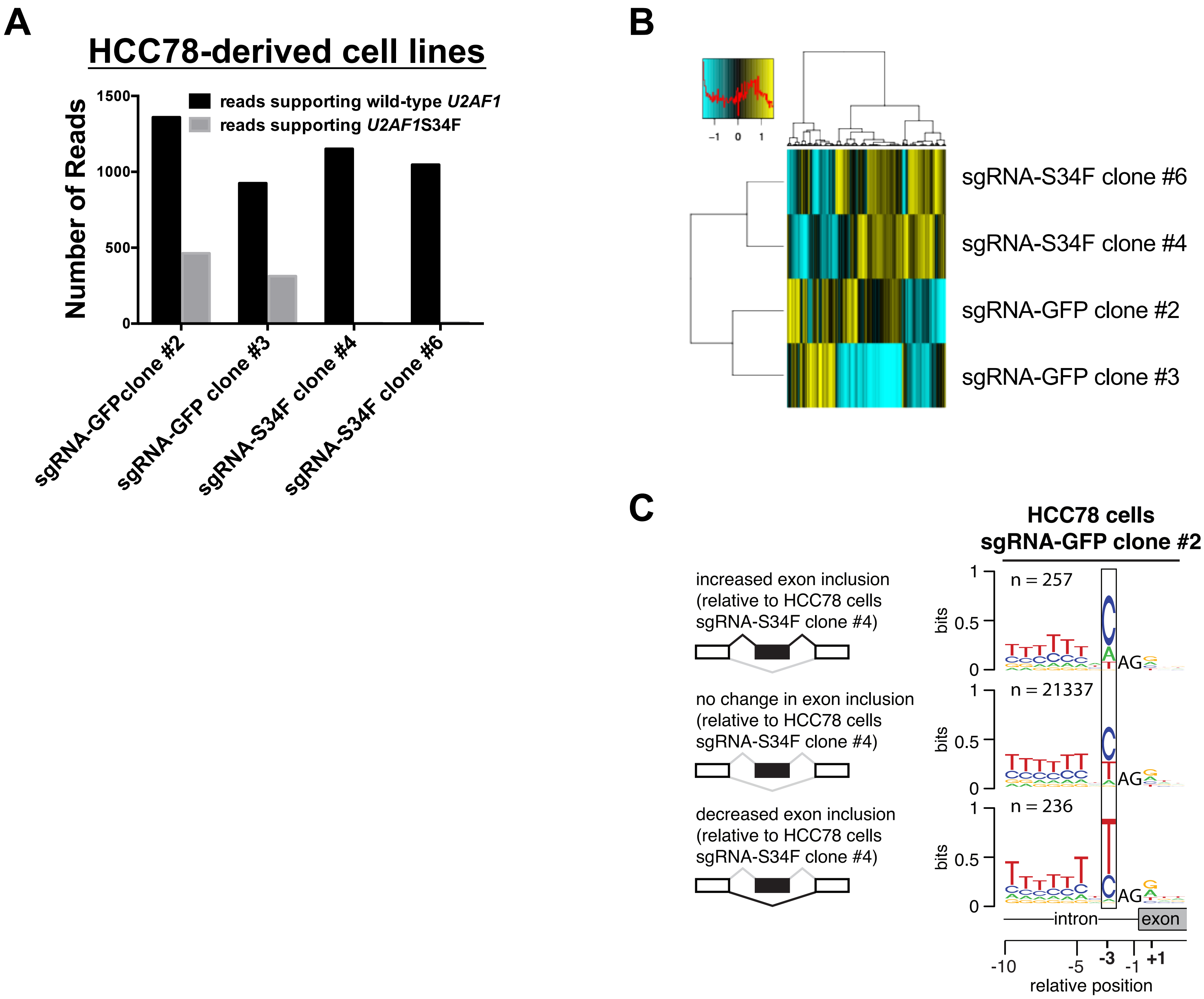
A
HCC78-derived cell lines
Number of Reads
1500
1000
500
0
reads supporting wild-type U2AF1
reads supporting U2AF1S34F
sgRNA-GFPclone #2
sgRNA-GFP clone #3
sgRNA-S34F clone #4
sgRNA-S34F clone #6
B
-1
0
1
sgRNA-S34F clone #6
sgRNA-S34F clone #4
sgRNA-GFP clone #2
sgRNA-GFP clone #3
C
HCC78 cells
sgRNA-GFP clone #2
increased exon inclusion
(relative to HCC78 cells
sgRNA-S34F clone #4)
no change in exon inclusion
(relative to HCC78 cells
sgRNA-S34F clone #4)
decreased exon inclusion
(relative to HCC78 cells
sgRNA-S34F clone #4)
n = 257
n = 21337
n = 236
bits
1
0.5
0
AG
intron
exon
-10
-5
-3
-1
+1
relative position
